# Introduction to the BioChemical Library (BCL): An Application-Based Open-Source Toolkit for Integrated Cheminformatics and Machine Learning in Computer-Aided Drug Discovery

**DOI:** 10.3389/fphar.2022.833099

**Published:** 2022-02-21

**Authors:** Benjamin P. Brown, Oanh Vu, Alexander R. Geanes, Sandeepkumar Kothiwale, Mariusz Butkiewicz, Edward W. Lowe, Ralf Mueller, Richard Pape, Jeffrey Mendenhall, Jens Meiler

**Affiliations:** ^1^ Chemical and Physical Biology Program, Medical Scientist Training Program, Center for Structural Biology, Vanderbilt University, Nashville, TN, United States; ^2^ Department of Chemistry, Center for Structural Biology, Vanderbilt University, Nashville, TN, United States; ^3^ Department of Chemistry, Departments of Pharmacology and Biomedical Informatics, Center for Structural Biology, Vanderbilt University, Nashville, TN, United States; ^4^ Institute for Drug Discovery, Leipzig University Medical School, Leipzig, Germany

**Keywords:** drug discovery, drug design, cheminformatics, open-source, deep neural network, QSAR, biochemical library, BCL

## Abstract

The BioChemical Library (BCL) cheminformatics toolkit is an application-based academic open-source software package designed to integrate traditional small molecule cheminformatics tools with machine learning-based quantitative structure-activity/property relationship (QSAR/QSPR) modeling. In this pedagogical article we provide a detailed introduction to core BCL cheminformatics functionality, showing how traditional tasks (e.g., computing chemical properties, estimating druglikeness) can be readily combined with machine learning. In addition, we have included multiple examples covering areas of advanced use, such as reaction-based library design. We anticipate that this manuscript will be a valuable resource for researchers in computer-aided drug discovery looking to integrate modular cheminformatics and machine learning tools into their pipelines.

## Introduction

Computer-aided drug discovery (CADD) methods are routinely employed to improve the efficiency of hit identification and lead optimization (Macalino et al., 2015; Usha et al., 2017). The importance of *in silico* methods in drug discovery is exemplified by the multitude of cheminformatics tools available today. These tools frequently include capabilities for tasks such as high-volume molecule processing (Hassan et al., 2006; SciTegic, 2007), ligand-based (LB) small molecule alignment (Labute et al., 2001; Jain Ajay, 2004; Chan, 2017; Brown et al., 2019), conformer generation (Cappel et al., 2015; Kothiwale et al., 2015; Friedrich et al., 2017a, 2019), pharmacophore modeling (Hecker et al., 2002; Acharya et al., 2011; Vlachakis et al., 2015), structure-based (SB) protein-ligand docking (Friesner et al., 2004; Meiler and Baker, 2006; Davis and Baker, 2009; Hartmann et al., 2009; Morris et al., 2009; Kaufmann and Meiler, 2012; Lemmon et al., 2012), and ligand design. Increasingly, modern drug discovery relies on customizable and target-specific machine learning-based quantitative structure-activity relationship (QSAR) and structure-property relationship (QSPR) modeling during virtual high-throughput screening (vHTS) (Lo et al., 2018; Vamathevan et al., 2019).

Frequently, building a drug discovery pipeline with all of these parts requires users to combine multiple different software packages into their workflow. This can be challenging because of different version requirements in package dependencies. Moreover, file- and data-type incompatibilities between packages can lead to errors and pipeline inefficiencies. Here, we describe the BioChemical Library (BCL) cheminformatics toolkit, a freely available academic open-source software package with tightly integrated machine learning-based QSAR/QSPR capabilities.

The BCL is an application-based software package programmed and compiled in C++. This means that BCL applications can be integrated into existing pipelines without the need for package dependency management (i.e., maintaining directory-dependent virtual environments, or keeping separate Miniconda environments for each task). In addition, BCL applications are modular and can be easily combined into complex protocols with simple Shell scripts. Output files from the BCL are primarily common file types that can also be read as input by other software packages. Its command-line usage will be familiar to users of the popular macromolecular modeling software Rosetta (Kaufmann et al., 2010). The simple command line user interface (UI) makes it easy to create complex protocols without extensive coding or scripting experience. Our goal with this manuscript is to describe the core functionalities of the BCL cheminformatics toolkit and provide detailed examples for real use cases. At the end, we briefly discuss ongoing software developments that may be of interest to users.

## Molecule Preparation and Processing

### Fundamentals of BioChemical Library Command-Line Syntax

The first thing to complete after downloading and installing the BCL is to add the license file to the/path/to/bcl folder. We further recommend adding/<path>/<to>/bcl to the LD_LIBRARY_PATH and PATH environment variables in the. cshrc/.bashrc. This allows users to access the BCL from any terminal window simply by typing bcl. exe into the command-line. For detailed setup instructions, read the appropriate operating system (OS)-specific ReadMe file in bcl/installer/.

The BCL is organized into application groups each of which contains multiple applications. To view the application groups and associated applications, run the BCL help command:

bcl.exe help

The BCL has application groups for cheminformatics, protein folding, machine learning, and other tasks (Supplementary Table S1). To isolate and view the applications associated with the application group molecule, for example, run the application group help command:

bcl.exe molecule:Help

Generally, the syntax to access a BCL application is as follows:

bcl.exe application_group:Application

The help menu for any application cans similarly be accessed as

bcl.exe application_group:Application --help

These help options list the basic arguments and parameters available for each application. More detailed help options are also frequently available for individual application parameters. In this way, all of the documentation required to run the BCL can be readily accessed from the command line. The application groups composing the core of the BCL cheminformatics toolkit include the following: Molecule, Descriptor, and Model (Table 1).

**TABLE 1:** Overview of BCL application groups covered in this manuscript.

Application Group	Typical Inputs	Typical Outputs
Molecule	Molecules (.sdf)	Molecules (.sdf)
Descriptor	Descriptor sets	Dataset binary file (.bin)
	Molecules (.sdf; GenerateDataset only)	Dataset comma-separated file (.csv)
	Dataset binary file (.bin)	
	Dataset comma-separated file (.csv)	
Model	Dataset binary file (.bin)	Machine learning model(s)
	Machine learning model(s)	Predictions

### Filtering

Molecules are input to the BCL in the MDL structure-data format (SDF) file. Often, molecules that are downloaded or converted from one source to another contain errors (e.g., incorrect bond order assignments, undesired protonation states/formal charge, etc.). Dataset sanitization is a critical component of computational chemistry and informatics projects. The BCL molecule: Filter application is the first step in correcting these errors or identifying molecules that cannot be easily and automatically corrected.

To see all of the options available in molecule:Filter, run the following command:

bcl.exe molecule:Filter--help

or view the supplementary material (Supplementary Table S2,S3).

For the following examples we will make use of a set of the Platinum Diverse Dataset, a subset of high-quality ligands in their protein-bound 3D conformations (Friedrich et al., 2017b).

bcl.exe molecule:Filter \
-input_filenames platinum_diverse_dataset_2017_01. sdf.gz \
-output_matched platinum_diverse_dataset_2017_01. matched.sdf.gz \
-output_unmatched platinum_diverse_dataset_2017_01. unmatched.sdf.gz \
-add_h -neutralize \
-defined_atom_types–simple \
-logger File platinum_diverse_dataset_2017_01. Filter.log

This command reads in the SDF platinum_diverse_dataset_2017_01. sdf.gz, saturates all molecules with hydrogen atoms, neutralizes any formal charges, checks to see whether the molecules have valid atom types (e.g., carbon atoms making five covalent bonds are not valid), and then checks to see whether the molecules have simple connectivity (e.g., whether they are part of a molecular complex, such as a salt). The neutralization flag identifies atoms with formal charge and tries to remove the formal charge. The default behavior allows modification of the protonation state of the atom (i.e., pH) and/or the bond order. Other options (more or less aggressive neutralization schemes) are also available and can be seen in the help menu. Adding hydrogen atoms and neutralizing charges are not required operations but are shown above to demonstrate the functionality.

All molecules that match the filter (i.e., molecules with defined atom types and are not part of molecular complexes) are output into platinum_diverse_dataset_2017_01. matched.sdf, and molecules that fail to pass the filters are output into platinum_diverse_dataset_2017_01. unmatched.sdf. In this case, all molecules pass the filter. This allows the user to review the molecules that failed the filter and choose to either fix them or continue without them.

The molecule:Filter application can also be used to separate molecules by property and/or substructure using the compare_property_values flag. For example, to filter out molecules that contain 10 or more rotatable bonds and a topological polar surface area (TPSA) less than 140 Å^2^, the following command can be used:

bcl.exe molecule:Filter \
-input_filenames platinum_diverse_dataset_2017_01. sdf.gz \
-output_matched platinum_diverse_dataset_2017_01. veber_pass.sdf.gz \
-output_unmatched platinum_diverse_dataset_2017_01. veber_fail.sdf.gz \
-compare_property_values TopologicalPolarSurfaceArea less 140 \
NRotBond less_equal 10 \
-logger File platinum_diverse_dataset_2017_01. veber.log

Of 2,859 molecules, 395 were first filtered out for have a TPSA ≥140 Å^2^, and then an additional 84 molecules that had greater than 10 rotatable bonds were filtered out. Notice that the filters are applied sequentially, and molecules must pass both filters to be output to the matched file. Alternatively, the any flag can be specified such that if a molecule meets any one of the filter criteria, then it is output to the matched file:

bcl.exe molecule:Filter \
-input_filenames platinum_diverse_dataset_2017_01. sdf.gz \
-output_matched platinum_diverse_dataset_2017_01. any_pass.sdf.gz \
-output_unmatched platinum_diverse_dataset_2017_01. any_fail.sdf.gz \
-compare_property_values TopologicalPolarSurfaceArea less 140 \
NRotBond less_equal 10 \
-any -logger File platinum_diverse_dataset_2017_01. any.log

In this example, 2,801 molecules passed at least one of the filters and only 58 were filtered out.

One may also filter based on substructure similarity. This is particularly useful if there are specific substructures that are desired or that need to be avoided. For example, aromatic amines are a well-known toxicophore and cannot be incorporated into potential druglike molecules; however, it is not uncommon to find these substructures in datasets. Here, we will filter a subset of DrugBank (Wishart et al., 2018) molecules to remove aniline-containing compounds:

bcl.exe molecule:Filter \
-input_filenames drugbank_nonexperimental.simple.sdf.gz \
-output_matched drugbank_nonexperimental.simple.anilines.sdf.gz \
-output_unmatched drugbank_nonexperimental.simple.clean.sdf.gz \
-contains_fragments_from aniline. sdf.gz \
-logger File drugbank_nonexperimental.simple.toxicity_check.log

In practice, we usually explicitly filter certain toxicophore substructures via graph search with the MoleculeTotalToxicFragments descriptor in conjunction with compare_property_values flag; however, this example illustrates the flexibility to filter by substructure similarity with molecule:Filter. In addition to the standard use cases presented here, molecule:Filter can identify molecules with clashes in 3D space, conformers outside of some tolerance value from a reference conformer, exact substructure matches, specific chemical properties, and more. Some of these filters will be further explored in other subsections.

### Removing Redundancy

Another critical aspect of dataset sanitization is removing redundancy. This is especially important when preparing datasets for QSAR model training and testing. If molecules appear more than once in a dataset, then it is possible that they could appear simultaneously in the training and test sets, leading to an artificial inflation in test set performance.

The BCL application molecule:Unique can help with this task. It has four levels at which it can compare and differentiate molecules:

1. Constitutions–compares atom identities and connectivity disregarding stereochemistry;
2. Configurations–compares atom identities, connectivity, and stereochemistry;
3. Conformations–compares configurations as well as 3D conformations;
4. Exact–checks to see whether atom identities and order are equal with the same connectivities, bond orders, stereochemistry, and 3D coordinates.

The first time the BCL encounters a molecule in an SDF it will store it in memory. Any additional encounters with the same molecule (at the chosen level described above) will be marked as duplicate encounters. The default behavior is to output only the first encounter of each molecule. There are cases in which a molecule appears multiple times but has different MDL properties and/or property values. It may not be desirable to lose the stored properties on duplicate compounds. In such cases, the user can choose to merge the properties or overwrite the duplicate descriptors instead.

For example, one may want to see if any high-throughput screening (HTS) hits have activity on multiple targets. Previously, we published nine high-quality virtual HTS (vHTS) benchmark sets for QSAR modeling binary classification tasks (Butkiewicz et al., 2013). Here, we will take a look at the active compounds from each of those nine datasets and see if any of them have activity on multiple targets.

bcl.exe molecule:Unique \
-input_filenames 1798_actives.sdf.gz 1843_actives.sdf.gz \
2258_actives.sdf.gz 2689_actives.sdf.gz \
435008_actives.sdf.gz 435034_actives.sdf.gz \
463087_actives.sdf.gz 485290_actives.sdf.gz 488997_actives.sdf.gz \
-compare Constitutions \
–output_dupes all_actives.dupes.sdf.gz \
–logger File all_actives.unique.log

The output file all_actives.dupes.sdf.gz contains 22 molecules that are active in at least two different datasets (note that each individual dataset was pre-processed to remove redundant molecules). If we want to merge the properties of these 22 compounds and isolate them from the rest of the actives, we can perform a second molecule: Unique with the merge_descriptors flag set, and then use molecule:Filter with the contains flag to isolate the duplicated compounds:

bcl.exe molecule:Unique \
-input_filenames 1798_actives.sdf.gz 1843_actives.sdf.gz \
2258_actives.sdf.gz 2689_actives.sdf.gz \
435008_actives.sdf.gz 435034_actives.sdf.gz \
463087_actives.sdf.gz 485290_actives.sdf.gz 488997_actives.sdf.gz \
-compare Constitutions–merge_descriptors \
-output all_actives.unique_merged.sdf.gz \
–logger File all_actives.unique_merged.log

followed by

bcl.exe molecule:Filter \
-input_filenames all_actives.unique_merged.sdf.gz \
-contains all_actives.dupes.sdf.gz \
-output_matched all_actives.dupes_merged.sdf.gz \
–logger File all_actives.dupes_merged.log

When merge_descriptors is passed, all unique properties are included in the resultant output file. If the same property is present on duplicates, then the first observation of that property is stored on the output molecule. If overwrite_descriptors is passed instead of merge_descriptors, the last observation of a duplicate property is stored. By default, without either of these flags only the MDL properties on the first occurrence of a molecule are stored in the output.

It may be that some of the compounds in the previous example that have activity on multiple targets are actually stereoisomers. Here, the molecules were compared based on atom identity and connectivity (Constitutions). Iterative runs of molecule:Unique coupled with molecule:Filter can be used to identify such cases.

### Sorting and Reordering

Sorting molecules is also useful during vHTS. After making predictions on a million compounds with a QSAR model, frequently users will want to identify some small top fraction of most probable hits for experimental testing. This can be readily achieved with molecule:Reorder (note–this example utilizes pseudocode for filenames):

bcl.exe molecule:Reorder \
-input_filenames < screened_molecules.sdf> \
-output < screened_molecules.best.sdf > -output_max 100 \
-sort <QSAR_Score> -reverse \
–logger File < screened_molecules.best.log>

In this example, the reverse flag indicates that the scores will be sorted from largest to smallest (default behavior is smallest to largest). Not more than 100 molecules will be output into the file screened_molecules.best.sdf.gz because of the output_max specification (the default behavior returns all molecules in the new order).

In the previous section, we demonstrated that the BCL could identify duplicate compounds at multiple levels of discrimination. One important note is that redundant molecules are excluded (i.e., sent to the output_dupes file) in the order in which they are observed in the original input. Often, the user may want to control this sequence by sorting the molecules according to some property. In these cases, molecule:Reorder can be used to do just that.

Finally, a general note on SDF input and output. Aromaticity is automatically detected when reading input files; however, output structures are Kekulized (represented as alternating single-double bonds) by default. To output an SDF that contains explicit aromatic bonds (achieved by labeling bond order as 4 in the MDL SDF), pass the explicit_aromaticity flag on the command line.

### Making Fragments

The BCL application molecule:Split gives researchers a tool to derive fragments from starting small molecules to aid in pharmacophore modeling, fragment-based drug discovery, and *de novo* drug design. There are many different types of fragments molecule:Split is able generate from whole molecule(s) (Table 2).

**TABLE 2:** Fragment splits currently supported by the BCL.

Molecule Split Implementation	Description	Customizations
Scaffolds	returns Murcko scaffolds of molecules (Bemis and Murcko, 1996)	None
Inverse Scaffold	returns the remaining components of a molecule after the Murcko scaffold is removed (Bemis and Murcko, 1996)	None
GADD Fragments	splits molecules into GA-based Drug Database fragments (Daylight Theory: SMILES)	None
Largest Common Substructure	splits molecules into their maximum common substructures relative to an input set	level of equivalence of element- and bond- type comparisons
ECFP Fragments	splits molecules into radial fingerprint fragments similar to those used for extended connectivity fingerprints (Rogers and Hahn, 2010)	bond distance from each reference atom
Linear Fragments	splits molecule into linear non-branching fingerprint fragments similar to Obabel FP2 fingerprints	bond distance from each reference atom
Rings	returns all ring components of molecules	None
Rings With Unsaturated Substituents	returns ring components of molecules along with their unsaturated substituents	None
Unbridged Aromatic Rings	returns unbridged aromatic ring components of molecules	None
Unbridged Rings	returns unbridged ring components of molecules	None
Chains	returns non-ring (chain) components of molecules	None
Rigid	splits a molecule into rigid components; defined by breaking non-ring, non-amide single-bonds to heavy atoms	None
Rigid Sans Amide	splits a molecule into rigid components; defined by breaking non-ring, non-amide single-bonds to heavy atoms	None
Isolate	splits a molecule with multiple disconnected parts (e.g., salt crystal) into component parts	None
Largest	splits a molecule with multiple disconnected parts (e.g., salt crystal) into component part and returns the largest component by molecular weight	None

For example, we can derive the Murcko scaffold from the FDA-approved 3rd generation tyrosine kinase inhibitor (TKI) osimertinib (Ramalingam et al., 2017) as follows:

bcl.exe molecule:Split \
-input_filenames osimertinib. sdf.gz \
-output osi. murcko.sdf.gz \
-implementation Scaffolds

Alternatively, we could remove the Murcko scaffold and return the other components:

bcl.exe molecule:Split \
-input_filenames osimertinib. sdf.gz \
-output osi. inverse_scaffold.sdf.gz \
-implementation InverseScaffold

Substructure comparisons are described in more detail in Section 5.1.

### Coordinate Information

The last application of interest for molecule processing is molecule: Coordinates molecule: Coordinates is a minor application that performs several convenience tasks. First, molecule: Coordinates can recenter all molecules in the input file(s) to the origin. Second, it can compute molecular centroids. Third, molecule: Coordinates can compute statistics on molecular conformers.

For example, passing the statistics flag compute statistics on bond lengths, bond angles, and dihedral angles. Passing the dihedral_scores flag will compute a per-dihedral breakdown of the BCL 3D conformer score. The BCL 3D conformer score, or ConfScore, computes an amide non-planarity penalty in addition to a normalized dihedral score. Passing the amide_deviations and amide_penalties will output the amide deviations and penalties on a per-amide basis, respectively. This can be useful when comparing conformations obtained from conformation sampling algorithms, crystal structures, and/or molecular dynamics (MD) trajectory ensembles. See Section 4 for more information on conformer sampling.

## Computing Molecular Properties

Computing molecular descriptors/properties is a critical component of cheminformatics model building. We use the term “properties” to refer to individual chemical features and “descriptors” to refer to combinations of properties, often used to train QSAR/QSPR models; however, the terms are often used interchangeably in the BCL. In conjunction with substructure-based comparisons, generating molecular descriptors is arguably the foundation of LB CADD. The BCL was designed with a modular descriptor interface and extensible property definitions framework. This allows both developers and users alike to write new descriptors for specific applications as needed. To see a list of available predefined molecular properties, perform the following command:

bcl.exe molecule:Properties–help

The property interface is organized into two general categories: 1) Descriptors of Molecules, and 2) Descriptors of Atoms. As you will see throughout this section and Section 6, properties can be modified and recombined in a highly customizable fashion. See the Supplementary Materials for an example containing multiple custom property definitions, as well as for sample output from the molecule:Properties help menu options detailing available features.

### Computing Whole Molecule Properties

As the names suggest, some descriptors are intrinsic to the whole molecule, while others are specific to atoms. For example, compute some whole molecule descriptors for the EGFR kinase inhibitor osimertinib:

bcl.exe molecule:Properties \
-input_filenames osimertinib. sdf.gz \
-output osi. mol_properties.sdf.gz \
-add Weight NRotBond NRings TopologicalPolarSurfaceArea \
-tabulate Weight NRotBond NRings TopologicalPolarSurfaceArea \
-output_table osi. mol_properties.table.txt

The flag add will add the specified properties to the SDF as MDL properties. The tabulate flag will output the properties for each molecule in row-column format in the file specified by output_table. There is also a statistics flag that will compute basic statistics for each of the specific descriptors across all the molecules in the input SDFs and output to output_histogram. The key observation regarding the output file is that the values for Weight, NRotBond, etc., are emergent properties of the whole molecule.

### Computing Atomic Properties

Next, compute some atomic descriptors for osimertinib:

bcl.exe molecule:Properties \
-add_h–neutralize \
-input_filenames osimertinib. sdf.gz \
-output osi. atom_properties.sdf.gz \
-add Weight Atom_SigmaCharge Atom_TopologicalPolarSurfaceArea \
-tabulate Atom_SigmaCharge Atom_TopologicalPolarSurfaceArea \
-output_table osi. atom_properties.table.txt \
-statistics Atom_SigmaCharge Atom_TopologicalPolarSurfaceArea \
-output_histogram osi. atom_properties.hist.txt

Notice here that the statistics flag outputs statistics across each atom property rather than across each molecule property. This is also the behavior when there are multiple input molecules. Importantly, here we see that the output is an array of values for each property. The indices of the array correspond to the atom indices of the molecule.

### Performing Operations on Descriptors

Each category of descriptors can further be modified by molecule-specific or atom-specific operations. For example, some whole molecule properties can be obtained by performing simple operations on the per-atom properties. TopologicalPolarSurfaceArea (whole molecule property) is the sum of Atom_TopologicalPolarSurfaceArea (atomic property) across the whole molecule.

bcl.exe molecule:Properties \
-add_h–neutralize \
-input_filenames osimertinib. sdf.gz \
-output osi. mol_properties.sdf.gz \
-add TopologicalPolarSurfaceArea \
“MoleculeSum (Atom_TopologicalPolarSurfaceArea)”

Check to verify that TopologicalPolarSurfaceArea and MoleculeSum (Atom_TopologicalPolarSurfaceArea) yield the same value for osimertinib.

Examples of additional operations include other basic statistics (mean, max, min, standard deviation, etc.), property radial distribution function (RDF), Coulomb force, and shape moment. See the help menu for additional options and details.

### Combining Properties to Evaluate Druglikeness

In Section 2.2 we discussed using the molecule:Filter application to remove molecules from a dataset that failed specific druglikeness criteria (e.g., TPSA ≥140 Å^2^). Several familiar druglikeness metrics come prepackaged in the BCL (i.e., Lipinski’s Rule of 5 and Veber’s Rule), as well as several others inspired by the literature and conventional medicinal chemistry practices. For each molecule in the Platinum Diverse dataset, count how many Lipinski and Veber violations there are. In addition, count as drug-like all molecules that have fewer than two Lipinski violations:

bcl.exe molecule:Properties \
-input_filenames platinum_diverse_dataset_2017_01. sdf.gz \
-output_table platinum_diverse_dataset_2017_01. druglike.txt \
-tabulate LipinskiViolations LipinskiViolationsVeber LipinskiDruglike

The property LipinskiViolations counts how many times a molecule violates one of Lipinski’s Rules ( ≤ 5 hydrogen bond donors (HBD; –NH and–OH groups), ≤10 hydrogen bond acceptors (HBA; any–N or–O), molecular weight (MW) < 500 Daltons, and water-octanol partition coefficient (logP) < 5). The LipinskiViolationsVeber property computes the number of times a molecule violates Veber’s Rule (infraction if TPSA ≥140 Å^2^ and/or number of rotatable bonds >10). The LipinskiDruglike property is a Boolean that returns 1 if fewer than two Lipinski violations occur; 0 otherwise. There is no equivalent Boolean operator for Veber druglikeness; however, it is simple to implement one using the aforementioned operators.

bcl.exe molecule:Properties \
-input_filenames platinum_diverse_dataset_2017_01. sdf.gz \
-output_table platinum_diverse_dataset_2017_01. veber_druglike.txt \
-tabulate “Define [VeberDruglike = Less (lhs = LipinskiViolationsVeber, rhs = 1)]” VeberDruglike

This command makes use of the Define and Less operators to return 1 if there are no violations to Veber’s Rule and 0 otherwise. New properties created with Define can also be passed to subsequent operators on the same line. For example, one could create a descriptor called VeberAndLipinskiDruglike by doing the following:

bcl.exe molecule:Properties \
-input_filenames platinum_diverse_dataset_2017_01. sdf.gz \
-output_table platinum_diverse_dataset_2017_01. veber_druglike.txt \
-tabulate \
“Define [VeberDruglike = Less (lhs = LipinskiViolationsVeber, rhs = 1)]” \
“Define [VeberAndLipinskiDruglike = Multiply (LipinskiDruglike, VeberDruglike)]” \
VeberAndLipinskiDruglike

This new descriptor returns 1 if a molecule passes both druglikeness filters, and 0 otherwise.

Many metrics can be created using the BCL descriptor framework without modifying the source code. This can be useful to users who come across novel methods in the literature and wish to implement them in their own work. Take as an example a seminal work from Bickerton et al., which sought to quantify the chemical aesthetics of potential druglike compounds. Bickerton et al. asked 79 medicinal chemists at AstraZeneca to answer “would you undertake chemistry on this compound if it were a hit?” for ∼200 compounds each, to which chemists replied either “yes” or “no” (Bickerton et al., 2012). They generated a regression function that yielded a quantitative estimate of druglikeness (QED) using eight chemical descriptors: molecular weight, logP, number of hydrogen bond acceptors, number of hydrogen bond donors, polar surface area, number of rotatable bonds, number of aromatic rings, and number of ALERTS (Bickerton et al., 2012).

Using the same dataset and descriptors as Bickerton et al. (generously provided in their Supplemental Materials), similar druglikeness metrics can be implemented in the BCL through the descriptor framework. One approach could be to use the operators described above to reproduce the algebraic expression described in Eq. 1 of Bickerton et al. with the parameters described in their Supplemental Materials. The algebra expressed in BCL notation can be saved to an external text file and passed to the command-line using standard shell script syntax (e.g., @File.txt in Bash). Because there are relatively few descriptors in the Bickerton et al. model, an alternative approach could be to create a classification model.

Here, we demonstrate the latter by (Eq. 1) generating a decision tree (DT) model and then 2) converting our DT into a single logic statement to pass to the BCL descriptor interface. For comparison, we also generate linear regression (LinReg) and artificial neural network (ANN) models, and we include the original QED score. All models are trained to predict a chemist’s verdict for each potential compound based on the descriptors used in Bickerton et al. (for details on model training and validation, see Supplementary Methods; for details on how to build machine learning models with the BCL, see Section 7).

**FIGURE 1:**
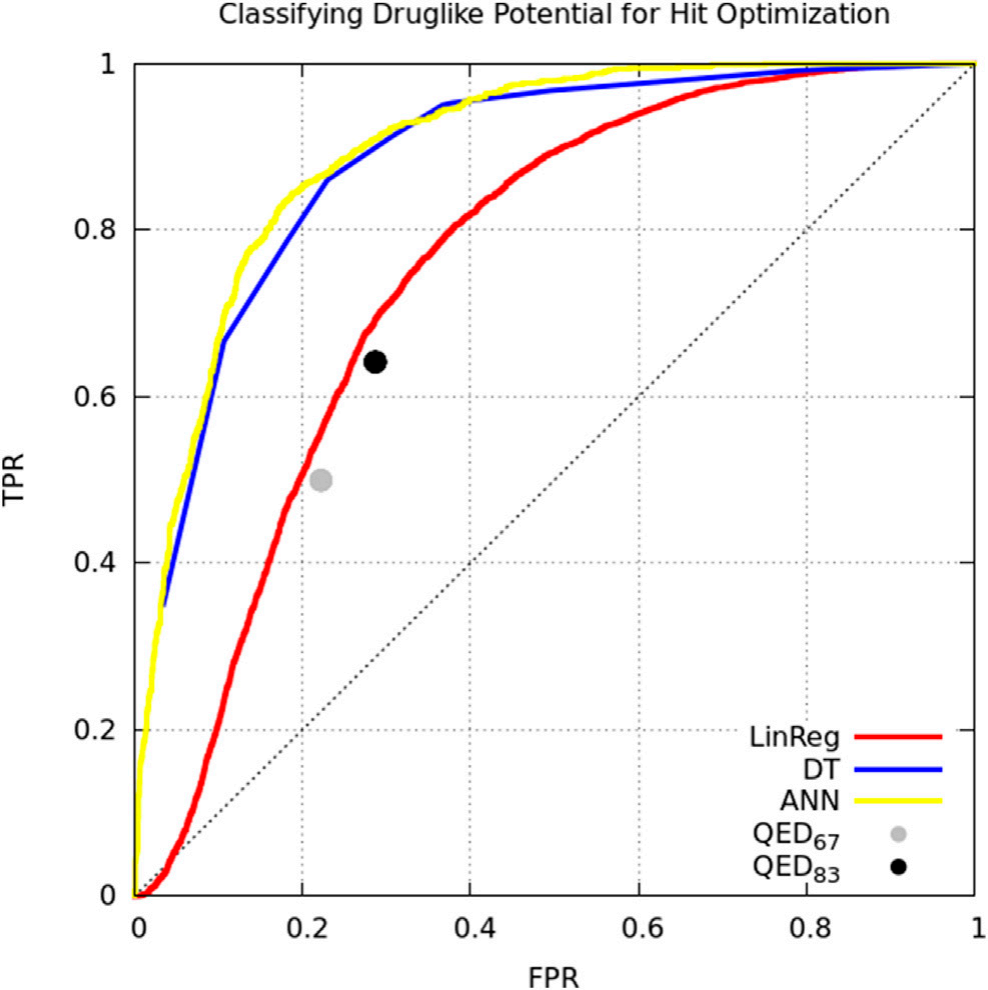
Classifying small molecules’ potential for hit optimization. Models were trained to predict whether medicinal chemists would perform hit optimization on target molecules (“yes” or “no”) starting with seven descriptors: molecular weight, logP, number of hydrogen bond acceptors, number of hydrogen bond donors, polar surface area, number of rotatable bonds, number of aromatic rings. Model types include linear regression (red), decision tree (blue), single-layer artificial neural network (yellow), and the quantitative estimate of druglikeness score with cutoffs at mean score for attractive molecules (0.67; gray) and the mean plus one standard deviation (0.83; black) by Bickerton et al. (Bickerton et al., 2012). Models trained on supplemental data from Bickerton et al. (Bickerton et al., 2012).

Model classification performance is displayed as a receiver-operating characteristic (ROC) curve (Figure 1). Bickerton et al. found that the mean QED score for molecules that medicinal chemists found favorable was 0.67 (±0.16 standard deviation). Taking the mean and mean plus standard deviation QED scores as cutoffs, we see that QED performs comparably to multiple linear regression. The ANN and DT perform better, but perhaps owing to the small number of and simple relation between variables there is no performance benefit of the ANN over the DT (Figure 1).

Now that we have our DT, we can reduce it to a readable if-else style format that can be converted into a BCL descriptor. Run the script SimplifyDecisionTree.py, passing as an argument the DT model:

/path/to/bcl/scripts/machine_learning/analysis/SimplifyDecisionTree.py \
./models/DT/model000000. model > DT. logic_summary.txt

We can see in the contents of DT. logic_summary.txt that the first thing the DT checks is whether the small molecule has less than two aromatic rings. Molecules with no aromatic rings are excluded, and molecules with one aromatic ring are subject to different criteria than molecules with two or more. Subsequent criteria are then evaluated. We can rewrite the logic summary as a descriptor and save it in a file called “dt.obj”. Then, we pass that file to molecule:Properties as a descriptor definition and use it to classify molecules:

bcl.exe molecule:Properties \
-add_h -neutralize \
-input_filenames platinum_diverse_dataset_2017_01. sdf.gz \
-output_table platinum_diverse_dataset_2017_01. dt_druglike.txt \
-tabulate “Define (Hitlike = @dt.obj)” Hitlike

The “dt.obj” code object file is a plain text file that can be opened with any text editor. The syntax mimics the BCL command-line syntax. Code object files are a convenient way to write a long, multi-line BCL command-line that makes it easier to build and reuse feature sets.

On the topic of druglikeness, it is worth noting that additional advanced methods are also available to classify the chemical space of molecules in a dataset. In some cases, it is useful to identify potential drug-like compounds that not only fit the criteria discussed above but are also similar to some known class (es) of drugs. For example, when performing fragment-based combinatorial library design for kinase inhibitors, in addition to filtering out molecules that violate Veber’s rules, it may also be desirable to filter molecules that are not sufficiently chemically similar to existing kinase inhibitors. This can be accomplished by building and scoring against an applicability domain (AD) model. For further details on creating and using AD models in the BCL, see Section 7.4.3.

We have described multiple uses of the molecule:Properties application, placing special emphasis on how it can be utilized to build different types of druglikeness metrics. As it is fundamentally a tool to obtain information from small molecule chemical structures, molecule:Properties can also be used to help generate statistical potentials, chemical filters, QSAR/QSPR models, and more. Some of these use-cases will be explored in later sections.

## Small Molecule Conformer Generation

Small molecule 3D conformer generation is a critical aspect of both SB and LB CADD because the biologically relevant conformation of the molecule of interest is rarely known *a priori*. In SB molecular docking, small molecule flexibility is often represented through the inclusion of multiple discrete pre-generated conformers (Brylinski and Skolnick, 2008; Morris et al., 2009; Lemmon and Meiler, 2012; Combs et al., 2013; DeLuca et al., 2015). Small molecule conformations need to be sampled to arrive at the correct binding pose. Molecules that appear in binding pockets of substantially different proteins often bind in distinct modes for each protein, suggesting that the binding pose of the molecule need not be near the global energy minima of the molecule (Nicklaus et al., 1995; Boström et al., 1998; Perola and Charifson, 2004; Sitzmann et al., 2012; Friedrich et al., 2018). Likewise, in LB pharmacophore modeling, small molecules need to be flexibly aligned according to their chemical properties to identify the biologically relevant 3D features conferring bioactivity.

The BCL conformer generator, also called BCL:Conf, utilizes a fragment-based rotamer library derived from the crystallography open database (COD) to combine rotamers consisting of one or more dihedral angles according to a statistically-derived energy (Mendenhall et al., 2020). Clashes are dynamically resolved by iteratively identifying clashed atom pairs and rotating the central-most bonds between them without changing dihedral bins. In this way, conformational ensembles are stochastically generated according to likely rotamer combinations from the COD.

The BCL small molecule conformation sampler is a leader among general purpose small molecule conformer generation algorithms (Kothiwale et al., 2015; Mendenhall et al., 2020). In this section, we demonstrate how to use the BCL to generate global and local conformational ensembles and sample discrete rotamers within a molecule.

### Generating Global Conformational Ensembles

Start by generating conformers of osimertinib with the default settings. Here, all that is needed is an input filename and an output filename:

bcl.exe molecule:ConformerGenerator \
-ensemble_filenames osimertinib. sdf.gz \
-conformers_single_file osimertinib. global_confs.sdf.gz

The ensemble_filenames argument is equivalent to the input_filenames argument used elsewhere (the difference is historical). The conformers_single_file argument is one of two output options. The other option is conformers_separate_files. As implied by the name, in the former case all conformers are output to a single file. In the latter case, if multiple molecules are input to ensemble_filenames, then a unique SDF will be written for the conformational ensembles of each of the input molecules [e.g., if the input SDF(s) contained 10 molecules, then conformers_separate_files would output 10SDFs each with a conformational ensemble of one of the input molecules].

By default, BCL:Conf will perform 8,000 conformer generation iterations, each of which rebuilds the molecule essentially from scratch (excepting rigid ring structures and bonds that do not vary substantially in length or angle). Without any other options, the top conformations will be clustered, yielding the 100 best-scoring representatives of each different cluster. An unbiased view of the conformational space around the ligand can be obtained by setting the skip_cluster flag. For this application, it is advisable to lower the number of iterations to roughly double the number of desired conformations; the conformers are rebuilt from scratch at every iteration, so there is little gain from doing more iterations than conformers desired. The returned conformers are sorted by score. Number of iterations and final conformers can be specified with the max_iterations and top_models flags, respectively.

Conformations can be filtered to remove highly-similar conformations using the conformation_comparer flag (e.g., to standard RMSD, dihedral distance, etc.) and the tolerance for what constitutes an “identical” conformer increased from the default (0.0) to an arbitrarily large value (note that RMSD- and dihedral-based metrics have units of Å and degrees, respectively) (Kothiwale et al., 2015). For most applications, we recommend the use of SymmetryRMSD with a modest tolerance of 0.25 Å. By default, the tolerance is adjusted automatically to yield the desired number of clusters so as to best represent conformational space, however, a user-provided tolerance is treated as a minimal acceptable difference between clusters.

For high-throughput applications, we recommend reducing iterations from 8,000 down to 800 or even 250. BCL:Conf’s speed is nearly linear in number of iterations. Generally, more iterations yield better performance, at a trade-off of slightly-faster than linear increase in time per conformation when clustering is used (Mendenhall et al., 2020).

Alternatively, if conformation_comparer is set to “RMSD 0.0”, then no filtering or clustering is specified, and BCL:Conf will perform max_iterations conformer generation iterations, randomly select top_models conformers, sort them from best to worst by score, and return them. This option is the fastest, and the ensembles returned are arguably the most Boltzmann-like. For a recent comparison of each set of parameters to one another and other conformer generation algorithms, please see Mendenhall et al., 2020 (Mendenhall et al., 2020). We recommend generating conformers with explicit hydrogen atoms added.

Generate conformers using two of the protocols described protocols. First, run

bcl.exe molecule:ConformerGenerator \
-add_h -ensemble_filenames osimertinib. sdf.gz \
-conformers_single_file osimertinib. symrmsd_cluster.confs.sdf.gz \
-max_iterations 8,000 –top_models 25 \
-conformation_comparer SymmetryRMSD 0.25

Then,

bcl.exe molecule:ConformerGenerator \
-add_h -ensemble_filenames osimertinib. sdf.gz \
-conformers_single_file osimertinib. raw.confs.sdf.gz \
-max_iterations 8,000 –top_models 250 –skip_cluster
-conformation_comparer RMSD 0.0

Notice that the ensemble generated with the SymmetryRMSD comparer and clustering enabled occupies the densest part of the broader conformational space sampled in the raw distribution.

### Generating Local Conformational Ensembles

Local sampling was implemented in the recent algorithmic improvements to BCL:Conf (Mendenhall et al., 2020). The idea is that sometimes users know or have predicted with some degree of certainty a chemically meaningful or bioactive pose of a small molecule, but additional refinement is needed. This is a common use case when modeling protein-ligand complexes starting with another ligand with some similarity to the ligand of interest (Bozhanova et al., 2021; Hanker et al., 2021). When using pre-generated conformers for docking or small molecule flexible alignment, it is unlikely that the best ligand conformer will be chosen and simultaneously have its position fully optimized in Cartesian space. Local sampling around an input conformer allows the user to refine ligand poses after an initial search.

Local sampling in the BCL is accomplished by restricting the rotamer search in one of four ways:

1. -skip_rotamer_dihedral_sampling–preserve input dihedrals to within 15-degrees of closest 30-degree bin (centered on 0°) in non-ring bonds.
2. -skip_bond_angle_sampling–preserve input conformer bond lengths and angles
3. -skip_ring_sampling–preserve input ring conformations
4. –change_chirality–by default, input chirality and isometry are preserved. Use this flag to allow for generation of enantiomers and stereoisomers.

These options are not mutually exclusive. Depending on how they are combined, different levels of sampling can be achieved. Moreover, they can be used in combination with any of the other options (e.g., conformation comparison type, clustering) described above. Generate local conformational ensembles of osimertinib by first placing all three restrictions:

bcl.exe molecule:ConformerGenerator \
-ensemble_filenames osimertinib. sdf.gz \
-conformers_single_file osimertinib. skip_all.local_confs.sdf.gz \
-skip_rotamer_dihedral_sampling -skip_bond_angle_sampling \
-skip_ring_sampling–skip_cluster

Next, apply only the skip_rotamer_dihedral_sampling and skip_bond_angle_sampling restrictions to generate a local ensemble:

bcl.exe molecule:ConformerGenerator \
-ensemble_filenames osimertinib. sdf.gz \
-conformers_single_file osimertinib. skip_dihed_ring.local_confs.sdf.gz \
-skip_rotamer_dihedral_sampling -skip_ring_sampling–skip_cluster

Both of the ensembles show less conformational diversity than the global conformational ensemble created in the previous section. Notice the relative sampling differences between each of the local conformation sampling protocols described.

### Conformational Sampling of Substructures

Often times one may wish to only sample conformations of part of a molecule. For example, in docking congeneric ligand series, the core scaffold pose may be known with a high degree of confidence, and the goal is to optimize the pose of the rest of the molecule while keep the core scaffold fixed. Alternatively, crystal structures of protein-ligand complexes often have low or missing density for part of a bound ligand, and thus coordinate assignment may not accurate. Discretely sampling specific small molecule rotamers thus becomes a useful task to perform.

In the BCL, this is accomplished by first assigning an MDL miscellaneous property named “SampleByParts” to the molecule(s) of interest. The value of the SampleByParts property corresponds to the 0-indexed atom indices of atoms in dihedrals that are allowed to be sampled by molecule:ConformerGenerator. By encoding this as a molecule-specific property, we avoid multiple command-line calls with different atom index specifications, allowing users to generate conformers more rapidly for multiple molecules and/or different independent rotamers within a molecule.

As an example, consider a crystal structure of epidermal growth factor receptor (EGFR) kinase in complex with osimertinib (PDB ID 4ZAU) (Yosaatmadja et al., 2015). This is the first publicly available crystal structure of the EGFR-osimertinib complex. In this structure, the solvent-exposed ethyldimethylamine substituent is missing density. We will sample alternative conformations of the ethyldimethylamine substituent than that which is proposed in the PDB ID 4ZAU. First, add the corresponding atom indices to the file osimertinib. sdf:

bcl.exe molecule:Properties \
-add “Define [SampleByParts = Constant (3,36,18,19,6,20,21)]” SampleByParts \
-input_filenames osimertinib. sdf.gz–output \
osimertinib.sample_by_parts.sdf

Also, note that if you have many molecules for which you want to assign SampleByParts atom indices and you do not want to have to manually identify the relevant indices, you can also use the molecule:SetSampleByPartsAtoms application. This application sets SampleByParts indices based on comparison to user-supplied substructures. With the SampleByParts property defined in the SDF, generate global conformers as previously described:

bcl.exe molecule:ConformerGenerator \
-ensemble_filenames osimertinib. sample_by_parts.sdf.gz \
-conformers_single_file osimertinib. sample_by_parts.confs.sdf.gz \
-top_models 250 –cluster

**FIGURE 2:**
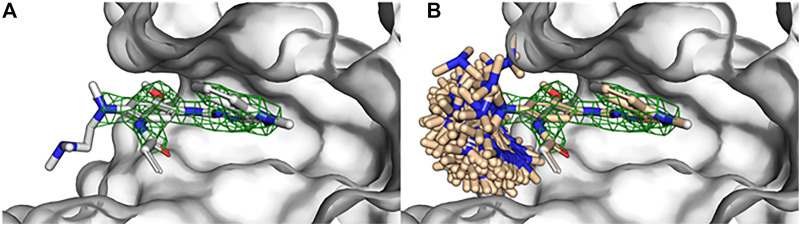
Substructure sampling of small molecule rotamers with BCL:Conf. **(A)** Crystallographic structure of osimertinib bound to EGFR kinase (PDB ID 4ZAU) contains missing density of the ethyldimethylamine substituent of osimertinib. **(B)** Global conformational sampling of the osimertinib ethyldimethylamine substituent without perturbing the rest of the bound pose using BCL:Conf. Osimertinib electron density visualized with green mesh by importing the 2fo-fc map in PyMOL and contouring at 2σ.

Observe that sampling global conformers (i.e., sampling across dihedral bins allowing bond angle/length adjustment and ring conformer changes) with SampleByParts maintains the coordinates of all unspecified atoms. In this case, only dihedrals containing strictly the ethyldimethylamine atoms are sampled (Figure 2). Similarly, SampleByParts can be used in conjunction with the local sampling methods described above.

## Molecule Property- and Substructure-based Comparisons

A critical component of LB CADD is molecular similarity analysis. Provided a set of molecules, we frequently want to know how similar each molecule is to a reference molecule(s). Fundamentally, this requires 1) defining what specifically will be compared between the molecules, and 2) defining the metric with which similarity will be measured. In the BCL, this is accomplished primarily through use of the molecule:Compare application. The command-line syntax of molecule:Compare differs from the syntax of other applications discussed so far. The SDF input files to molecule:Compare are passed as parameters instead of argument flags.

bcl.exe molecule:Compare < mandatory_parameter_one.sdf> \
<optional_parameter_two.sdf> –output < mandatory_output.file> \

This syntax strictly enforces two types of behavior:

1. If a single SDF is specified as a parameter, then all molecules in the file are compared with one another
2. If two SDFs are specified, then the molecule(s) in the second file will be compared against the molecule(s) in the first file.

Finally, it is worth noting that molecule:Compare’s performance scales approximately linearly with number of threads for costly comparisons. To enable threads, set -scheduler PThread <number_threads>. We suggest setting number_threads to number of physical cores on the device for maximum performance.

### Defining Molecular Structures

The BCL encodes molecules as graphs where the edges are bonds, and the atoms are nodes. For substructure-based comparisons, we can define equivalence between bonds and atoms using various comparisons dubbed comparison types. For any substructure-based comparison between two or more molecules, some combination of atom and bond comparison types is required, which defines the equivalence class for the atoms and bonds, respectively. The default combination differs between tasks. For a summary of available atom and bond type comparisons, examine the help menu options of any comparer that utilizes substructures. For example,

bcl.exe molecule:Compare \
-method “LargestCommonSubstructureTanimoto (help)”

will display the default atom and bond comparison types for this comparison method as well as list the available comparison types. For example, if atom type resolution occurs at AtomType, then an SP3 carbon would match another SP3 carbon but not an SP2. If the resolution is lowered to ElementType, then all carbon atoms can match one another independent of their orbital configuration. Similarly, bond type resolutions of BondOrder and BondOrderAmideWithIsometryOrAromaticWithRingness will yield different substructure matches.

Not all similarity comparisons occur at the structural/substructural level. A number of comparison metrics in the BCL occur between properties computed at the whole molecular, substructural, or atomic level. Further, distance-based comparisons between molecules that are constitutionally identical can also be made.

### Similarity Scoring Between Constitutionally Unique Molecules

In cases where the similarity between unique molecules is desired there are broadly two approaches for measuring similarity: by substructure and by property. These are not mutually exclusive; depending on the desired resolution of the substructure comparisons, one can further measure property differences between substructures of different molecules.

One common substructure similarity metric is the Tanimoto coefficient (TC), expressed between two molecules as the ratio of matched-to-unmatched atoms:

$$TC=\frac{\left|A\cap B\right|}{\left|A\right|+\left|B\right|-\left|A\cap B\right|},$$ (1)

where A and B are the two molecules. The intersection of atoms in (Eq. 1) is the size of the largest common substructure under the specified comparison types. This is a specific formalism of the more general Tversky index when both α and β are equal to 1:

$$TC=\frac{\left|A\cap B\right|}{\left|A\cap B\right|+\alpha \left|A-B\right|+\beta \left|B-A\right|},$$ (2)

**FIGURE 3:**
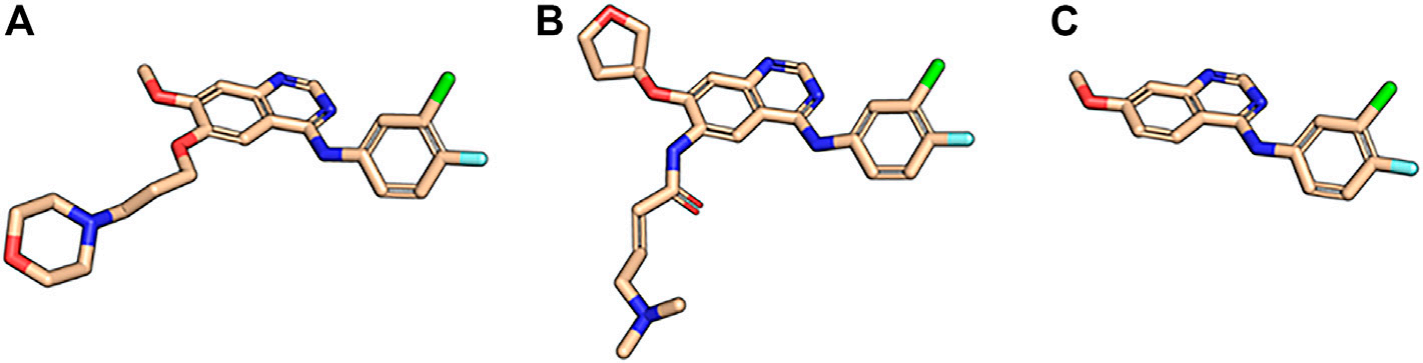
Maximum common substructure between gefitinib and afatinib. **(A)** Afatinib (PDB ID 4G5J) and **(B)** gefitinib (PDB ID 4I22) in their binding mode 3D conformations next to **(C)** their maximum common substructure extracted with the BCL.

The first-generation EGFR tyrosine kinase inhibitor gefitinib and the second-generation inhibitor afatinib are structurally very similar. Afatinib is modified from the gefitinib scaffold and incorporates an acrylamide linker. Visualize the maximum common substructure (MCS) of afatinib and gefitinib using molecule:Split (Figure 3):

bcl.exe molecule:Split \
-implementation “LargestCommonSubstructure (file = afatinib.sdf)" \
-input_filenames gefitinib. sdf.gz–output mcs_gef_afa.sdf.gz

Next, calculate the MCS TC of the gefitinib and afatinib:

bcl.exe molecule:Compare gefitinib. sdf.gz afatinib. sdf.gz \
-method LargestCommonSubstructureTanimoto–output gef_afa_mcs_tani.txt

This method searches for the single largest common connected substructure as the intersection of two molecules and computes the TC. In this case, the MCS TC is approximately 0.48. Sometimes searching for a single connected substructure can be disadvantageous. For example, if the primary differences between molecules results from core substitutions bridging two otherwise identical halves, then the single largest common substructure approach will fail to account for the complete degree of similarity. Alternatively, the user can calculate the maximum common disconnected substructure (MCDS) TC:

bcl.exe molecule:Compare gefitinib. sdf.gz afatinib. sdf.gz \
-method LargestCommonDisconnectedSubstructureTanimoto \
–output gef_afa_mcds_tani.txt

As expected, the MCDS TC is greater than the MCS TC at approximately 0.86.

### Distance-Based Scoring Between Constitutionally Identical Molecules

In Section 4 we demonstrated how the BCL can be used to generate small molecule conformational ensembles. One common way to measure the performance of small molecule conformer generators is to measure how close we can recover biologically relevant conformations. We can do this in the BCL by measuring the RMSD or SymmetryRMSD of molecules in our conformational ensemble to the experimentally determined conformations. Generate a global ensemble of osimertinib:

bcl.exe molecule:ConformerGenerator \
-add_h -ensemble_filenames osimertinib. sdf.gz \
-conformers_single_file osimertinib. confs.sdf.gz \
-max_iterations 8,000 –top_models 50 –cluster \
-conformation_comparer SymmetryRMSD 0.25 –generate_3D

Note that we are generating the molecule completely *de novo* ignoring all information from input coordinates by using generate_3D. Measure the heavy-atom symmetric RMSD to the native conformation:

bcl.exe molecule:Compare osimertinib. sdf.gz osimertinib. confs.sdf.gz \
-method SymmetryRMSD -logger File osi. sym_rmsd_native.log \
-output osi. sym_rmsd_native.txt -remove_h

On examination of osi. sym_rmsd_native.txt, we see that see that of our 25 generated conformers, 3 are less than 2.0 Å from the native conformer, and the best is approximately 0.66 Å from native. If we repeat this process for two additional TKIs, the first-generation inhibitor erlotinib and the second-generation inhibitor afatinib, we also see that we are able to obtain multiple conformers less than 1.0 Å from native.

In addition to RMSD-based metrics, molecule:Compare can also measure distance in the form of dihedral angle sums and dihedral distance bins. For additional information, examine the help menu options.

### Largest Common Substructure Alignment

The BCL can be used to align small molecules according to their MCS. Unlike most of the examples in this section, this is accomplished through the molecule:AlignToScaffold application by passing three parameters:

bcl.exe molecule:AlignToScaffold <scaffold> <ensemble> <output>

For example, to align afatinib to gefitinib based on their MCS, use the following command:

bcl.exe molecule:AlignToScaffold gefitinib. sdf.gz afatinib. sdf.gz \
afatinib.ats.sdf.gz \

Instead of aligning by MCS, the user may also align the target ensemble to the largest rigid component of the scaffold structure by passing the align_rigid flag. Moreover, if the user wants to a define an alternative set of atoms to be aligned instead of the defaults, this can be accomplished by specifying those atoms for each the scaffold and target ensemble with align_scaffold_atoms and align_ensemble_atoms, respectively.

### Property-Based Flexible Alignment

In addition to substructure-based alignment, we can also perform property-based alignment. Property-based alignment algorithms typically maximize the overlap or minimize the distance between molecular and/or atomic properties (Sliwoski et al., 2014). We have previously demonstrated that the performance of the BCL property-based alignment algorithm, also referred to as BCL:MolAlign, is on par with leading academic and commercial molecular alignment algorithms (Brown et al., 2019).

BCL:MolAlign combines the conformational sampling ability of BCL:Conf with the property framework described in Section 3 to minimize the property-distance between two molecules through flexible superimposition. The property-distance is computed between mutually-matching atom pairs that are dynamically updated with each iteration. Alignment pose sampling is accomplished through a series of moves that traverse the co-space defined by the relative position of the two molecules to one another (Brown et al., 2019). BCL:MolAlign can be used to perform alignments which can be classified as rigid (two molecules with fixed conformers), semi-flexible (one molecule with a fixed conformer, one molecule whose conformers are sampled), and fully-flexible (two molecules whose conformers are sampled).

**FIGURE 4:**
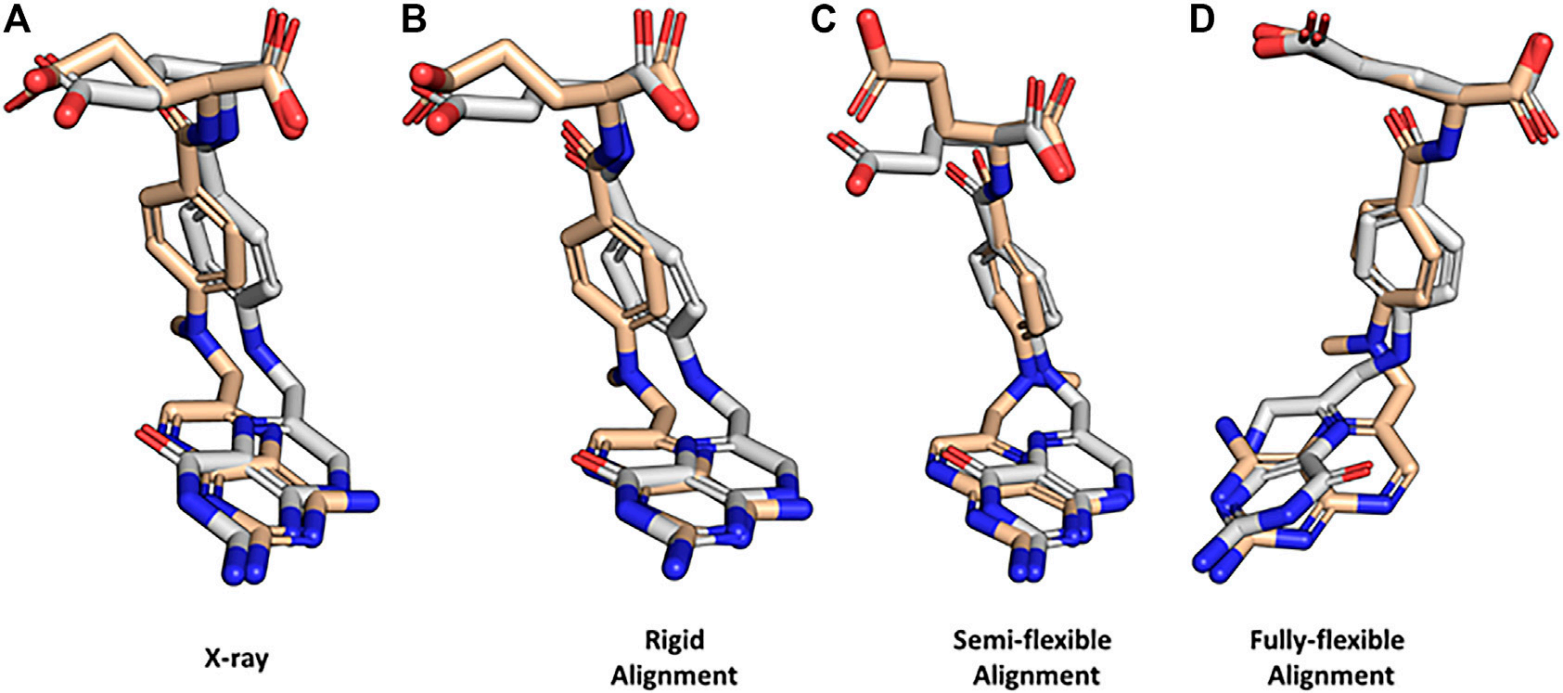
Property-based alignment of dihydrofolic acid and methotrexate with BCL:MolAlign. **(A)** Superimposed crystallographic structures of dihydrofolic acid (DHF; PDB ID 1DHF) and methotrexate (MTX; PDB ID 1DLS) in complex with dihydrofolate reductase (DHFR). **(B)** Rigid alignment of DHF and MTX starting from the bioactive conformers from the crystal structures. **(C)** Flexible alignment of MTX (flexible) to DHF (rigid, bioactive conformer). **(D)** Fully flexible alignment of DHF and MTX. DHF is colored white and MTX is colored wheat. MTX was randomly rotated and translated prior to rigid alignment to DHF. All flexible alignments performed using generate_3D to remove bias from start coordinates.

To demonstrate how BCL:MolAlign can be used to perform each of these alignments, consider the classic problem of obtaining the crystallographic alignment of methotrexate (MTX) and dihydrofolic acid (DHF). This example is a good one because the intuitive heterocyclic overlap is not the correct one (Labute et al., 2001). Instead, alignment of the binding pockets of dihydrofolate reductase (DHFR) co-crystallized with MTX (PDB ID 1DLS) and DHF (PDB ID 1DHF) shows only partial heterocycle overlap and superimposition of the heterocycle carbonyl in DHF and an aromatic hydrogen bond accepting nitrogen in MTX (Figure 4A). Perform a rigid alignment of MTX to DHF with the following command:

bcl.exe molecule:Compare mtx. perturbed.sdf.gz dhf. sdf.gz \
-add_h–neutralize \
-output mtx_dhf_rigid_rmsdx.output \
-logger File rigid_alignment.log -random_seed \
-method “PsiField \
(
output aligned mol a = mtx. rigid_aligned.sdf,
iterations = 1,000,
number outputs = 1
)"

The rigid alignment ranks the correct alignment mode as the top scoring alignment (Figure 4B). Rigid alignments are rarely useful for drug discovery because the bioactive conformation of the target small molecule is usually unknown; however, they provide a useful check for alignment scoring functions. Next, flexibly align MTX to the DHFR-binding pose of DHF:

bcl.exe molecule:Compare mtx. perturbed.sdf.gz dhf. sdf.gz \
-add_h–neutralize \
-output mtx_dhf_rigid_rmsdx.output \
-logger File semi-flexible_alignment.log -random_seed \
-method “PsiFlexField
(
output_aligned_mol_a = mtx. semiflex_aligned.sdf,
rigid_mol_b = true,
number_flexible_trajectories = 3,
fraction_filtered_initially = 0.25,
fraction_filtered_iteratively = 0.50,
iterations = 400,
filter_iterations = 200,
refinement_iterations = 50,
conformer_pairs = 500,
number_outputs = 1,
sample_conformers = SampleConformations (
conformation_comparer = SymmetryRMSD,
generate_3D = 1,tolerance = 0.10,rotamer_library = cod,
max_iterations = 8,000,max_conformations = 50,
cluster = true)
)”

Here, we can see that BCL:MolAlign correctly determines the alignment of the heterocycles, central aromatic rings, and (partially) the acidic groups (Figure 4C). Note that rigid_mol_b is enabled, which fixes the pose of the second parameter molecule. For a detailed description of how each argument modifies the alignment algorithm, see Brown et al*.* (Brown et al., 2019). For performance considerations, we generally find that the number of conformer pairs is more critical to pose recovery than the numbers of iterations at each stage. For complex ligands with many rotational bonds, we recommend increasing max_conformations and conformer_pairs.

Fully-flexible alignment is useful when one is trying to recover pharmacophore features without knowing the binding pose of either molecule. Here, the goal is to align pharmacophore features of the molecules, not recover the native pose of the target molecule(s) by aligning to another molecule with a known binding mode. Perform a fully-flexible alignment of MTX and DHF.

bcl.exe molecule:Compare mtx. perturbed.sdf.gz dhf. perturbed.sdf.gz \
-add_h–neutralize \
-output mtx_dhf_rigid_rmsdx.output \
-logger File fully-flexible_alignment.log \
-random_seed–scheduler PThread 8 \
-method “PsiFlexField \
( \
output_aligned_mol_a = mtx-dhf. fullflex_aligned.sdf, \
rigid_mol_b = false, \
number_flexible_trajectories = 5, \
fraction_filtered_initially = 0.25, \
fraction_filtered_iteratively = 0.50, \
iterations = 800, \
filter_iterations = 400, \
refinement_iterations = 100, \
conformer_pairs = 2,500, \
number_outputs = 1, \
sample_conformers = SampleConformations ( \
conformation_comparer = SymmetryRMSD, \
generate_3D = 1,tolerance = 0.10,rotamer_library = cod, \
max_iterations = 8,000,max_conformations = 50, \
cluster = true) \
)”

Fully-flexible alignment of MTX and DHF does not recover the most native-like conformations of MTX and DHF; however, it does recover correct alignments of the heterocycles, central aromatic rings, and acidic groups (Figure 4D). Notice that we increased the number of conformer pairs from 500 to 2,500 when we went from semi-flexible to fully-flexible alignment.

## Feature Generation

The descriptor application group is the workhorse for molecule featurization. Similar to the molecule:Properties application, the descriptor application group provides command-line access to the internal descriptor framework. Unlike molecule, descriptor is dataset centric; its primary purpose is to generate, manipulate, and analyze feature datasets for QSAR/QSPR. In this section, we will demonstrate core applications in descriptor and how they can be utilized in QSAR/QSPR modeling.

### Generating Simple Datasets From Molecules

Four specifications are required to generate feature datasets from small molecules:

1. The molecules for which to generate the features; these can be any valid SDF.
2. The types of features to generate; these are properties such as those described in Section 3. Typically, these are stored in a separate file and passed to the command-line at run-time; however, they can also be specified directly on the command-line. Importantly, combining multiple descriptors for feature generation requires the use of the Combine descriptor.
3. The feature result label; this indicates the output(s) that models will train toward. This can be a constant value (i.e., if featurization is being done for some purpose other than model training), a property (e.g., LogP for a QSPR model), or another label (e.g., bioactivity label from experimental data).
4. The output filename; three output types are available. The BCL has a partial binary format with the “.bin” suffix that is used for all model training. Feature datasets can also be output with the “.csv” suffix for a comma-separated values (CSV) file. Moreover, “.csv” files and “.bin” files can be interconverted. In this way, features generated with the BCL can be used by other software, and vice versa. For inter-operability with Weka software, “.arff” format is also supported, with a limitation of only working with continuous variables.

Generate a simple feature dataset consisting of several scalar descriptors for a set of confirmed active M1 Muscarinic Receptor positive allosteric modulators (PAMs) and corresponding true negatives (Butkiewicz et al., 2013). The SDF corresponding to these compounds is 1798. combined.sdf. These molecules have been labeled with the MDL property “IsActive” such that the confirmed actives have a value of 1 and the negatives have a value of 0.

bcl.exe descriptor:GenerateDataset \
-source “SdfFile (filename = 1798. combined.sdf)” –id_labels “String (M1)” \
-result_labels “Combine (IsActive)” \
-feature_labels “Combine (Weight, LogP,HbondDonor, HbondAcceptor)” \
-output 1798. combined.scalars.bin

Binary files were designed for rapid non-consecutive reading and writing, but the interested reader will find that the file format consists of a textual header specifying the properties and their sizes followed by a simple binary output of all features. Dataset information and statistics can be obtained by calling descriptor:GenerateDataset compare. For example:

bcl.exe descriptor:GenerateDataset–compare 1798. combined.scalars.bin

To better understand the binary file encodings, convert 1798. combined.scalars.bin to a CSV file:

bcl.exe descriptor:GenerateDataset \
-source “Subset (filename = 1798. combined.scalars.bin)” \
-output 1798. combined.scalars.csv

The first column of every row contains the ID label “M1” as specified when the binary file was generated. The next four columns contain the descriptors specified above: Weight, LogP, HbondDonor, and HbondAcceptor. The very last column is the result value, which contains either 0 or 1 depending on the value in the SDF MDL property “IsActive”.

Convert CSV file back to a binary file:

bcl.exe descriptor:GenerateDataset \
-source “Csv(filename = 1798. combined.scalars.csv, number result cols = 1, number id chars = 2)” \
-output 1798. combined.scalars.bin

CSV files do not contain all of the supplementary information contained within the partial binary file format. Thus, certain information needs to be provided directly. For example, we need to specify the number of characters that are part of the row ID label, otherwise the BCL will try to convert the string (or numerical) ID into feature values. ID labels therefore must be fixed-width. In addition, we need to tell the BCL how many of the columns are result values. By default, the BCL will assume that only the last column is the result label. By specifying number result cols = N, we tell the BCL to take the last N columns of the CSV as the result value(s).

Also notice that the feature and result label information is not informative after converting from CSV to binary. The values are transferred to the new file format, but the BCL obviously cannot know where those values came from. These must be manually specified.

bcl.exe descriptor:GenerateDataset \
-source “Csv(filename = 1798. combined.scalars.csv, number result cols = 1, number id chars = 2)” \
–id_labels “String (M1)” \
-result_labels “Combine (IsActive)” \
-feature_labels “Combine (Weight, LogP,HbondDonor, HbondAcceptor)” \
-output 1798. combined.scalars.bin

In this case, the feature labels are internal parsable properties of the BCL; however, when relabeling feature labels upon converting from CSV to binary format, the user can specify any labels so long as the total number of labels is consistent with the number of feature columns.

### Modifying Datasets

After generating a dataset or importing a CSV file and converting it to binary format, feature datasets can be modified. The most frequent form of modification is randomization. Training a machine learning model, for example a neural network, often requires dataset randomization.

bcl.exe descriptor:GenerateDataset \
-source “Randomize [Subset (filename = 1798. combined.scalars.bin)]” \
-output 1798. combined.scalars.rand.bin

Binary files are read by the “Subset” retriever. The Randomize operator is passed through the source flag and provided the dataset retriever option corresponding to the binary file.

Additional dataset operators can be classified by how they modify the dataset. For example, the PCA (principal components analysis) and EncodeByModel operators perform dimensionality reduction across feature (column) space, while the KMeans operator reduces dimensionality across molecule (row) space. Other operators are useful during model training and validation, such as Balanced, Chunks, and YScramble. Still others can be used to select particular ranges of rows from a dataset, such as Rows. Here, we will take a look at a few dataset operators. For full details on all available dataset operators, see the descriptor:GenerateDataset help menu.

Start by generating a dataset for the Kir2.1 inward rectifying potassium channel using the dataset compiled in Butkiewicz et al. (Butkiewicz et al., 2013) and the best performing LB descriptor set from Mendenhall and Meiler (Mendenhall and Meiler, 2016). This dataset contains 301,493 small molecules, 172 of which are confirmed active molecules. For each molecule, there will be 1,315 feature columns and 1 result column.

bcl.exe descriptor:GenerateDataset \
-source “SdfFile (filename = 1843. combined.sdf.gz)” –scheduler PThread 8 \
-feature_labels MendenhallMeiler2015. Minimal.object \
-result_labels “Combine (IsActive)” \
–output 1843. Minimal.bin–logger File 1843. Minimal.log

Randomize the dataset:

bcl.exe descriptor:GenerateDataset \
-source “Randomize (Subset (filename = 1843. combined.bin))” \
-output 1843. combined.rand.bin–logger File 1843. Minimal.rand.log

Note that we could have generated a randomized dataset with a single command by wrapping the SdfFile dataset retriever with Randomize; however, the Randomize dataset retriever is unable to support hyperthreading. Consequently, it is faster to generate larger datasets first using multiple threads and randomize them afterward. Next, perform PCA on the dataset using OpenCL to accelerate the calculation with a GPU. The flag opencl is optional and may not be supported on all platforms, but may provide a substantial speedup, depending on the GPU and dataset size:

bcl.exe descriptor:GeneratePCAEigenVectors \
-training “Subset (filename = 1843. Minimal.rand.bin)” \
-output_filename 1843. Minimal.PCs.dat–opencl \
-logger File 1843. Minimal.PCs.log

Finally, generate a new feature dataset accounting for 95% of the variance:

bcl.exe descriptor:GenerateDataset \
-source “PCA(dataset = Subset (filename = 1843. Minimal.rand.bin), fraction = 0.95, filename = 1843. Minimal.PCs.dat)” \
-output 1843. Minimal.rand.pca_095. bin–opencl \
-logger File 1843. Minimal.rand.pca_095. log

Performing PCA on the dataset has reduced the number of descriptors from 1,315 to 695. Alternatively, one could use EncodeByModel to reduce the number of feature columns using a pre-generated model. The following example utilizes pseudocode and a hypothetical pre-generated ANN with the MendenhallMeiler2015. Minimal.object features.

bcl.exe descriptor:GenerateDataset/
-source “EncodeByModel [storage = File (directory = /path/to/model/directory, prefix = model),retriever = Subset (filename=<my_binary_file.bin>)]” \
-output < my_encoded_binary_file.bin>

The input file < my_binary_file.bin > would have 1,315 descriptors from MendenhallMeiler2015. Minimal.object, and the output file < my_encoded_binary_file.bin > would have a number of descriptors corresponding to the number of neurons in the final hidden layer preceding the output layer of our hypothetical pre-generated ANN.

As a practical note, we have found that PCA-based dimensionality reduction useful for dataset visualization, but of limited value in improving model performance. Performance can often be recovered to that of the initial dataset when requiring at least 95% of the variance to be preserved, but performance improvement is rare from PCA, when using a regularized method such as dropout-ANNs.

Suppose you encoded the same original feature set using two different models and now want to combine the new encoded files for further training. This can readily be accomplished with the Combine operator.

bcl.exe descriptor:GenerateDataset \
-source “Combined [Subset (filename=<my_binary_file_1. bin>), Subset (filename=<my_binary_file_2. bin>)]” \
-output < my_combined_binary_file.bin>

Next, instead of performing dimensionality reduction along the column (features) axis, we will reduce the dimensionality along the row (molecule) axis. Perform K-means clustering of the feature dataset to reduce our row number from 301,493 to 300.

bcl.exe descriptor:GenerateDataset \
-source “KMeans [dataset = Subset (filename = 1843. combined.rand.bin), clusters = 300]” \
-output 1843. combined.rand.k300. bin \
-logger File 1843. combined.rand.k300. log

This form of dimensionality reduction is unlikely to be as useful for training a deep neural network (DNN); however, it can be useful in similarity analysis in low dimensional feature space. Some of the datasets generated in this section will be referenced again in Section 7 to train classification machine learning models.

### Small Molecule Autocorrelation Descriptors

**FIGURE 5:**
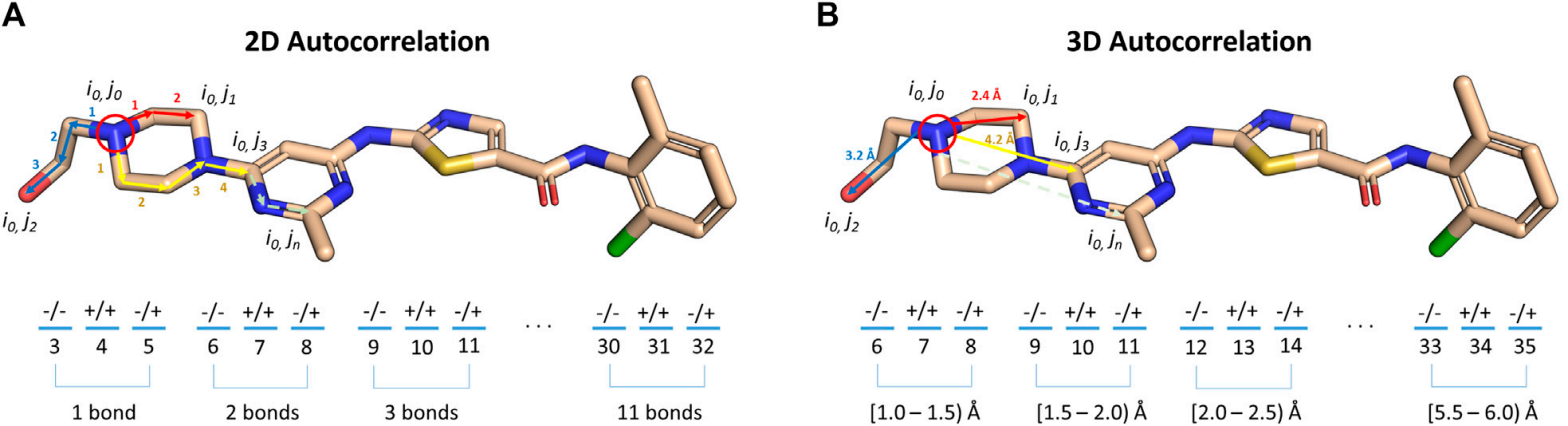
Illustration of signed autocorrelation descriptors. Signed autocorrelations are the sums of products of each atom property pair (e.g., i_0_,j_2_) in a distance bin defined by **(A)** bond separation, or **(B)** Euclidean distance in 3D space. Within each distance bin, atom property pairs are further separated into bins corresponding to the sign of the property of the first (left hand side of ‘/’) and second (right hand side of ‘/’) atoms in the pair.

As indicated in the previous section, the BCL can also compute signed autocorrelation functions. Autocorrelations are regularly used as features in cheminformatics machine learning models (Sliwoski et al., 2014). When computed for atomic descriptors, such as Atom_SigmaCharge, the autocorrelations sum pairwise property products into distance bins by calculating the separation between molecule atom pairs in number of bonds (2DA) or Euclidean distance (3DA). Each distance bin is further separated into three sign-pair bins corresponding to property value sign of each atom in the pair (Eq. 3) (Sliwoski et al., 2015).

$$A\left(r_{a},r_{b}\right)=\;\overset{N}{\underset{j}{\sum }}\overset{N}{\underset{i}{\sum }}\delta \left(r_{a}\leq r_{i,j}<r_{b}\right)P_{i}P_{j},$$ (3)

where r_a_ and r_b_ are the boundaries of the current distance interval, N is the total number of atoms in the molecule, r_(i,j)_ is the distance between the two atoms being considered, δ is the Kronecker delta, and P is the property computed for each atom. 2DAs are conformation-independent, while 3DAs are conformation-dependent (Figure 5).

The “dasatinibs.sdf” file contains the coordinates and connectivity for two dasatinib molecules: one with 2D coordinates, the other with 3D coordinates. Compute the signed 2DA and 3DA for Atom_SigmaCharge on both dasatinib molecules.

bcl.exe descriptor:GenerateDataset \
–source “SdfFile (filename = dasatinibs.sdf)” \
-feature_labels “Combine (3daSmoothSign (property = Atom_SigmaCharge))” \
-result_labels “Combine [Constant (999)]” -output dasatinibs.3da.csv \
–logger File dasatinibs.3da.log
bcl.exe descriptor:GenerateDataset \
–source “SdfFile (filename = dasatinibs.sdf)” \
-feature_labels “Combine [2DASmoothSign (property = Atom_SigmaCharge)]” \
-result_labels “Combine [Constant (999)]” -output dasatinibs.2da.csv \
–logger File dasatinibs.2da.log

**FIGURE 6:**
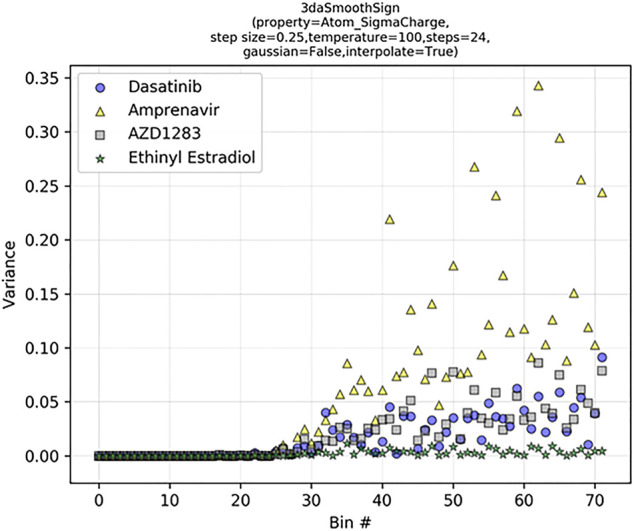
Signed 3DA variance increases with bin distance in flexible molecules. The 3DA distance bins extend to 6.0 Å at intervals of 0.25 Å. At each distance bin, there are three sign-pair bins (−/−, +/+, −/+).

Upon examination of the tabulated 2DA and 3DA values for the two different dasatinib molecules, we observe that the 2DA contains the same values in both cases, while the 3DA contains unique values for the different conformers. To visualize the variance in each 3DA distance bin, we can tabulate the 3DAs for Atom_SigmaCharge on an ensemble of 3D conformations for several different molecules (Figure 6). Dasatinib is a TKI with 7 rotatable bonds, amprenavir is a HIV protease inhibitor with 12 rotatable bonds, AZD1283 is an antagonist of the P2Y12 receptor with 9 rotatable bonds, and ethinyl estradiol is a synthetic estradiol with only 1 rotatable bond that binds and activates estrogen receptors.

We can see that the variance in each descriptor column increases as a function of distance and number of rotatable bonds. In ethinyl estradiol there is little change in descriptor column variance as a function of distance. In contrast, molecules with increasing numbers of rotatable bonds display increasingly large variances at longer distance bins. This suggests that increasing conformational heterogeneity at longer distance bins leads to increased noise. Indeed, we have previously found that extending LB 3DAs beyond approximately 6.0 Å generally results in reduced performance on QSAR classification tasks (Sliwoski et al., 2015), consistent with our example here (Figure 6). Importantly, however, at shorter distances where there is less conformational heterogeneity we are able to improve our performance with 3DAs even when the active conformation of the small molecule is unknown (Sliwoski et al., 2015; Mendenhall and Meiler, 2016). Moreover, models making predictions on molecules that are fairly rigid (e.g., steroid derivatives) may benefit from longer range distance bins.

It is also possible to use molecule:Properties to tabulate and compute statistics for molecules instead of plotting the CSV file data from descriptor:GenerateDataset. Here, we used descriptor:GenerateDataset to illustrate its usage. In practice, we do not just use a single 3DA or 2DA, but instead build sets of descriptors for feature and result labels and store them as separate code object files. As mentioned previously, the code object file format is the same format as allowed on the command line.

## Machine Learning Architectures and Applications

The BCL supports multiple machine learning algorithms for QSAR/QSPR modeling. Among the methods available are ANNs (including DNNs and multitasking neural networks) (Dahl, 2014; Bharath et al., 2015; Mendenhall and Meiler, 2016; Xu et al., 2017), support vector machines (SVM) (Kawai et al., 2008; Ma et al., 2008; Mariusz et al., 2009), Kohonen networks (KN) (Kohonen, 1990; Korolev et al., 2003; Wang et al., 2005), restricted Boltzmann machines (RBM) (Le Roux and Bengio, 2008; Tijmen Tieleman, 2008), and decision trees (DT) (Mariusz et al., 2009; Sheridan, 2012; Butkiewicz et al., 2013). GPU acceleration is available for ANNs and SVMs through OpenCL (Munshi, 2008). The primary application group for machine learning in the BCL is model. To see the applications within model, check the help menu:

bcl.exe model:Help

### Overview of BioChemical Library Model Training and Validation

Here, we will first introduce the user to the overall workflow involved in training, analyzing, and subsequently testing BCL machine learning models. The basic workflow for model training is the same for each machine learning method and can be completed via the model:Train application. To see the available machine learning methods, access the help options within model:Train.

bcl.exe model:Train --help

As of this writing, the available model types can be found in Table 3. The most reliable way to see available model types is via the help menu options of your version of the BCL.

**TABLE 3:** Machine learning model types.

Model Name	Description
Applicability Domain Kohonen	A Kohonen map-based implementation to detect whether a point is within the applicability domain of a model. All nodes will use the same spline for computing applicability. This implies an assumption that the model in question has the most difficulty predicting things far from any node center, regardless of which node center it is
Applicability Measure Kohonen	A Kohonen map-based implementation to detect whether a point is within the applicability domain of a model. All nodes will have their own distance metric, which is valid if the model is capable of distinguishing between classes of features (e.g., if the model in question is a Kohonen map itself)
Decision Tree	A decision tree trained using one of several methods to partition feature indices
Kappa Nearest Neighbor	A k-nearest-neighbor predictor; iteration optimizes k
Kohonen	A Kohonen-network based predictor
Leverage	Computes the leverage matrix (projection or hat matrix), which allows identification of significant outliers that would likely substantially influence any simple linear model of system. A returned value >2 represents probable outliers, while greater than 3 represent definitive outliers. The average value is 1 for all values in the training set
Linear Regression	Performs multiple linear regression
Multiple Output Support Vector Machine	A support vector machine with multiple outputs using sequential-minimal-optimization
Neural Network	A neural network with many customizable hyperparameters (e.g., hidden layer count, layer size, dropout type and fraction, transfer function, initialization with pre-generated models, learning rate, weight update/backpropagation scheme, etc.)
Restricted Boltzmann Machine	A restricted Boltzmann machine neural network
Support Vector Machine	A support vector machine trained using sequential-minimal-optimization

To expose all options for a particular machine learning method, pass the algorithm name as the first parameter to the application with the help menu request:

bcl.exe model:Train “<training algorithm>(help)”

The following is a typical command-line format to train a model beginning with a pre-generated descriptor binary file:

bcl.exe model:Train < training algorithm> \
-max_minutes < maximum time of training in minutes> \
-max_iterations < maximum number of training iterations> \
-final_objective_function < performance metrics for model evaluation> \
-feature_labels < names of descriptors> \
-training < training set> \
-monitoring < monitoring set> \
-independent < independent set> \
-storage_model < location in which to store the model> \
-opencl < enables GPU acceleration> \
-logger File < log file>

Model performance is evaluated with the user-specified objective function. The choice of objective function is typically related to the task being performed (e.g., classification vs regression) (Table 4).

**TABLE 4:** Objective functions for machine learning models.

Name	Prediction task	Formula
Accuracy	Classification	$Accuracy=\frac{TP+TN}{P+N}$
AUC (Area under the receiver operating characteristic curve)	Classification	$TPR=\frac{TP}{FN+TP}$
		$FPR=\frac{FP}{TN+FP}$
		AUC=∫TPR d(FPR)
LogAUC	Classification	$logAUC=\frac{\int_{0.001}^{0.1}TPR\;d\left(\text{log}\left(FPR\right)\right)}{\int_{0.001}^{0.1}d\left(\text{log}\left(FPR\right)\right)}$
MCC (Matthew’s correlation coefficient)	Classification	$MCC=\frac{TP*TN-FP*FN}{\sqrt{\left(TP+FP\right)\left(TP+FN\right)\left(TN+FP\right)\left(TN+FN\right)}}$
PPV (Positive predictive value)	Classification	$PPV=\frac{TP}{TP+FP}$
Enrichment factor	Classification	$EF\left(x\%\right)=\frac{PPV\left(x\%\right)}{PPV\left(100\%\right)}$
MAE (Mean absolute error)	Regression	$MAE=\frac{1}{N}\overset{N}{\underset{i}{\sum }}\left|f\left(x_{i}\right)-y_{i}\right|$
MAE_NMAD (MAE normalized by the mean absolute deviation)	Regression	$MAE_{NMAD}=\frac{MAE}{\frac{1}{N}\sum_{i}^{N}\left|y_{i}-\bar{y}\right|}$
RMSD (Root-mean-square deviation)	Regression	$RMSD=\sqrt{\frac{1}{N}\overset{N}{\underset{i}{\sum }}{\left(f\left(x_{i}\right)-y_{i}\right)}^{2}}$
NRMSD (RMSD normalized by the range)	Regression	$NRMSD=\frac{RMSD}{\max\left(y\right)-min\left(y\right)}$
RMSD_NSTD (RMSD normalized by the standard deviation)	Regression	$RMSD\_NSTD=\frac{RMSD}{\text{Stdev}\left(y\right)}$

BCL model:Train is designed to readily enable cross-validation. The application is flexible with respect to serialization of model predictions for each of the monitoring, independent, and training partitions as well as writing of the model itself. For example, in five-fold cross-validation, the dataset is split into five chunks. For each round of cross-validation, the model is trained on four-fifths of the dataset, and the other fifth “independent” set is left out for testing. One of the chunks can additionally be specified as the monitoring dataset. The monitoring dataset can be used for early termination of the model training session to prevent overtraining (early termination is largely deprecated in favor of dropout to prevent earlier termination; we demonstrate it here to illustrate the syntax).

The initial dataset set is split into monitoring, independent, and training partitions with model:Train by assigning chunks with the dataset retriever responsible for binary format files, Subset. In the following pseudocode example, we will set the options to divide the training set into the following five chunks (0-indexed): chunks one to four will be used as the training set, and chunk 0 will be used as both the monitoring set and the independent set (this is appropriate only if the monitoring dataset is not being used for early termination).

-training “Subset (number chunks = 5,chunks = [1, 4], filename=<my_dataset.bin>)”
-monitoring “Subset (number chunks = 5,chunks = [0], filename=<my_dataset.bin>)”
-independent “Subset (number chunks = 5,chunks = [0], filename=<my_dataset.bin>)”

Dataset partitioning is repeated for each round of cross-validation until each chunk takes a turn as the independent set. Then, the predictions of all the test sets are pooled together by the model:PredictionMerge application:

bcl.exe model:PredictionMerge \
-input_model_storage ‘File (directory = /path/to/models/,prefix = model)' \
-output < output_pooled_predictions>

This command line averages predictions made on the same independent set, though other pooling operations are available (see help). Prediction performance is evaluated with the specified objective function on the pooled predictions using the model:ComputeStatistics application:

bcl.exe model:ComputeStatistics \
-input < output_pooled_predictions> \
-obj_function < performance_metric> \
-filename_obj_function < output_performance_metric_file>

### Simplifying the Model Training and Validation Framework in Practice

To simplify model training, we have written a Python script “launch.py” to perform training and cross-validation with one command.

To see a list of model training operations (descriptor selection or scoring, for example):

/path/to/bcl/scripts/machine_learning/launch.py–h

To see the list of available flags for cross-validation, call

/path/to/bcl/scripts/machine_learning/launch.py–t cross-validation–h

The following pseudocode example generates a simple linear regression model:

/path/to/bcl/scripts/machine_learning/launch.py -t cross-validation \
--cross-validation 5 --local \
--learning-method LinearRegression (objective function = RMSD, \
solver = Cholesky (smoothing = 0)) \
--id linear_regression --final-objective-function RMSD \
--datasets < my_dataset.bin > --override-memory-multiplier: 1.25

More complex commands can be easily prepared inside of a configuration file to be passed to the “launch.py” script. A sample configuration file is available in the Supplementary Material.

bcl/trunk/scripts/machine_learning/launch.py–t cross_validation \
--config-file config. example.ini

The “launch.py” script will automatically generate three new directories titled “log_files”, “results”, and “models”. Into each of those three directories a labeled directory (name specified with the id flag) is made. Model prediction output files and results of the final objective function are stored in the labeled directory within the “results” folder. Log files, commands, and autogenerated scripts are stored in the labeled directory within the “log_files” folder. Finally, final model details are stored in the labeled directory within the “models” folder.

In addition to running the training jobs locally, training can be run on a SLURM cluster using the slurm flag. In this way, large cross-validation jobs may leverage high-performance computing with minimal changes to the configuration. See additional configuration operations, such as slurm-host, using launch. py–t cross-validation–h.

### Applying Models to Independent Test Sets for Virtual High-Throughput Screening

Note that in the above examples the training and test splits are derived from the same binary format file. This is not strictly necessary, and the user can supply alternatively derived validation splits prepared in separate files. Moreover, using a dataset split as the independent test set is generally only useful for model validation. To apply trained model predictions to new molecules in a vHTS, either model:Test or molecule:Properties can be used. For example, if a model is trained and validated using five-fold cross-validation, then the merged prediction on an external test set can be made as follows with model:Test:

bcl.exe model:Test \
-retrieve_dataset “Subset (filename=<vHTS.test.bin>)” \
-storage_model “File (directory = /path/to/models/,prefix = model)” \
-average output < vHTS.model_test.csv> –logger File < vHTS.model_test.log>

Likewise, predictions can be made with molecule:Properties using the Prediction operators:

bcl.exe molecule:Properties–input_filenames < vHTS.test.sdf> \
–tabulate \
“Define {predicted_activity = PredictionMean [storage = File (directory = /path/to/models/,prefix = model)])}” predicted_activity \
“Define {local_ppv = PredictionInfo [predictor = File (directory = /path/to/models/,prefix = model),metrics (LocalPPV)]}” local_ppv \
“Define {XActive = Multiply [predicted_activity, Greater (lhs = local_ppv,rhs = 0.50)]}” XActive \
-output_table < vHTS.prop.test.csv > -logger File < vHTS.prop.test.log>

Notice that scoring new compounds via molecule:Properties allows multiple outcome metrics to be reported and modified on-the-fly, while scoring with model:Test just outputs the raw prediction values (and optionally just the mean with average). In this case, the output of model:Test is equivalent to “predicted_activity” from molecule:Properties. The property “XActive” is the “predicted_activity” score when the local PPV is greater than 0.5, and 0.0 otherwise. The localPPV metric calibrates model output values to local classification probability on the test sets. It is an estimate of the PPV at a singular model output value. This is in contrast to traditional PPV, which specifies the value of a prediction at, or above, a given output value (assuming positive parity). This metric assumes that the trained model prediction value varies monotonically with the actual prediction likelihood.

### Supervised Learning

#### Training a Standard Artificial Neural Network to Classify Kir2.1 Positive Allosteric Modulators

ANNs are one of the most commonly employed classes of non-linear classifiers in QSAR modeling for LB-CADD due to their strong predictive power (Dahl, 2014; Xu et al., 2017; Vamathevan et al., 2019). To see all the options available to a neural network in the BCL, call

bcl.exe model:Train “NeuralNetwork (help)”

The BCL supports shallow and deep single- and multi-tasking neural networks. Transfer functions include linear, sigmoid, rectified linear, and leaky rectified linear. For a network with L hidden layers indexed l∈(1…L) , forward propagation for l∈(0…L−1) can be described as

$$z^{\left(l+1\right)}=\;w^{\left(l+1\right)}y^{l}+b^{\left(l+1\right)},$$ (4)

$$y^{\left(l+1\right)}=f\left(z^{\left(l+1\right)}\right)$$ (5)

where $y^{l}$ is the output vector at layer *l* connected to the input vector $z^{\left(l+1\right)}$ at layer *l+1* by weights ***w*** and biases ***b***, and *f* is the transfer function applied to each set of inputs into the *l+1* layer. Correspondingly, the activation of a single neuron *i* in hidden layer *l+1* can be represented as

$$z_{i}^{\left(l+1\right)}=\;w_{i}^{\left(l+1\right)}y^{l}+b_{i}^{\left(l+1\right)},$$ (6)

$$y_{i}^{\left(l+1\right)}=f\left(z_{i}^{\left(l+1\right)}\right)$$ (7)

to yield the output $y_{i}^{\left(l+1\right)}$ from layer *l+1*. We have found that for classical QSAR tasks a simple mean-squared error (MSE) cost function is adequate.

Historically, overtraining in ANNs has been prevented by early termination of training when the monitoring dataset improvement rate or improvement scores fail to progress beyond a pre-determined extent. More recently, we have demonstrated that dropout is a better alternative to prevent model overtraining in QSAR tasks (Mendenhall and Meiler, 2016). The dropout approach has been described elsewhere in detail (Nitish et al., 2014). Briefly, during forward propagation each layer of the ANN is assigned a probability p according to which the output value $y_{i}^{l}$ of each i neuron in the layer l will be independently set to zero (i.e., “dropped”).

$$z_{i}^{\left(l+1\right)}=\;w^{\left(l+1\right)}\left(r^{l}*y^{l}\right)+b_{i}^{\left(l+1\right)},$$ (8)

Here, $r^{l}$ is a vector with the same dimensions as $y^{l}$ whose values are either 0 (at fraction *p*) or 1 (at fraction 1—*p*) and multiplied elementwise by the values in $y^{l}$ . At the end of every training batch, $r^{l}$ is shuffled. If neurons are dropped with a probability *p*, then at test time the corresponding weights are scaled down by the factor 1—*p*.

Train a shallow (single hidden layer) neural network to classify molecules as either active or inactive PAMs of Kir2.1 beginning with the randomized dataset we generated in Section 6.2:

launch.py -t cross-validation --local \
--datasets 1843. combined.rand.bin --id 1843. ann.1x32_005_025 \
--config-file config. example.ann.ini \

The configuration file specifies the learning method as follows:

learning-method: ‘NeuralNetwork ( \
transfer function = Sigmoid, \
weight update = Simple (alpha = 0.50,eta = 0.05), \
dropout (0.05,0.25), \
objective function = % (objective-function)s, \
scaling = AveStd, steps per update = 1, hidden architecture (32), \
balance = True, balance target ratio = 0.10, \
shuffle = True, input dropout type = Zero \
)’

Note that we are asking for an ANN with one hidden layer composed of 32 neurons. The input and hidden layers will have 5 and 25% dropout, respectively. In addition, we have enabled class balancing. We have far fewer active (172) than inactive (301,321) compounds. Balancing oversamples the underrepresented (minor) class to achieve a ratio of (in this case) 0.10 with the most common class (major). The balance max repeats flag can also be set to specify the maximum number of times that a feature can be repeated. This does not lead to overtraining because of dropout. Batch size is controlled with the steps per update flag. The objective-function variable is defined in the configuration file as

“AucRocCurve (cutoff = 0.5,parity = 1,x_axis_log = 1, min fpr = 0.001, max fpr = 0.1)”

Additional variables, such as the maximum number of training iterations (20), number of rounds of cross-validation (5), monitoring dataset (independent set), etc. are also set in the configuration file.

As a comparison, train an additional ANN with the same parameters using the feature set whose dimensions were reduced with PCA in Section 6.2:

launch.py -t cross-validation --local \
--datasets 1843. combined.rand.pca_095. bin \
--id 1843. pca_095. ann.1x32_005_025 \
--config-file config. example.ann.ini \

**FIGURE 7:**
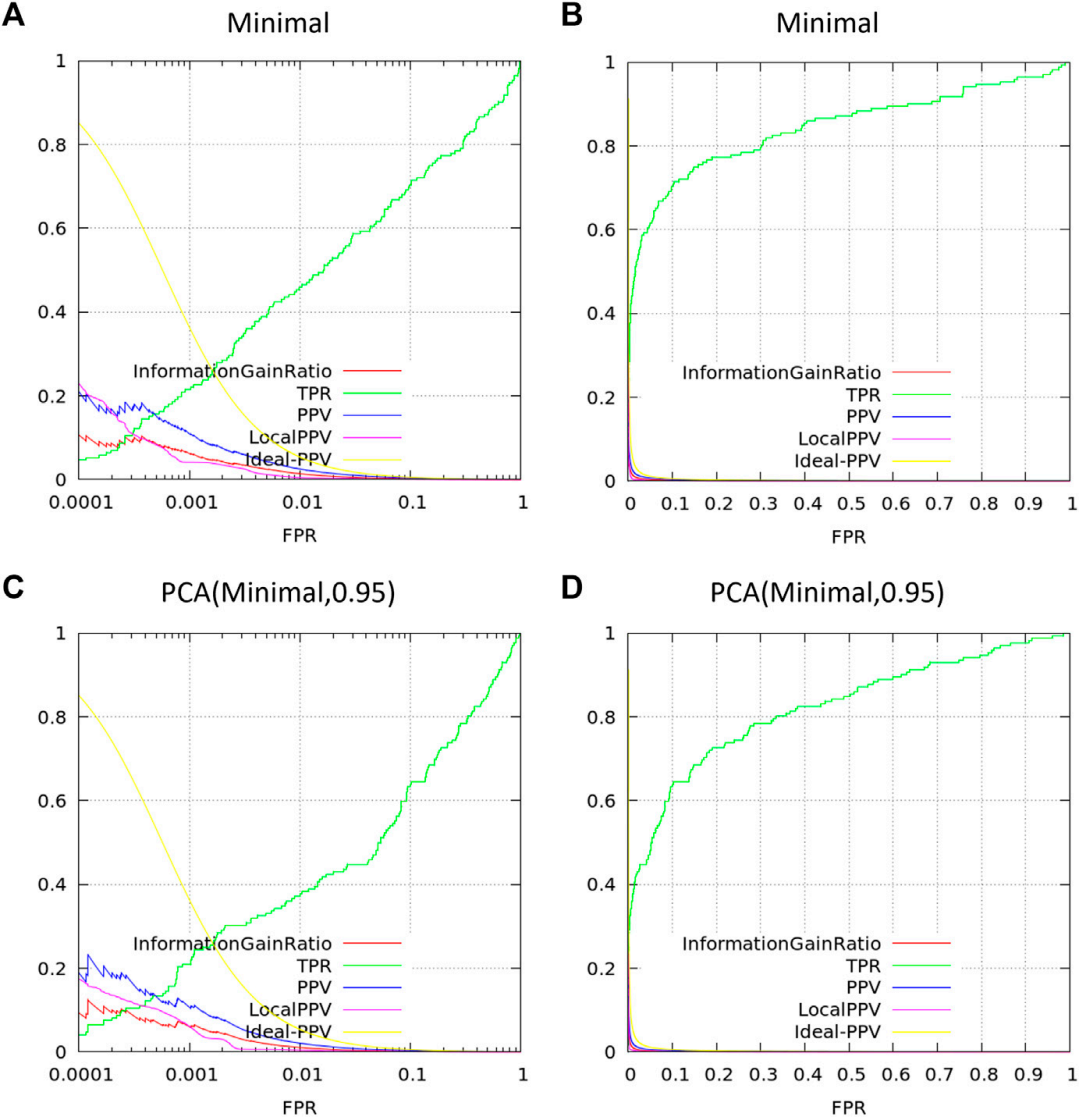
ROC curve comparison Kir2.1 activity prediction models with different descriptors. Models were trained with either **(A,B)** the Minimal dataset containing 959 non-redundant standard LB descriptors (see Supplemental Data) on a log10 **(A)** or linear **(B)** x-axis, or PCA-modified LB descriptors accounting for 95% variance on a log10 **(C)** or linear **(D)** x-axis.

The “launch.py” pipeline automatically generates a ROC curve for each model with and without a log scaled x-axis (Figure 7). The overall AUC is quite similar between the two methods (Figures 7B,D); however, the model trained with the PCA descriptors has worse early enrichment (logAUC = 0.39) than the model trained with the full descriptors (logAUC = 0.46) (Figures 7A,C).

#### Training a Deep, Multitasking Neural Network to Predict Solubility

Predicting physicochemical properties such as solubility is a challenging but critical component of lead compound optimization. Many substitutions to a candidate molecule may increase the potency or selectivity, but at the cost of worsening solubility, metabolic stability, or other properties. Therefore, it is advantageous to prioritize synthesis and evaluation of derivatives that are simultaneously predicted to be active and have a promising chemical profile. To do this, we need a target-agnostic QSPR model.

Dahl and colleagues demonstrated that multitask learning could improve the prediction of multiple outputs simultaneously if the training tasks are correlated (Dahl, 2014; Xu et al., 2017). As an example of how such a model is trained with the BCL, we will train a deep neural network to simultaneously predict three measures relating to solubility: the water-octanol partition coefficient (logP), the aqueous solubility (logS), and the hydration free energy (i.e., the solvation free energy in water; ΔG_hydration_). Note that not the descriptors, model architecture, nor hyper-parameters have been optimized for performance. This can be seen as an “out of the box” model a user might create.

Molecules for training and validation are sourced from previously published databases (Syracuse Research Corporation, 1994; Edward W.; Lowe et al., 2011; Mobley and Guthrie, 2014; Wu et al., 2018) and combined with BCL molecule:Unique to remove redundant compounds (see Supplementary Methods for details). Note that we anticipate some additional error in predictions introduced by not averaging replicate experimental measurements of QSPR properties prior to removing redundancy. Generate three datasets: One with all of the unique compounds (Full), another that contains only those compounds with all three result labels (Dense), and one that contains all of the compounds minus those with all three result labels (Full–Dense). The following command generates the feature set for all of the compounds with three result labels encoded by MDL property labels:

bcl.exe descriptor:GenerateDataset \
-source “SdfFile (filename = all_logp_logs_dgsolv.sdf.gz)” \
-feature_labels VuMendenhallMeiler2019. Scalar_Mol2D.object \
-result_labels “Combine (LogP_actual, LogS_actual,dG_hydration_kcal-mol)” \
-output all_logp_logs_dgsolv.Scalar_Mol2D.bin \
-logger File all_logp_logs_dgsolv.Scalar_Mol2D.log \
-scheduler PThread 8 –compare

**FIGURE 8:**
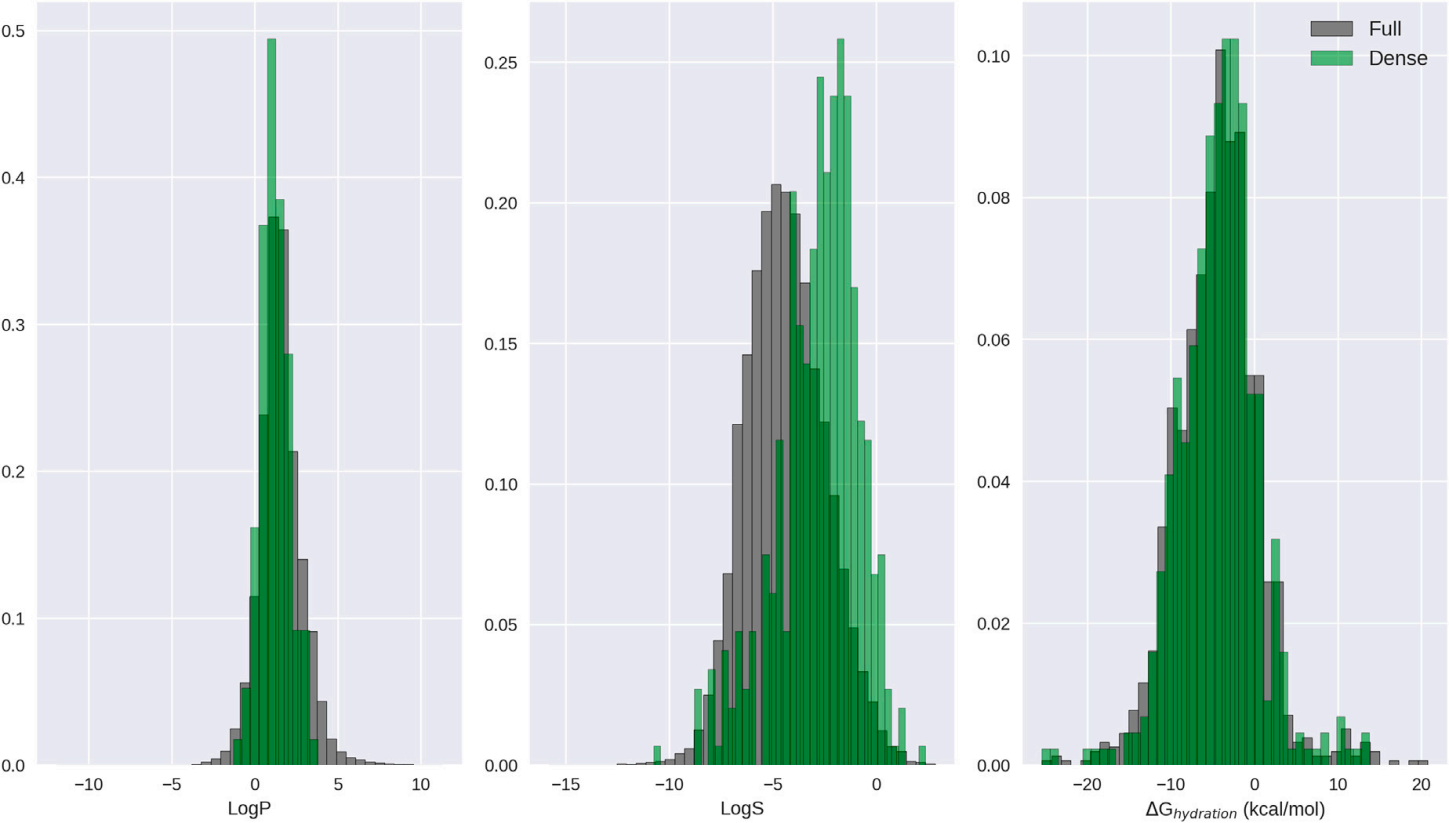
Result value overlap between the Full and Dense datasets. Density normalized histograms of LogP, LogS, and ΔG_hydration_ between the Full (35874 total, 35113 LogP, 20721 LogS, 1,339 ΔG_hydration_; gray) and Dense (448 total for all values; green) datasets.

To generate the Dense feature set, add the–forbid_incomplete_records flag. The two binary format files should contain 35,874 and 448 rows, respectively, and the third dataset should contain the difference between them, 35,426. The distribution of result values overlaps reasonably well between the Full and Dense datasets, with the exception of the LogS distributions (Figure 8).

Randomize the datasets before training the model. The configuration file config. exmple.mdnn.ini sets up the neural network architecture:

learning-method: “NeuralNetwork ( \
transfer function = Rectifier (0.05), \
weight update = Simple (alpha = 0.50,eta = 0.005), \
dropout (0.05,0.25, 0.05), \
objective function = % (objective-function)s, \
scaling = AveStd, steps per update = 10, hidden architecture (128,32), \
balance = False, shuffle = True, input dropout type = Zero \
)”

Note that our network contains 2 hidden layers with 128 and 32 neurons, respectively, with 5% dropout on the input layer, 25% dropout on the first hidden layer, and 5% dropout on the second hidden layer. Our objective function will be MAE_NMAD since this is a regression task. We will perform five-fold cross validation (specified in the configuration file). Train the network:

launch.py -t cross-validation --local \
--datasets all_logp_logs_dgsolv.Scalar_Mol2D.rand.bin \
--id all_logp_logs_dgsolv.Scalar_Mol2D.2x256-32_005_025_005 \
--config-file config. example.mdnn.ini --just-submit

The just-submit flag sends the process to the background. Train the dense network as well; it should take less time since there are relatively few examples in the training sets. Check the log_merge.txt file in the corresponding “log_files” subdirectory to view the final objective function for each of the three result labels (Table 5).

**TABLE 5:** Five-fold cross validation results for multitask modeling of solubility prediction. These table values are automatically calculated and output in the log_merge.txt file in the corresponding subdirectory of the autogenerated “log_files” directory. The Full set consisted of 35,874 molecules (with 35113 LogP, 20721 LogS, and 1,339 ΔG_hydration_ result labels). The Dense set consisted of 448 molecules (with 448 LogP, 448 LogS, and 448 ΔG_hydration_ result labels). The Full–Dense set contained 35,428 molecules (with 34665 LogP, 20273 LogS, and 891 ΔG_hydration_ result labels).

		QSPR Prediction								
		*LogP*			*LogS*			*ΔG*_*hydration*_		
	**Analysis Metric**	***MAE***	***MAD***	***MAE/MAD***	***MAE***	***MAD***	***MAE/MAD***	***MAE***	***MAD***	***MAE/MAD***
**Model Feature Set**	*Full*	0.61	0.95	0.64	0.21	1.51	0.14	1.64	3.62	0.45
	*Dense*	0.20	0.68	0.29	0.24	1.51	0.16	1.53	3.58	0.43
	*Full—Dense*	0.51	0.97	0.53	0.23	1.51	0.15	2.03	3.85	0.53

In cases where the training set has small deviation from the mean value, MAE will be lower, which can be misleading. To address this, we normalize MAE by MAD. Here, we see that the model trained on the Dense set of features learned LogP the best. However, this may be an artifact of the reduced training space. If we were to evaluate whether the Dense model was able to extrapolate beyond the very small training set, we would almost certainly see worse performance.

To illustrate this, evaluate the predictive power of our Dense model on molecules in our Full–Dense training set, and vice versa. This can be accomplished using either model:Test or molecule:Properties as described in Section 7.1. The results of this analysis are in Table 6. The model trained on the Full–Dense set does a good job predicting the QSPR properties for the Dense molecule set, achieving Pearson correlation coefficients between 0.82 and 0.99 for the three tasks. We see that the values we obtained in the internal random-split 5-fold cross validation (Table 5) agree with those obtained on the Dense set predictions (Table 6). In contrast, despite having the best five-fold cross-validation performance (Table 5), the model trained on the Dense feature set performs extremely poorly at predicting quantitative QSPR properties of the Full–Dense molecule set (Table 6).

**TABLE 6:** QSPR external test-set predictions. Results of predicting QSPR properties on the Dense dataset with the model trained on the Full–Dense feature set, and results of predicting QSPR properties on the Full–Dense dataset with the model trained on the Dense feature set. The table is organized such that the values indicate the performance of the model trained with the indicated set of descriptors on the alternate test set. The Dense set consisted of 448 molecules (with 448 LogP, 448 LogS, and 448 ΔG_hydration_ result labels). The Full–Dense set contained 35,428 molecules (with 34665 LogP, 20273 LogS, and 891 ΔG_hydration_ result labels).

		Model Feature Set					
		*Full—Dense*			*Dense*		
	QSPR Prediction	*LogP*	*LogS*	*ΔG*_*hydration*_	*LogP*	*LogS*	*ΔG*_*hydration*_
**Analysis Metric**	*MAE*	0.94	0.27	1.71	580.32	65.18	30.03
	*MAE/MAD*	1.37	0.18	0.48	599.81	43.20	7.80
	*R*	0.88	0.99	0.82	0.00	-0.11	-0.05
	*p*	0.89	0.99	0.88	0.48	0.88	0.75

Taken together, these data suggest that there is likely a significant fraction of molecules in the Full–Dense set that occupy an area of feature space not represented in the 448 molecule Dense set. This is a good example that internal randomized cross-validation on a small training set is not an accurate predictor of external test set performance unless the external test set is within a similar domain of applicability (Tetko et al., 2008; Sheridan, 2012). Applicability domains in the BCL will be discussed in more detail in Section 7.5.

#### Training a Decision Tree

DT is a tree-based machine learning algorithm that partitions the dataset into smaller subsets as it develops. A DT starts from a root node, branches out to internal nodes, and ends at leaf nodes. To see the different options of a decision tree, call

bcl.exe model:Train “DecisionTree (help)”

The default option of the decision method chooses the features for data splitting with the maximum information gain, and its prediction performance is scored by accuracy.

learning-method: DecisionTree ( \
objective function = Accuracy, \
partitioner = InformationGain, \
Activity cutoff = 0.5, \
nodes core = SplitRating, \
min split = 0 \
)

There are two factors that determine the order of features and their corresponding splitting values in dataset partitioning in a decision tree: partitioners and node scores. Four types of partitioners are currently implemented in the BCL: InformationGain, Gini, ROC, and Sequence. The first three options rate the feature to split the dataset by information gain, Gini index, and area under the curve of the local ROC curves (Ferri et al., 2002), respectively. The last option only allows splits that result in at least one pure node.

While the partitioner determines how to calculate the split rating of different configurations of dataset partition, the node score type dictates how to rank different combinations of feature order and their corresponding splitting values. Four types of node scores are currently implemented in the BCL: split rating (SplitRating), number of correct predictions before splitting (InitialNumIncorrect), split rating times initial number of correct predictions (RatingTimesInitialNumIncorrect), and sum of number of incorrect predictions before and after data splitting (InitialIncorrectPlusFinalCorrect). The users can also control the minimum number of incorrect classifications of a node by assigning a value to the min split flag.

A DT was employed in Section 3.4 to classify small molecules’ potential for hit optimization. The BCL can convert DTs into descriptor files that can be used to help defined new properties. For more details, see Section 3.4.

### Unsupervised Learning

#### Adjusting Tunable Parameters in a Self-Organizing Map

A self-organizing map (SOM), also commonly referred to as a Kohonen map, is an unsupervised learning method that is commonly used in clustering and dimensionality reduction. The SOM produces a low-dimensional (typically one to two dimensions), discretized representation of the input space of the training samples, called a map. This method applies competitive learning to reach a solution, as opposed to conventional feed-forward neural networks, which utilize error-correction learning. To see the options available to a Kohonen map model, call

bcl.exe model:Train “Kohonen (help)”

Here is the typical configuration file setup to build a Kohonen map model:

learning-method: Kohonen (
shuffle = True, scaling = AveStd, map dimensions = (10, 10), \
steps per update = 0,radius = 7.5, length = 140, Neighbor kernel = Bubble, \
Initializer = RandomlyChosenVectors, cutoff = 0.5, objective function = RMSD \
)

Before training a Kohonen map, users may shuffle the training set (shuffle = True). Similar to the ANNs, there are two options for scaling the input: AveStd and MinMax. The former works best when the input descriptors are continuous, and the latter is ideal for sparse and/or discretized input data. Regarding the configuration of the SOM, the map dimensions option dictates the number of nodes, or neurons, in each direction of the map. Setting the steps per update flag (i.e., batch size) to 0 indicates that all training rows will be used for each iteration.

The initial radius of the neighborhood function, radius, is the maximum distance between the neighbor neuron and the best matching unit (BMU). Increasing the radius generally increases model quality at the expense of training time. In our experience, diminishing returns are met when the radius approaches 1/3 to 1/2 the total distance of the map. The number of iterations it takes for the radius to decrease to 0 in the given neighbor kernel function is given by length. The radius of the neighborhood is gradually reduced as the number of the iterations t increases, such that by 4∗length the original radius is reduced to size 0:

$$radius_{t+1}=radius_{t=0}\left(1-\frac{t+1}{4\times length}\right),$$ (9)

Each iteration, the neurons compete by measuring their distances to the input dataset. The neuron j, with associated weight vector w, with the lowest distance d to the randomly selected input vector x is the winner.

$$d_{j}\left(x\right)=\sqrt{\underset{i}{\sum }{\left(x_{i}-w_{ji}\right)}^{2}},$$ (10)

Iterations proceed for the entire batch size prior to updating neuron weights. The next step is updating the weights within the neighborhood of the winning node. There are two options for the neighbor kernel function: Bubble and Gaussian. The new weights are updated as

$$w_{ij}^{t+1}=w_{ij}^{t}+\alpha_{j}^{t}B_{j}\left(x_{i}^{t}-w_{ij}^{t}\right),$$ (11)

where the β is 0.8 for the wining node and 0.2 for other nodes in the neighborhood and learning rate α is $exp(-\frac{{\left(distance\;to\;winner\right)}^{2}}{2\times radius^{2}})$ for the Gaussian kernel and 1 for the Bubble kernel. The Bubble kernel keeps the learning rate constant inside the neighborhood, while the Gaussian kernel reduces the learning rates for more distant nodes, at a substantial performance cost.

Finally, users can select one of the objective functions mentioned above to evaluate the prediction performance of the model. At test time, the model will assign an AD score for each external compound. This AD score is the normalized distance of that compound to the closest node of the training set. For instance, a tested molecule with an AD score of 0.90 is further from the closest node than 90% of other molecules in the training set. In other words, that molecule’s feature space was not so well-represented in the training dataset.

#### Training a Self-Organizing Map Druglikeness Applicability Domain

We will use the BCL to build class-specific druglikeness applicability domain (AD) models from the structures of FDA approved drugs: 58 opioid receptor modulators and 82 kinase inhibitors (Wishart et al., 2018). From each set of molecules, 5 molecules are randomly removed from the training set for external validation. Training occurs on the remaining molecules. The AD models will be used to measure the similarity between external compounds and a “typical drug” targeting opioid receptors or kinases. Generate a configuration file for the AD called AD. config containing the following:

learning-method: “ApplicabilityDomainKohonen ( \
shuffle = 0, map dimensions (% (cluster_num)s), steps per update = 0, \
length = 140, radius = 7.5, neighbor kernel = Bubble, \
initializer = RandomlyChosenVectors, scaling = AveStd, cutoff = 0.5, \
share distance metric = True
)”

Note that the map dimensions are set by the cluster_num flag in the training command. Generate feature set for each molecule file using descriptor:GenerateDataset. Train the kinase set AD model:

launch.py -t cross_validation --config-file AD. config \
--datasets kinase. train.Scalar_UMol2D.bin \
--id kinase. Scalar_UMol2D.AD --max-iterations 200 \
--local --no-cross-validation --cluster_num 5

Afterward, train the opioid receptor set AD model. Next, we can evaluate the test sets with each AD model, beginning with the kinase inhibitor test set with the kinase inhibitor AD model:

bcl.exe model:Test -retrieve_dataset \
“SdfFile (filename = kinase.test.sdf.gz)” \
-storage_model \
“File (directory = ./models/kinase_mol2d_scalar_AD, prefix = model)” \
-output kinase_kinaseAD.test.out

**FIGURE 9:**
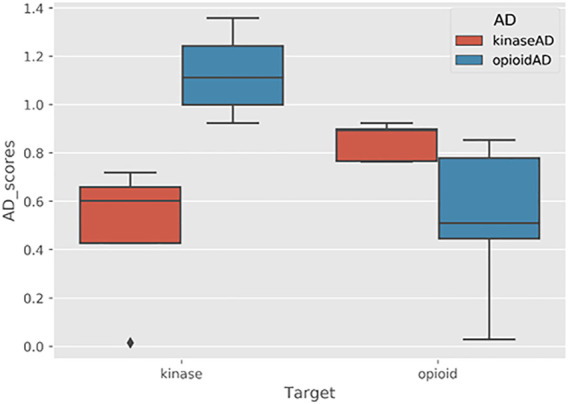
Applicability domain models differentiate molecular structures targeting unique proteins. Each box plot represents AD scores of five drugs that target either kinases or opioid receptors. AD models trained on kinase and opioid training datasets are colored in red (legend: kinaseAD) and blue (legend: opioidAD), respectively.

The AD scores are listed in the output data file. The first two lines are the format name, and the dimension of the data table. The AD scores of five test compounds are stored in the second columns of the last 5 lines. We can see that our test set compounds from the FDA approved kinase inhibitor list have a shorter AD distance than our molecules in the opioid receptor test set, and vice versa (Figure 9). These scores represent the distance of each test compound to the feature space occupied by the training set FDA approved kinase inhibitors. In other words, they tell us how far we are from drug-like feature space for this group of inhibitors. The output AD scores are summarized in Figure 9.

## Drug Design

Up to this point we have demonstrated vHTS predictions on pre-existing external datasets. Screening external datasets can be very valuable because of the ever-increasing number and availability of public, commercial, and institutional small molecule repositories. Nevertheless, it is also frequently the case that computation can be applied to assist specific medicinal chemistry projects. For example, in silico drug design can conceivably be utilized for library design, hit explosion, or scaffold hopping. Here, we will demonstrate how to perform multicomponent reaction (MCR)-based drug design with the BCL.

### Defining Reaction Files for Drug Design

Reaction-based drug design in the BCL proceeds according to user-defined MDL RXN (.rxn) files. There are a number of predefined reactions located in bcl/rotamer_library/functional_reactions. Reactions can be single-component intramolecular reactions, or multi-component intermolecular reactions of up to four unique reagents. Reactants must have their atoms mapped to corresponding atoms in the product(s). Atom mapping is required for substituents on the input reagents to be merged with the product(s).

The reaction design framework functions in part by performing substructure comparisons of candidate reagents to reactant structures drawn in the RXN file. Substructure matching occurs at a resolution of ElementType for atoms and BondOrderOrAromatic for bonds. If there are candidate reagents that collectively can match all reactant positions in a reaction, then the reaction can proceed. Note that unlike input SDFs for molecule files, aromaticity must be shown explicitly in the RXN file to be interpreted. Also note that reactant matching will only match hydrogen atoms if they are drawn explicitly.

### Executing Reaction Design

**FIGURE 10:**
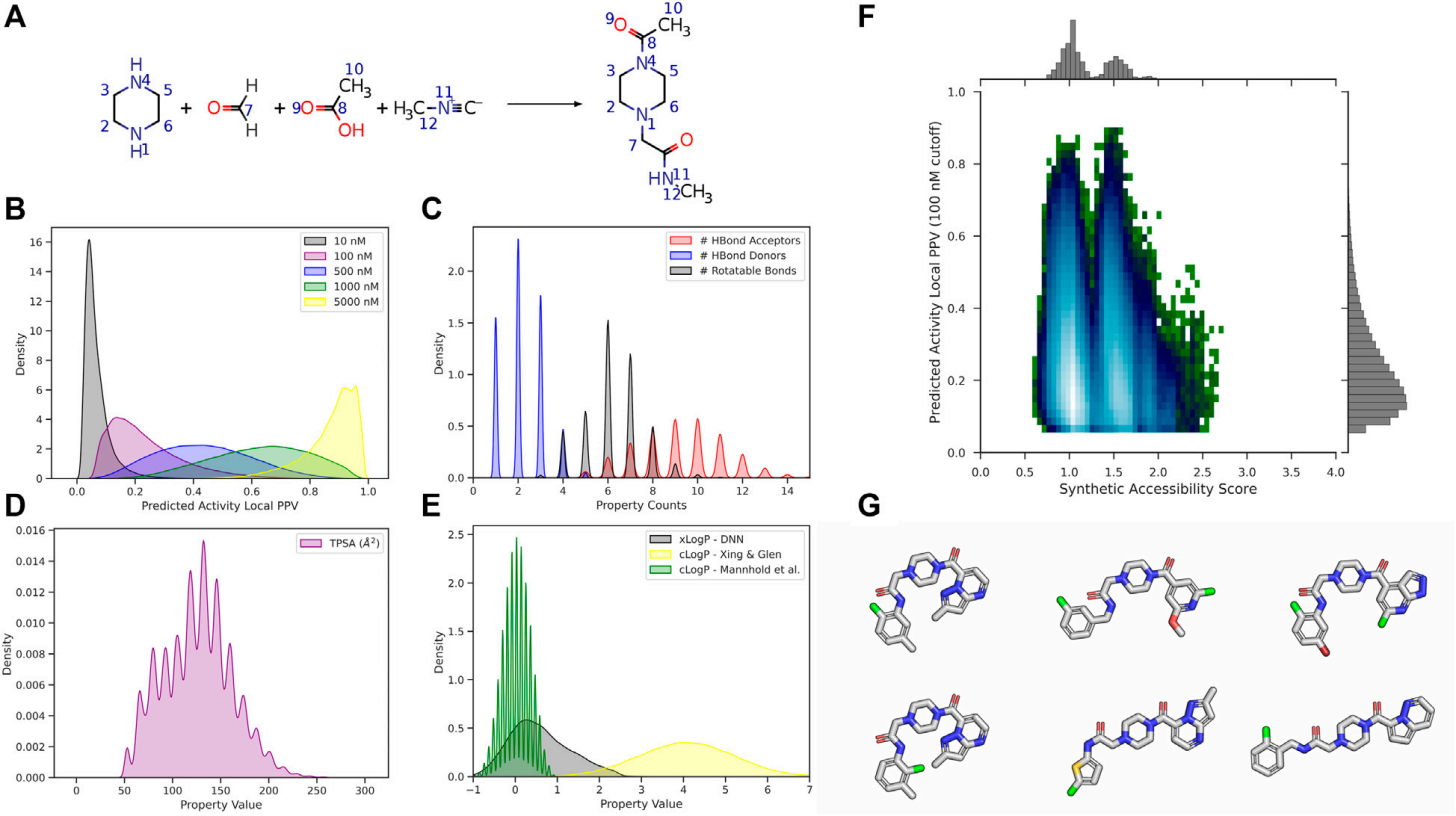
Multicomponent reaction-based design of dopamine receptor D4 antagonist candidates. **(A)** The 4-component split-Ugi reaction utilizing a piperazine as the diamine. Hydrogen atoms are represented implicitly. Atom numbers correspond to mappings between reactant and product atoms. The density of molecules generated with respect to **(B)** the predicted activity local PPV for each classification result label, **(C)** number of hydrogen bond donors, hydrogen bond acceptors, or rotatable bonds, **(D)** topological polar surface area, and **(E)** the computed logP. LogP estimates are computed using the Full neural network from Section 7.4 (XLogP; gray), the property-based cLogP approach from Xing and Glen, 2002 (Xing and Glen, 2002), and the atom-based cLogP approach from Mannhold et al., 2008 (Mannhold and Van de Waterbeemd, 2001). **(F)** Density of generated molecules with respect to synthetic accessibility score (x-axis) and predicted dopamine receptor D4 antagonist activity (local PPV for 100 nM classification; y-axis). The 2D histogram density is log10-scaled. **(G)** Structural representation of six randomly selected molecules from a sample of 198 designs that had local PPV values greater than or equal to 0.80 for predicted activity at 100 nM.

In this example, we will generate products according to a 4-component split-Ugi reaction utilizing piperazine as the diamine scaffold in all designs (Figure 10A).

bcl.exe molecule:React \
-starting_fragments piperazine. sdf -reagents reagents_le_20. sdf \
-reactions./rxns_dir/ -routine Random -repeats 9 -ligand_based \
-fix_geometry -fix_ring_geometry -extend_adjacent_atoms 2 \
-output_filename ugi_products.sdf -logger File ugi_reaction.log

The individual molecule fragments passed via starting_fragments are treated as required reaction components. The reactions flag is given the path to a directory containing all RXN files the user wishes to include in the reaction. The reagents flag specifies candidate reactants with which the starting_fragments molecules are reacted. Thus, for every entry in the SDF passed via starting_fragments, the molecule: React application will check to see if it is a valid reactant for any of the reactions in the directory specified by reactions; for those reactions that the current starting_fragments molecule is a valid reactant, the remaining possible reactant positions are fit against the molecule fragments provided via reagents.

The routine flag specifies how to continue with reaction sampling. Currently, there are two options, though additional options are under development. The default is Random, which will perform one valid reaction (if any exist) for each molecule in starting_fragments using a randomly selected reaction and reagents from the user input. By default, the Random routine will run one time; however, by specifying repeats users can increase the number of cycles. If the starting_fragments SDF contains 100 entries and repeats is set to 4, then the molecule:React application will run 500 times–one initial run for all entries followed by four repeats of all 100 entries. Alternatively, users may specify Exhaustive, which will enumerate all possible products from all given reactions and reagents for each starting_fragments molecule. Ongoing efforts to expand the reaction-based drug design framework include additional optimization routines, such as evolutionary fragment generation and simulated annealing, as well as mixed intra- and inter-reagent reactions. Other options are related to generation of 3D conformers for the product molecules and are explained in the help menu.

bcl.exe molecule:React --help

### Analyzing Designs

For illustration purposes, we generated ∼700,000 configurationally unique molecules with the split-Ugi reaction (Figure 10A). As our starting fragment, we used a solitary piperazine ring. For simplicity and to keep the size of the product library reasonably small, we also utilized a formaldehyde in the second reactant position (though another aldehyde is possible). We passed a collection of commercially available building block fragments, filtered such that the heavy atom count was less than or equal to 20, to fill positions three and four via our reagents flag. We analyzed the resulting library without any additional filtering (e.g., for druglikeness, predicted mutagenicity, Lipinski’s rules, etc.).

Piperazine rings and related substructures are well-defined core components of dopamine receptor (DR) antagonists (Lindsley and Hopkins, 2017). Utilizing BCL commands described in previous sections along with publicly available PubChem Bioassays, we trained a single QSAR model to simultaneously predict dopamine receptor D4 (DRD4) antagonist activity at multiple thresholds (10, 100, 500, 1,000, and 5,000 nM). Subsequently, we employed this QSAR model to predict the DRD4 antagonist activity of our newly created library (Figure 10B).

As might be expected, there are a high density of molecules with a low (< 0.20) local PPV for activity at 10 nM; however, as the threshold for activity increases, the density of molecules that are identified as active increases (Figure 10B). We also quantified the number of HBDs, HBAs, and rotatable bonds in our molecules (Figure 10C). Most compounds have fewer than 5 HBD and 10 rotatable bonds. Approximately half of the dataset contains 10 or more HBA, which would contribute to Lipinski’s rules violations, though many FDA-approved molecules do not follow Lipinski rules strictly (DeGoey et al., 2018). Nevertheless, number of HBAs may be one criterion by which to filter out molecules from the library from further analysis.

We also estimated topological polar surface area (TPSA) (Figure 10D) and water-octanol partition coefficient (logP) (Figure 10E). More than half of the molecules have a TPSA less than 150 Å^2^. One could also filter out molecules from the library that have TPSA greater than 150 Å^2^ and/or greater than 10 rotatable bonds (Veber rules for druglikeness). We performed logP estimates with three unique methods: 1) the DNN we trained in Section 7.4.2; 2) a property-based metric from Xing and Glen, 2002 (Xing and Glen, 2002); and 3) an atom-based metric from Mannhold et al., 2008 (Mannhold and Van de Waterbeemd, 2001). Each of these metrics are available in the BCL as molecular properties and can be employed to characterize the solubility of candidate compound libraries.

Finally, we display predicted activity at 100 nM as a function of synthetic accessibility score (SAScore) (Ertl and Schuffenhauer, 2009) (Figure 10F). Encouragingly, the molecules predicted most likely to be active at 100 nM (local PPV ≥0.80) have SAScores below 2.0, well-within an acceptable range (Ertl and Schuffenhauer, 2009). Overall, the SAScores of the library are low, reflective of the reaction type and selected reagents (Figure 10F). We selected six random molecules with local PPV greater than 0.80 at the 100 nM activity cutoff for display (Figure 10G). These molecules are topologically similar to known antagonists of DRs, specifically DRD4; however, it is possible that this reaction produces a scaffold with an activity cliff (loss of protonation of the piperazine ring) (Berry et al., 2010; Lindsley and Hopkins, 2017).

## Discussion

The BCL is an academic research project made available for public use. As an academic research project, the BCL is under continuous development. Ongoing improvements are anticipated for many of the applications described here, including small molecule conformer sampling, small molecule flexible alignment, descriptor/feature generation, and additional machine learning architectures (e.g., random forest, gradient boosting, and convolutional neural networks), strategies, and pre-generated models. In addition, several new tools are currently under active development for tasks such as library design, *de novo* drug design, pharmacophore mapping, and more.

This manuscript has focused extensively on LB in silico drug discovery tools; however, we have also begun incorporating SB tools, such as deep learning-based protein-ligand interaction scoring (Brown et al., 2021). Two primary goals moving forward are 1) continuing to increase the accessibility of the BCL to other scientists, and 2) integrating the BCL with other state-of-the-art software packages to allow for more complex protocol design. To accomplish these goals in tandem, we are completing scientific advances and software changes required to functionally integrate and compile the BCL in the Rosetta macromolecular modeling suite (Leman et al., 2020), enabling access to protocol development at the C++ (Rosetta applications), Python (PyRosetta), and XML (RosettaScripts) levels, in addition to the API described in this manuscript. We are also developing a graphical user interface (GUI) for the BCL LB drug discovery. The GUI will enable on-the-fly QSAR/QSPR calculations and druglikeness evaluation while the user is drawing molecules.

Our hope is that this manuscript will serve as a resource for those interested in utilizing the BCL for cheminformatics research. Several high level BCL applications can also be accessed via webserver for non-expert users. The webserver is available through the BCL Commons website at http://www.meilerlab.org/bclcommons. Example files mentioned throughout the manuscript are freely available on the Meiler Lab GitHub page.

The BCL can be downloaded freely from http://www.meilerlab.org/bclcommons and requires a supporting license from http://meilerlab.org/servers/bcl-academic-license that is free for academic and non-profit users, with commercial licenses available for a fee.

## Data Availability

The datasets presented in this study can be found in online repositories. The names of the repository/repositories and accession number(s) can be found below: https://github.com/Meilerlab.

## References

[B1] Acharya C. Coop A. Polli J. E. Mackerell A. D. (2011). Recent Advances in Ligand-Based Drug Design: Relevance and Utility of the Conformationally Sampled Pharmacophore Approach. Curr. Comput. Aided Drug Des. 7, 10–22. 10.2174/157340911793743547 20807187 PMC2975775

[B2] Bemis G. W. Murcko M. A. (1996). The Properties of Known Drugs. 1. Molecular Frameworks. J. Med. Chem. 39, 2887–2893. 10.1021/jm9602928 8709122

[B3] Berry C. B. Locuson C. W. Daniels J. S. Lindsley C. W. Hopkins C. R. (2010). “Discovery and Characterization of ML398, a Potent and Selective Chiral Morpholine Based Antagonist of the Dopamine 4 (D4) Receptor,” in Probe Reports from the NIH Molecular Libraries Program (Bethesda (MD): National Center for Biotechnology Information (US)). 25834901

[B4] Bharath R. Steven K. Patrick R. Dale Webster D. K. Vijay P. (2015). Massively Multitask Networks for Drug Discovery. Ithaca, NY: arXiv:1502.02072v1.

[B5] Bickerton G. R. Paolini G. V. Besnard J. Muresan S. Hopkins A. L. (2012). Quantifying the Chemical beauty of Drugs. Nat. Chem. 4, 90–98. 10.1038/nchem.1243 22270643 PMC3524573

[B6] Boström J. Norrby P. O. Liljefors T. (1998). Conformational Energy Penalties of Protein-Bound Ligands. J. Comput. Aided Mol. Des. 12, 383–396. 9777496 10.1023/a:1008007507641

[B7] Bozhanova N. G. Calcutt M. W. Beavers W. N. Brown B. P. Skaar E. P. Meiler J. (2021). Lipocalin Blc Is a Potential Heme-Binding Protein. FEBS Lett. 595, 206–219. 10.1002/1873-3468.14001 33210733 PMC8177097

[B8] Brown B. P. Mendenhall J. Geanes A. R. Meiler J. (2021). General Purpose Structure-Based Drug Discovery Neural Network Score Functions with Human-Interpretable Pharmacophore Maps. J. Chem. Inf. Model. 61, 603–620. 10.1021/acs.jcim.0c01001 33496578 PMC7903419

[B9] Brown B. P. Mendenhall J. Meiler J. (2019). BCL:MolAlign: Three-Dimensional Small Molecule Alignment for Pharmacophore Mapping. J. Chem. Inf. Model. 59, 689–701. 10.1021/acs.jcim.9b00020 30707580 PMC6598199

[B10] Brylinski M. Skolnick J. (2008). Q-dock: Low-Resolution Flexible Ligand Docking with Pocket-specific Threading Restraints. J. Comput. Chem. 29, 1574–1588. 10.1002/jcc.20917 18293308 PMC2726574

[B11] Butkiewicz M. Lowe E. W. Mueller R. Mendenhall J. L. Teixeira P. L. Weaver C. D. (2013). Benchmarking Ligand-Based Virtual High-Throughput Screening with the PubChem Database. Molecules 18, 735–756. 10.3390/molecules18010735 23299552 PMC3759399

[B12] Cappel D. Dixon S. L. Sherman W. Duan J. (2015). Exploring Conformational Search Protocols for Ligand-Based Virtual Screening and 3-D QSAR Modeling. J. Comput. Aided Mol. Des. 29, 165–182. 10.1007/s10822-014-9813-4 25408244

[B13] Chan S. L. (2017). MolAlign: an Algorithm for Aligning Multiple Small Molecules. J. Comput. Aided Mol. Des. 31, 523–546. 10.1007/s10822-017-0023-8 28573347

[B14] Combs S. A. Deluca S. L. Deluca S. H. Lemmon G. H. Nannemann D. P. Nguyen E. D. (2013). Small-molecule Ligand Docking into Comparative Models with Rosetta. Nat. Protoc. 8, 1277–1298. 10.1038/nprot.2013.074 23744289 PMC5750396

[B15] Dahl G. E. (2014). Multi-task Neural Networks for QSAR Predictions. Ithaca, NY: arXiv preprint arXiv:1406.1231.

[B16] Davis I. W. Baker D. (2009). RosettaLigand Docking with Full Ligand and Receptor Flexibility. J. Mol. Biol. 385, 381–392. 10.1016/j.jmb.2008.11.010 19041878

[B17] DeGoey D. A. Chen H. J. Cox P. B. Wendt M. D. (2018). Beyond the Rule of 5: Lessons Learned from AbbVie's Drugs and Compound Collection. J. Med. Chem. 61, 2636–2651. 10.1021/acs.jmedchem.7b00717 28926247

[B18] DeLuca S. Khar K. Meiler J. (2015). Fully Flexible Docking of Medium Sized Ligand Libraries with RosettaLigand. PLoS One 10, e0132508. 10.1371/journal.pone.0132508 26207742 PMC4514752

[B19] Ertl P. Schuffenhauer A. (2009). Estimation of Synthetic Accessibility Score of Drug-like Molecules Based on Molecular Complexity and Fragment Contributions. J. Cheminform 1, 8. 10.1186/1758-2946-1-8 20298526 PMC3225829

[B20] Ferri C. Flach P. Hernandez-Orallo J. (2002). “Learning Decision Trees Using the Area under the ROC Curve,” in Machine Learning, Proceedings of the Nineteenth International Conference (ICML 2002) (Sydney, Australia: University of New South Wales).

[B21] Friedrich N. O. de Bruyn Kops C. Flachsenberg F. Sommer K. Rarey M. Kirchmair J. (2017a). Benchmarking Commercial Conformer Ensemble Generators. J. Chem. Inf. Model. 57, 2719–2728. 10.1021/acs.jcim.7b00505 28967749

[B22] Friedrich N. O. Flachsenberg F. Meyder A. Sommer K. Kirchmair J. Rarey M. (2019). Conformator: A Novel Method for the Generation of Conformer Ensembles. J. Chem. Inf. Model. 59, 731–742. 10.1021/acs.jcim.8b00704 30747530

[B23] Friedrich N. O. Meyder A. de Bruyn Kops C. Sommer K. Flachsenberg F. Rarey M. (2017b). High-Quality Dataset of Protein-Bound Ligand Conformations and its Application to Benchmarking Conformer Ensemble Generators. J. Chem. Inf. Model. 57, 529–539. 10.1021/acs.jcim.6b00613 28206754

[B24] Friedrich N. O. Simsir M. Kirchmair J. (2018). How Diverse Are the Protein-Bound Conformations of Small-Molecule Drugs and Cofactors? Front. Chem. 6, 68. 10.3389/fchem.2018.00068 29637066 PMC5880911

[B25] Friesner R. A. Banks J. L. Murphy R. B. Halgren T. A. Klicic J. J. Mainz D. T. (2004). Glide: a New Approach for Rapid, Accurate Docking and Scoring. 1. Method and Assessment of Docking Accuracy. J. Med. Chem. 47, 1739–1749. 10.1021/jm0306430 15027865

[B26] Hanker A. B. Brown B. P. Meiler J. MarÃn A. Jayanthan H. S. Ye D. (2021). Co-occurring Gain-Of-Function Mutations in HER2 and HER3 Modulate HER2/HER3 Activation, Oncogenesis, and HER2 Inhibitor Sensitivity. Cancer Cell 39, 1099–e8. e8. 10.1016/j.ccell.2021.06.001 34171264 PMC8355076

[B27] Hartmann C. Antes I. Lengauer T. (2009). Docking and Scoring with Alternative Side-Chain Conformations. Proteins 74, 712–726. 10.1002/prot.22189 18704939

[B28] Hassan M. Brown R. D. Varma-O'brien S. Rogers D. (2006). Cheminformatics Analysis and Learning in a Data Pipelining Environment. Mol. Divers. 10, 283–299. 10.1007/s11030-006-9041-5 17031533

[B29] Hecker E. A. Duraiswami C. Andrea T. A. Diller D. J. (2002). Use of Catalyst Pharmacophore Models for Screening of Large Combinatorial Libraries. J. Chem. Inf. Comput. Sci. 42, 1204–1211. 10.1021/ci020368a 12377010

[B30] Jain A. N. (2004). Ligand-based Structural Hypotheses for Virtual Screening. J. Med. Chem. 47, 947–961. 10.1021/jm030520f 14761196

[B31] Kaufmann K. W. Lemmon G. H. Deluca S. L. Sheehan J. H. Meiler J. (2010). Practically Useful: what the Rosetta Protein Modeling Suite Can Do for You. Biochemistry 49, 2987–2998. 10.1021/bi902153g 20235548 PMC2850155

[B32] Kaufmann K. W. Meiler J. (2012). Using RosettaLigand for Small Molecule Docking into Comparative Models. PLoS One 7, e50769. 10.1371/journal.pone.0050769 23239984 PMC3519832

[B33] Kawai K. Fujishima S. Takahashi Y. (2008). Predictive Activity Profiling of Drugs by Topological-Fragment-Spectra-Based Support Vector Machines. J. Chem. Inf. Model. 48, 1152–1160. 10.1021/ci7004753 18533712

[B34] Kohonen T. (1990). The Self-Organizing Map. Proc. IEEE 78, 1464–1480. 10.1109/5.58325

[B35] Korolev D. Balakin K. V. Nikolsky Y. Kirillov E. Ivanenkov Y. A. Savchuk N. P. (2003). Modeling of Human Cytochrome P450-Mediated Drug Metabolism Using Unsupervised Machine Learning Approach. J. Med. Chem. 46, 3631–3643. 10.1021/jm030102a 12904067

[B36] Kothiwale S. Mendenhall J. L. Meiler J. (2015). BCL:Conf: Small Molecule Conformational Sampling Using a Knowledge Based Rotamer Library. J. Cheminform. 7, 47. 10.1186/s13321-015-0095-1 26473018 PMC4607025

[B37] Labute P. Williams C. Feher M. Sourial E. Schmidt J. M. (2001). Flexible Alignment of Small Molecules. J. Med. Chem. 44, 1483–1490. 10.1021/jm0002634 11334559

[B38] Le Roux N. Bengio Y. (2008). Representational Power of Restricted Boltzmann Machines and Deep Belief Networks. Neural Comput. 20, 1631–1649. 10.1162/neco.2008.04-07-510 18254699

[B39] Leman J. K. Weitzner B. D. Lewis S. M. Adolf-Bryfogle J. Alam N. Alford R. F. (2020). Macromolecular Modeling and Design in Rosetta: Recent Methods and Frameworks. Nat. Methods 17, 665–680. 10.1038/s41592-020-0848-2 32483333 PMC7603796

[B40] Lemmon G. Kaufmann K. Meiler J. (2012). Prediction of HIV-1 Protease/inhibitor Affinity Using RosettaLigand. Chem. Biol. Drug Des. 79, 888–896. 10.1111/j.1747-0285.2012.01356.x 22321894 PMC3342459

[B41] Lemmon G. Meiler J. (2012). Rosetta Ligand Docking with Flexible XML Protocols. Methods Mol. Biol. 819, 143–155. 10.1007/978-1-61779-465-0_10 22183535 PMC3749076

[B42] Lindsley C. W. Hopkins C. R. (2017). Return of D4 Dopamine Receptor Antagonists in Drug Discovery. J. Med. Chem. 60, 7233–7243. 10.1021/acs.jmedchem.7b00151 28489950

[B43] Lo Y. C. Rensi S. E. Torng W. Altman R. B. (2018). Machine Learning in Chemoinformatics and Drug Discovery. Drug Discov. Today 23, 1538–1546. 10.1016/j.drudis.2018.05.010 29750902 PMC6078794

[B44] Lowe E. W. Butkiewicz M. Spellings M. Albert O. Meiler J. (2011). Comparative Analysis of Machine Learning Techniques for the Prediction of LogP. IEEE.

[B45] Ma X. H. Wang R. Yang S. Y. Li Z. R. Xue Y. Wei Y. C. (2008). Evaluation of Virtual Screening Performance of Support Vector Machines Trained by Sparsely Distributed Active Compounds. J. Chem. Inf. Model. 48, 1227–1237. 10.1021/ci800022e 18533644

[B46] Macalino S. J. Gosu V. Hong S. Choi S. (2015). Role of Computer-Aided Drug Design in Modern Drug Discovery. Arch. Pharm. Res. 38, 1686–1701. 10.1007/s12272-015-0640-5 26208641

[B47] Mannhold R. Van de Waterbeemd H. (2001). Substructure and Whole Molecule Approaches for Calculating Log P. J. Comput. Aided Mol. Des. 15, 337–354. 10.1023/a:1011107422318 11349816

[B48] Mariusz B. Ralf M. Danilo S. Eric D. Jens M. Kai C. (2009). Application of Machine Learning Approaches on Quantitative Structure Activity Relationships. best student paper in IEEE symposium in CIBCB.

[B49] Meiler J. Baker D. (2006). ROSETTALIGAND: Protein-Small Molecule Docking with Full Side-Chain Flexibility. Proteins 65, 538–548. 10.1002/prot.21086 16972285

[B50] Mendenhall J. Meiler J. (2016). Improving Quantitative Structure-Activity Relationship Models Using Artificial Neural Networks Trained with Dropout. J. Comput. Aided Mol. Des. 30, 177–189. 10.1007/s10822-016-9895-2 26830599 PMC4798928

[B51] Mendenhall J. Brown B. P. Kothiwale S. Meiler J. (2020). BCL:Conf: Improved Open-Source Knowledge-Based Conformation Sampling Using the Crystallography Open Database. J. Chem. Inf. Model. 61, 189–201. 10.1021/acs.jcim.0c01140 33351632 PMC8130828

[B52] Mobley D. L. Guthrie J. P. (2014). FreeSolv: a Database of Experimental and Calculated Hydration Free Energies, with Input Files. J. Comput. Aided Mol. Des. 28, 711–720. 10.1007/s10822-014-9747-x 24928188 PMC4113415

[B53] Morris G. M. Huey R. Lindstrom W. Sanner M. F. Belew R. K. Goodsell D. S. (2009). AutoDock4 and AutoDockTools4: Automated Docking with Selective Receptor Flexibility. J. Comput. Chem. 30, 2785–2791. 10.1002/jcc.21256 19399780 PMC2760638

[B54] Munshi A. (2008). OpenCL: Parallel Computing on the GPU and CPU. Tutorial: SIGGRAPH.

[B55] Nicklaus M. C. Wang S. Driscoll J. S. Milne G. W. (1995). Conformational Changes of Small Molecules Binding to Proteins. Bioorg. Med. Chem. 3, 411–428. 10.1016/0968-0896(95)00031-b 8581425

[B56] Nitish S. Geoffrey H. Alex K. Ilya S. Ruslan S. (2014). Dropout: A Simple Way to Prevent Neural Networks from Overfitting. J. Machine Learn. Res. 15, 1929–1958.

[B57] Perola E. Charifson P. S. (2004). Conformational Analysis of Drug-like Molecules Bound to Proteins: an Extensive Study of Ligand Reorganization upon Binding. J. Med. Chem. 47, 2499–2510. 10.1021/jm030563w 15115393

[B58] Ramalingam S. S. Yang J. C.-H. Lee C. K. Kurata T. Kim D.-W. John T. (2018). Osimertinib as First-Line Treatment of EGFR Mutation-Positive Advanced Non-small-cell Lung Cancer. Jco 36, 841–849. 10.1200/JCO.2017.74.7576 28841389

[B59] Rogers D. Hahn M. (2010). Extended-Connectivity Fingerprints. J. Chem. Inf. Model. 50, 742–754. 10.1021/ci100050t 20426451

[B60] SciTegic (2007). Pipeline Pilot - Streamlines the Integration and Analysis of Vast Quantities of Data Flooding the Research Informatics World. Springer.

[B61] Sheridan R. P. (2012). Three Useful Dimensions for Domain Applicability in QSAR Models Using Random forest. J. Chem. Inf. Model. 52, 814–823. 10.1021/ci300004n 22385389

[B62] Sitzmann M. Weidlich I. E. Filippov I. V. Liao C. Peach M. L. Ihlenfeldt W. D. (2012). PDB Ligand Conformational Energies Calculated Quantum-Mechanically. J. Chem. Inf. Model. 52, 739–756. 10.1021/ci200595n 22303903 PMC7491702

[B63] Sliwoski G. Kothiwale S. Meiler J. Lowe E. W. (2014). Computational Methods in Drug Discovery. Pharmacol. Rev. 66, 334–395. 10.1124/pr.112.007336 24381236 PMC3880464

[B64] Sliwoski G. Mendenhall J. Meiler J. (2015). Autocorrelation Descriptor Improvements for QSAR: 2DA_Sign and 3DA_Sign. J. Comput. Aided Mol. Des. 30, 209–217. 10.1007/s10822-015-9893-9 26721261 PMC4803518

[B65] Syracuse Research Corporation (1994). Physical/Chemical Property Database. Syracuse, NY: PHYSPROP.

[B66] Tetko I. V. Sushko I. Pandey A. K. Zhu H. Tropsha A. Papa E. (2008). Critical Assessment of QSAR Models of Environmental Toxicity against tetrahymena Pyriformis: Focusing on Applicability Domain and Overfitting by Variable Selection. J. Chem. Inf. Model. 48, 1733–1746. 10.1021/ci800151m 18729318

[B67] Tijmen Tieleman (2008). “Training Restricted Boltzmann Machines Using Approximations to the Likelihood Gradient,” in Machine Learning, Proceedings of the Twenty-Fifth International Conference (ICML 2008) (Helsinki, Finland: DBLP).

[B68] Usha T. Shanmugarajan D. Goyal A. K. Kumar C. S. Middha S. K. (2017). Recent Updates on Computer-Aided Drug Discovery: Time for a Paradigm Shift. Curr. Top. Med. Chem. 17, 3296–3307. 10.2174/1568026618666180101163651 29295698

[B69] Vamathevan J. Clark D. Czodrowski P. Dunham I. Ferran E. Lee G. (2019). Applications of Machine Learning in Drug Discovery and Development. Nat. Rev. Drug Discov. 18, 463–477. 10.1038/s41573-019-0024-5 30976107 PMC6552674

[B70] Vlachakis D. Fakourelis P. Megalooikonomou V. Makris C. Kossida S. (2015). DrugOn: a Fully Integrated Pharmacophore Modeling and Structure Optimization Toolkit. PeerJ 3, e725. 10.7717/peerj.725 25648563 PMC4304849

[B71] Wang Y. H. Li Y. Yang S. L. Yang L. (2005). Classification of Substrates and Inhibitors of P-Glycoprotein Using Unsupervised Machine Learning Approach. J. Chem. Inf. Model. 45, 750–757. 10.1021/ci050041k 15921464

[B72] Wishart D. S. Feunang Y. D. Guo A. C. Lo E. J. Marcu A. Grant J. R. (2018). DrugBank 5.0: a Major Update to the DrugBank Database for 2018. Nucleic Acids Res. 46, D1074–D1082. 10.1093/nar/gkx1037 29126136 PMC5753335

[B73] Wu Z. Ramsundar B. Feinberg E. N. Gomes J. Geniesse C. Pappu A. S. (2018). MoleculeNet: a Benchmark for Molecular Machine Learning. Chem. Sci. 9, 513–530. 10.1039/c7sc02664a 29629118 PMC5868307

[B74] Xing L. Glen R. C. (2002). Novel Methods for the Prediction of logP, pK(a), and logD. J. Chem. Inf. Comput. Sci. 42, 796–805. 10.1021/ci010315d 12132880

[B75] Xu Y. Ma J. Liaw A. Sheridan R. P. Svetnik V. (2017). Demystifying Multitask Deep Neural Networks for Quantitative Structure-Activity Relationships. J. Chem. Inf. Model. 57, 2490–2504. 10.1021/acs.jcim.7b00087 28872869

[B76] Yosaatmadja Y. Silva S. Dickson J. M. Patterson A. V. Smaill J. B. Flanagan J. U. (2015). Binding Mode of the Breakthrough Inhibitor AZD9291 to Epidermal Growth Factor Receptor Revealed. J. Struct. Biol. 192, 539–544. 10.1016/j.jsb.2015.10.018 26522274

