# Supplementary material for: Introduction to the BioChemical Library (BCL): An Application-Based Open-Source Toolkit for Integrated Cheminformatics and Machine Learning in Computer-Aided Drug Discovery
Source: Front Pharmacol. 2022 Feb 21;13:833099. doi: 10.3389/fphar.2022.833099 (PMC8899505; doi:10.3389/fphar.2022.833099)
Supplement: Supplementary file 1 [file DataSheet4.docx]

# Descriptor set described in supplement for

# Mendenhall, Meiler "Advances in Machine Learning Applied to Quantitative Structural Activity Relationship Modeling"

# Unpublished 2015

# 391 columns total

# This descriptor set performed equivalently to the short-range "Minimal" variant, yet has only 1/3 of the total

# descriptors due to careful selection of the atom properties used when computing 2DA and 3DA.

Combine(

# Max # of bonds between any two atoms in the molecule

Define(BondGirth=DescriptorSum(2DAMax(steps=96,property=Atom_Identity,substitution_value=nan))),

# 1 For H, -1 for heavy atoms

Define(IsHTernary=Add(Constant(-1),Multiply(IsH,Constant(2)))),

# 1 for H-Bond donors (O or N that have bond to an H), -1 for H-Bond Acceptors (any O or N) that are not donors,

# 0 for all other atoms

Define(Atom_IsInAromaticRing=GreaterEqual(lhs=BondTypeCount(property=IsAromatic,value=1),rhs=2)),

Define(Atom_IsInAromaticRingTernary=Add(Constant(-1),Multiply(Atom_IsInAromaticRing,Constant(2)))),

# Whether an atom is at the intersection of two aromatic rings (commonly due to ring fusion, but rarely spiro too)

Define(Atom_InAromaticRingIntersection=GreaterEqual(lhs=BondTypeCount(property=IsAromatic,value=1),rhs=3)),

Define(Atom_InRingIntersection=GreaterEqual(lhs=BondTypeCount(property=IsInRing,value=1),rhs=3)),

# Scalar descriptors (1 number each)

Weight,

HbondDonor,

HbondAcceptor,

LogP,

TotalCharge,

NRotBond,

NAromaticRings,

NRings,

TopologicalPolarSurfaceArea,

Girth,

BondGirth,

MaxRingSize,

Limit(MinRingSize,max=8,min=0),

MoleculeSum(Atom_InAromaticRingIntersection),

MoleculeSum(Atom_InRingIntersection),

MoleculeStandardDeviation(Atom_Vcharge),

MoleculeStandardDeviation(Atom_SigmaCharge),

MoleculeMax(Atom_Vcharge),

MoleculeMax(Atom_SigmaCharge),

MoleculeMin(Atom_Vcharge),

MoleculeMin(Atom_SigmaCharge),

MoleculeSum(Abs(Atom_Vcharge)),

MoleculeSum(Abs(Atom_SigmaCharge)),

# Sign-aware 2DA's, out to 11 bonds (36 numbers each)

# Partial is used to exclude the bin at index 2, which corresponds to when atom property^2 is negative, which does

# not occur since all atom properties return real numbers

Template(

signature=2DASign11(X),

Partial(

2DASign(property=X,steps=11),

indices(0,1,3,4,5,6,7,8,9,10,11,12,13,14,15,16,17,18,19,20,21,22,23,24,25,26,27,28,29,30,31,32)

)

),

# Sign-aware 3DA's, out to 6A, beyond which rotomer-dependent effects begin to play a significant role

# The partial is used here to remove the first 1A of data, which is always redundant because the 0A bin is

# identical to the 2DA case, and the remaining bins 0.25, 0.5, and 0.75 are generally 0

Template(

signature=3DASign24(X),

Partial(

3daSmoothSign(property=X,step size=0.25,temperature=100,steps=24,gaussian=False,interpolate=True),

indices(

12,13,14,15,16,17,18,19,20,21,22,23,24,25,26,27,28,29,30,31,32,33,34,35,36,

37,38,39,40,41,42,43,44,45,46,47,48,49,50,51,52,53,54,55,56,57,58,59,60,61,

62,63,64,65,66,67,68,69,70,71

)

)

),

2DASign11(Atom_SigmaCharge),

2DASign11(Atom_Vcharge),

2DASign11(IsHTernary),

2DASign11(Atom_IsInAromaticRingTernary),

3DASign24(Atom_SigmaCharge),

3DASign24(Atom_Vcharge),

3DASign24(IsHTernary),

3DASign24(Atom_IsInAromaticRingTernary)

)
