# Supplementary material for: Introduction to the BioChemical Library (BCL): An Application-Based Open-Source Toolkit for Integrated Cheminformatics and Machine Learning in Computer-Aided Drug Discovery
Source: Front Pharmacol. 2022 Feb 21;13:833099. doi: 10.3389/fphar.2022.833099 (PMC8899505; doi:10.3389/fphar.2022.833099)
Supplement: Supplementary file 2 [file DataSheet3.docx]

# "Short-Range" descriptor set used in

# Mendenhall, Meiler "Advances in Machine Learning Applied to Quantitative Structural Activity Relationship Modeling"

# Unpublished 2015

# 1315 numbers total, 959 non-redundant

# This descriptor set performed the best of those in the benchmark

Combine(

# Max # of bonds between any two atoms in the molecule

Define(BondGirth=DescriptorSum(2DAMax(steps=96,property=Atom_Identity,substitution_value=nan))),

# 1 For H, -1 for heavy atoms

Define(IsHTernary=Add(Constant(-1),Multiply(IsH,Constant(2)))),

# Atom polarizability made positive for H, negative for heavy atoms

Define(Atom_SignedPolarizability=Multiply(Atom_EffectivePolarizability,IsHTernary)),

# The Limit descriptor is used below on various charge descriptors to mitigate the effect of unwanted and often

# incorrect assignment of formally charged atoms in molecules.

Define(Atom_VchargeL=Limit(Atom_Vcharge,max=0.5,min=-0.5)),

Define(Atom_SigmaChargeL=Limit(Atom_SigmaCharge,max=0.25,min=-0.25)),

# Proton-charges. These are generally always positive

Define(Atom_HSigmaChargeL=Multiply(Atom_SigmaChargeL,IsH)),

Define(Atom_HVchargeL=Multiply(Atom_VchargeL,IsH)),

# Charges on Heavy atoms

Define(Atom_HeavySigmaChargeL=Multiply(Atom_SigmaChargeL,IsNotH)),

Define(Atom_HeavyVchargeL=Multiply(Atom_VchargeL,IsNotH)),

# 1 for H-Bond donors (O or N that have bond to an H), -1 for H-Bond Acceptors (any O or N) that are not donors,

# 0 for all other atoms

Define(Atom_TernaryHBond=Subtract(lhs=Atom_HbondAcceptors,rhs=Multiply(Constant(2),Atom_HbondDonors))),

Define(Atom_IsInAromaticRing=GreaterEqual(lhs=BondTypeCount(property=IsAromatic,value=1),rhs=2)),

# Whether an atom is at the intersection of two aromatic rings (commonly due to ring fusion, but rarely spiro too)

Define(Atom_InAromaticRingIntersection=GreaterEqual(lhs=BondTypeCount(property=IsAromatic,value=1),rhs=3)),

Define(Atom_InRingIntersection=GreaterEqual(lhs=BondTypeCount(property=IsInRing,value=1),rhs=3)),

# Scalar descriptors (1 number each)

Weight,

HbondDonor,

HbondAcceptor,

LogP,

TotalCharge,

NRotBond,

NAromaticRings,

NRings,

TopologicalPolarSurfaceArea,

Girth,

BondGirth,

MaxRingSize,

Limit(MinRingSize,max=8,min=0),

MoleculeSum(Atom_IsInAromaticRing),

MoleculeSum(Atom_InAromaticRingIntersection),

MoleculeSum(Atom_InRingIntersection),

MoleculeStandardDeviation(Atom_VchargeL),

MoleculeStandardDeviation(Atom_SigmaChargeL),

MoleculeMax(Atom_VchargeL),

MoleculeMax(Atom_SigmaChargeL),

MoleculeMin(Atom_VchargeL),

MoleculeMin(Atom_SigmaChargeL),

MoleculeSum(Abs(Atom_VchargeL)),

MoleculeSum(Abs(Atom_SigmaChargeL)),

# Define unsigned atom properties used in this descriptor object

Define(

AtomUnsignedProperties=Combine(

Atom_HSigmaChargeL,

Atom_HVchargeL,

Atom_IsInAromaticRing,

Atom_InAromaticRingIntersection

)

),

# Define signed atom properties used in this descriptor object

Define(

AtomSignedProperties=Combine(

Atom_SignedPolarizability,

Atom_SigmaChargeL,

Atom_HeavySigmaChargeL,

Atom_HeavyVchargeL,

Atom_VchargeL,

Atom_TernaryHBond

)

),

# Unsigned 2DAs, out to 11 bonds distant (12 numbers each)

ForEach(

template=2DA(steps=11,property=AtomProperty,normalized=False),

variable=AtomProperty,

descriptors(Atom_Identity,AtomUnsignedProperties)

),

# Unsigned 3DAs, out to 6A (24 numbers each)

ForEach(

template=3daSmooth(property=AtomProperty,step size=0.25,temperature=100,steps=24,gaussian=True,interpolate=1),

variable=AtomProperty,

descriptors(AtomUnsignedProperties)

),

# Unsigned 3DAMax's, out to 6A (24 numbers each)

ForEach(

template=3daSoftMax(property=AtomProperty,step size=0.25,temperature=100,steps=24,gaussian=False),

variable=AtomProperty,

descriptors(AtomUnsignedProperties)

),

# Sign-aware 2DA Max's, out to 5 bonds (18 numbers each)

ForEach(

template=2DAMaxSign(property=AtomProperty,steps=5),

variable=AtomProperty,

descriptors(AtomSignedProperties)

),

# Sign-aware 2DA's, out to 5 bonds (18 numbers each)

ForEach(

template=2DASign(property=AtomProperty,steps=5),

variable=AtomProperty,

descriptors(AtomSignedProperties)

),

# Sign-aware, gaussian smoothed 3DA's, out to 6A (72 numbers each)

ForEach(

template=3daSmoothSign(property=AtomProperty,step size=0.25,temperature=100,steps=24,gaussian=True,interpolate=True),

variable=AtomProperty,

descriptors(AtomSignedProperties)

),

# Sign-aware 3DA Max's, out to 6A (72 numbers each)

ForEach(

template=3daSoftMaxSign(property=AtomProperty,step size=0.25,temperature=100,steps=24,gaussian=False),

variable=AtomProperty,

descriptors(AtomSignedProperties)

)

)
