# Supplementary material for: Introduction to the BioChemical Library (BCL): An Application-Based Open-Source Toolkit for Integrated Cheminformatics and Machine Learning in Computer-Aided Drug Discovery
Source: Front Pharmacol. 2022 Feb 21;13:833099. doi: 10.3389/fphar.2022.833099 (PMC8899505; doi:10.3389/fphar.2022.833099)
Supplement: Supplementary file 3 [file DataSheet2.docx]

***BCL v4.2.0 descriptors basic***

=crt=bcl::random=> Seed was set to 5489!

=============================================================================================================================================================================================

molecule:Properties Help

BCL v4.2.0, r0, compiled on Mon Jan 17 03:05:27 2022

Usage: bcl.exe molecule:Properties [OPTIONAL FLAGS] [@FILENAMES]

=============================================================================================================================================================================================

BCL FLAGS: affect general BCL functionality, but may not be relevant for all applications

-message_level : adjust the MessageLevel

<level> minimum level of messages that will be printed, default: "Standard", Allowed values: {Error, Silent, Critical, Standard, Verbose, Debug}

<verbosity> set to Detail to print the source file and line of origination for each message, default: "Summary", Allowed values: {Summary, Detail}

-logger : change the logger this executable uses

default: "Default", Choose from the following: { Default, File}

<identifier> define a logger identifier - for file, it is the filename to be opened, optional

-help : output user help for the bcl or an application

-readme : output readme information for the application

-file_compressed_alternatives : set to enable the search for files that cannot be opened, but for which a file with a compression extension might exist as alternative

-file_compression : the type of file compression to be used for files

<compression_type> compression algorithm to be used, default: "Uncompressed", Choose from the following: { Uncompressed, BZ2, GZ, Crypt}

-enums_files : files for enum data that adds enums or overrides data of existing enum data, This flag can be followed by at most 27

<enum_file> file that is similar to a written Enums derived class

-random_seed : adjust the random seed; if flag is used, system time is used as seed, if additional parameter is passed, the given number will be used, otherwise default will be used

<seed> random seed for the random number generator, default: "5489", range: [0,18446744073709551615]

-model_path : change path for reading and writing models

<model_path_name> relative or absolute model path, default: "/dors/meilerlab/apps/bcl/model/rev_5051/", any directory, if not provided, defaults to

/dors/meilerlab/apps/bcl/model/rev_5051/; search path is: {model/:../model/:../model/:bcl/model/:/dors/meilerlab/apps/bcl/model/rev_5051/:model/:model/}

-histogram_path : change path for reading and writing histograms for scores

<path> relative or absolute path, default: "/dors/meilerlab/apps/bcl/histograms/rev_5265/", any directory, if not provided, defaults to /dors/meilerlab/apps/bcl/histograms/rev_5265/;

search path is: {histogram/:../histogram/:../histogram/:bcl/histogram/:/dors/meilerlab/apps/bcl/histograms/rev_5265/:histogram/:histogram/}

-opencl : choice of opencl platform and device type

<platform> opencl platform; Disabled because this machine lacks libOpenCL or does not have the appropriate /etc/OpenCL/vendors .icd files, or Disable was already given,

default: "Disable", allowed values: {Disable}

-scheduler : choice of scheduler and number of cpus

<scheduler> type of scheduler, default: "Serial", Choose from the following: { Serial, PThread}

<number_cpus> number of cpus for a multi job scheduler, default: "1", range: [1,1000]

=============================================================================================================================================================================================

APPLICATION FLAGS: syntax: -flagname [flagparameter1] ...

-help : output user help for the bcl or an application

-readme : output readme information for the application

-input_filenames : input files containing molecules in sdf format, This flag can be followed by any number of

<filenames> files containing molecules in sdf format, any existent file

-input_start : index (0-offset) of first molecule to load for input

<start> index (0-offset) of first molecule to load, default: "0", range: [0,18446744073709551615]

-input_max : Specify the maximum number of molecules to be loaded for input

<number> number of molecules, default: "18446744073709551615", range: [1,18446744073709551615]

-add_h : add hydrogens to molecules when loaded

-remove_h : remove hydrogens from molecules when loaded

-neutralize : neutralize charges; by default, if the flag is specified but no neutralization type is given, BondOrderAndpH will be used, otherwise, no neutralization is used. All

neutralization algorithms preserve aromaticity except BondOrderAndpHAromaticityLossOk

<method> method used to neutralize charged atoms in the molecule, default: "BondOrderAndpH", Allowed values: {None, BondOrder, pH, BondOrderAndpH, BondOrderAndpHAromaticityLossOk}

-explicit_aromaticity : write MDL bonds with aromatic bonds specified explicitly (as 4); alternatively, the default behavior during MDL writing is to kekulize aromatic rings (write

alternating single (1) / double (2) bonds)

-numeric_histogram : properties containing numeric values, min value, bin size, # bins, This flag can be followed by any number of

<property> atom or small molecule property, choose any Molecule / Atom Numeric descriptor :

*********************************************************************************************************************************************************************************

************************************************************************** Descriptors of Molecules *****************************************************************************

*********************************************************************************************************************************************************************************

Basic Implementations

* Atom_Identity : 1, for any atom or molecule. The Atom_ prefix is purely for backwards-compatibility. In new descriptor files, the use of Constant(1) is preferred. Alias for

: Constant(1)

* CovalentSurfaceArea : covalent surface area. Alias for : MoleculeSum(Atom_CovalentSurfaceArea)

* CovalentVolume : covalent volume. Alias for : MoleculeSum(Atom_CovalentVolume)

* EstCovSurfaceArea : Estimated covalent surface area (conformation independent). Alias for : MoleculeSum(Atom_EstCovalentSurfaceArea)

* EstVdwSurfaceArea : Estimated van-der-waals surface area (conformation independent), using element-based VDW radii. Alias for : MoleculeSum(Atom_EstVdwSurfaceArea)

* EstVdwSurfaceAreaCSD : Estimated van-der-waals surface area (conformation independent), using more accurate, CSD-derived atom-type VdW radii, which tend to give larger SAs

to H than EstVdwSurfaceArea. Alias for : MoleculeSum(Atom_EstVdwSurfaceAreaCSD)

* Girth : calculates the girth of a molecule

* HbondAcceptor : # hydrogen bond acceptors. Alias for : MoleculeSum(Atom_HbondAcceptors)

* HbondDonor : # hydrogen bond donors. Alias for : MoleculeSum(Atom_HbondDonors)

* LipinskiDruglike : Returns 1 if the number of Lipinski violations is less than 2. Alias for : Less(lhs=LipinskiViolations,rhs=Constant(2))

* LipinskiViolations : calculates how many times a molecule violates Lipinski's Rule of 5

* LipinskiViolationsVeber : calculates how many times a molecule violates Veber's variant of the Lipinski's Rule of 5 (<10 rotatable bonds, Polar SA < 140 A^2; see J. Med.

Chem., 2002, 45 (12), pp 2615–2623)

* LogP : Calculates logp using the heuristic formula from http://pubs.acs.org/doi/full/10.1021/ci010315d

* LogP2008 : Calculates logp using the heuristic formula from http://pubs.acs.org/doi/full/10.1021/ci010315d

* MACCS : Retrieves the MACCS of each molecule using a modified pubchem MACCS fingerprint. See ftp://ftp.ncbi.nlm.nih.gov/pubchem/specifications/pubchem_fingerprints.txt for

details on the pubchem values

* MaxRingSize : Largest unbridged ring inside the molecule; returns 2 for ringless molecules. Alias for : MoleculeMax(AtomMaxRingSize)

* MinRingSize : Smallest ring inside the molecule; returns 10000 for ringless molecules. Alias for : MoleculeMin(AtomMinRingSize)

* MoleculeComplexity : calculates the complexity of a molecule (see http://www.jcheminf.com/content/1/1/8)

* MoleculeOneFourClashScore : Calculates the sum of one-four clash scores in the molecule

* MoleculeTotalBondEnergy : Calculates the sum of bond energies derived from the statistical bond potential

* MoleculeVDWScore : Calculates the total molecule VDW score normalized by the number of atoms in the molecule

* NAromaticMacrocyclicRings : Number of Aromatic macrocyclic (>8 atoms) rings in the molecule

* NAromaticRings : Number of Aromatic rings in the molecule

* NAtoms : Number of atoms. Alias for : NElements

* NConjugatedMacrocyclicRings : Number of Conjugated macrocyclic (>8 atoms) rings in the molecule

* NConjugatedRings : Number of Conjugated rings in the molecule

* NElements : The number of Atom in the Molecule

* NHeavyAtoms : Number of non-hydrogen atoms in the molecule. Alias for : MoleculeSum(IsNotH)

* NMacrocyclicRings : Number of macrocyclic (>8 atoms) rings in the molecule

* NNonconjugatedMacrocyclicRings : Number of Nonconjugated macrocyclic (>8 atoms) rings in the molecule

* NNonconjugatedRings : Number of Nonconjugated rings in the molecule

* NRings : Number of rings in the molecule

* NRotBond : calculates the number of non-trivially rotatable bonds

* NRotBondSym : calculates the number of non-trivially rotatable bonds

* NStereo : Number of stereocenters. Alias for : MoleculeSum(NotEqual(Atom_Stereocenters,Constant(0)))

* Polarizability : total polarizability. Alias for : MoleculeSum(Atom_Polarizability)

* TopologicalPolarSurfaceArea : topological polar surface area. Alias for : MoleculeSum(Atom_TopologicalPolarSurfaceArea)

* TotalCharge : Sum of sigma and pi charges. Alias for : MoleculeSum(Atom_TotalCharge)

* TotalFormalCharge : Sum of formal charges on the molecule. Alias for : MoleculeSum(Atom_FormalCharge)

* VdwSurfaceArea : van der waals surface area. Alias for : MoleculeSum(Atom_VDWSurfaceArea)

* VdwVolume : van der waals volume. Alias for : MoleculeSum(Atom_VDWVolume)

* Weight : Molecular weight (amu). Alias for : MoleculeSum(Atom_Mass)

* XLogP : Calculates LogP using a multi-tasking deep neural network trained to predict LogP, LogS, and hydration free energy

Customizable Implementations

* 3DInterHBondCode : Represents hydrogen bond interactions in relative distance and angle correlation bins; "3DInterHBondCode(help)" shows internal options

* AffinityNet : Calculates binding affinity (in units of pKd); "AffinityNet(help)" shows internal options

* AffinityNetAD : Calculates binding affinity (in units of pKd) weighted by 1.0 minus the model applicability domain score; "AffinityNetAD(help)" shows internal options

* BuserMetric : calculates the buser similarity score between ; "BuserMetric(help)" shows internal options

* Constant : Returns a constant set of values; "Constant(help)" shows internal options

* Default : given a number, returns that number, otherwise retrieves the value of the misc property by that name; "Default(help)" shows internal options

* Define : Define an alias for a given descriptor; "Define(help)" shows internal options

* DockANNScore : Calculates a docking score based on the local ppv classifying a pose as within 1.0 Angstroms of the native pose weighted by the predicted affinity of the pose

;"DockANNScore(help)" shows internal options

* EntropyQHA : Sampling options for relative conformational entropy estimates. Descriptor output indices correspond to the global_s, local_s, global_s - local_s, and

-ln(local_s/global_s). The first 4 indices are actual entropy estimates in J*K^-1*mol^-1, while the latter 4 indices are the PCA eigenvalue sums.; "EntropyQHA(help)" shows

internal options

* IsConstitutionDruglike : Returns 1 if the molecule is druglike and 0 otherwise; "IsConstitutionDruglike(help)" shows internal options

* IsConstitutionDruglikeAndHitlike : Returns 1 if the molecule is druglike and 0 otherwise; "IsConstitutionDruglikeAndHitlike(help)" shows internal options

* MiscProperty : retrieves a molecule property that cannot be calculated directly by the bcl, e.g. biological data; "MiscProperty(help)" shows internal options

* MolAlignPharmScore : Performs quick property-based molecular alignment with BCL::MolAlign and returnsthe RMSDX score as well as atomic overlap metrics.;

"MolAlignPharmScore(help)" shows internal options

* MoleculeAtomEnvironmentMap : Determines if molecule fragment radial atom environments exist in database; "MoleculeAtomEnvironmentMap(help)" shows internal options

* MoleculeSimilarity : Searches for specified substructures within the query molecule. Returns 1 if a structure exists in the query molecule;

"MoleculeSimilarity(help)" shows internal options

* NAromaticRingHalogensMaxFragment : Determines the sum of the number of halogen substituents on aromatic rings in a molecule. Alternatively, identifies the aromatic ring with

the most number of halogens and returns that count.; "NAromaticRingHalogensMaxFragment(help)" shows internal options

* NAromaticRingHalogensTotal : Determines the sum of the number of halogen substituents on aromatic rings in a molecule. Alternatively, identifies the aromatic ring with the

most number of halogens and returns that count.; "NAromaticRingHalogensTotal(help)" shows internal options

* PCC-AD-DNN : Calculates binding affinity (in units of pKd) weighted by 1.0 minus the model applicability domain score; "PCC-AD-DNN(help)" shows internal options

* PCC-DNN : Calculates binding affinity (in units of pKd); "PCC-DNN(help)" shows internal options

* ReactionStructureSearch : Searches for specified substructures within the query molecule. Returns 1 if a structure exists in the query molecule;

"ReactionStructureSearch(help)" shows internal options

* StructureCount : Searches for specified substructures within the query molecule. Returns the number of substructure matches that exist in the query molecule;

"StructureCount(help)" shows internal options

* StructureSearch : Searches for specified substructures within the query molecule. Returns 1 if a structure exists in the query molecule;

"StructureSearch(help)" shows internal options

* Template : Create a descriptor template; which is a partial definition for a descriptor, with remaining user-defined arguments that can be set later;

"Template(help)" shows internal options

* UMol2D : calculates the number of certain types of atom environments; "UMol2D(help)" shows internal options

* UniformRandom : Returns a uniform-randomly chosen set of values; "UniformRandom(help)" shows internal options

* UniformRandomDuplicated : Returns a uniform-randomly chosen value, copied {size} times; "UniformRandomDuplicated(help)" shows internal options

Operations

* MoleculeMax : Returns the max of the given descriptor across a Molecule; "MoleculeMax(help)" shows internal options

* MoleculeMean : Returns the mean of the given descriptor across a Molecule; "MoleculeMean(help)" shows internal options

* MoleculeMin : Returns the min of the given descriptor across a Molecule; "MoleculeMin(help)" shows internal options

* MoleculeRange : Returns the range of the given descriptor across a Molecule; "MoleculeRange(help)" shows internal options

* MoleculeStandardDeviation : Returns the standarddeviation of the given descriptor across a Molecule; "MoleculeStandardDeviation(help)" shows internal options

* MoleculeSum : Returns the sum of the given descriptor across a Molecule; "MoleculeSum(help)" shows internal options

* MoleculeWeightedMean : mean of a descriptor across the Molecule, weighted by any other descriptor; "MoleculeWeightedMean(help)" shows internal options

* MoleculeWeightedStandardDeviation : standarddeviation of a descriptor across the Molecule, weighted by any other descriptor;

"MoleculeWeightedStandardDeviation(help)" shows internal options

* MoleculeWeightedSum : sum of a descriptor across the Molecule, weighted by any other descriptor; "MoleculeWeightedSum(help)" shows internal options

* ShapeMoments : computes the mean, std, and skew of atomic distances from four anchor points: the molecular centroid (mct), closest atom to the mct (cam), furthest atom from

mct (fam), and furthest atom from fam (faf). The molecular centroid is the center of the positions, weighted by the anchor property.; "ShapeMoments(help)" shows internal options

Customizable Operations

* 2DAMax : computes max for each bin of the 2DA of a specified atom property; "2DAMax(help)" shows internal options

* 2DAMaxSign : computes 2D (bond distance) autocorrelation of a specified atom property; "2DAMaxSign(help)" shows internal options

* 2DAMin : computes min for each bin of the 2DA of a specified atom property; "2DAMin(help)" shows internal options

* 3DAClosestPairRealSpace : Relates the autocorrelations of two independent molecules; "3DAClosestPairRealSpace(help)" shows internal options

* 3DAClosestPairRealSpaceAsymmetry : Relates the autocorrelations of two independent molecules; "3DAClosestPairRealSpaceAsymmetry(help)" shows internal options

* 3DAPairConvolution : Relates the autocorrelations of two independent molecules; "3DAPairConvolution(help)" shows internal options

* 3DAPairConvolutionAsymmetry : Relates the autocorrelations of two independent molecules; "3DAPairConvolutionAsymmetry(help)" shows internal options

* 3DAPairRealSpace : Relates the autocorrelations of two independent molecules; "3DAPairRealSpace(help)" shows internal options

* 3DAPairRealSpaceAsymmetry : Relates the autocorrelations of two independent molecules; "3DAPairRealSpaceAsymmetry(help)" shows internal options

* 3DAPairRealSpaceConvolution : Relates the autocorrelations of two independent molecules; "3DAPairRealSpaceConvolution(help)" shows internal options

* 3DAPairRealSpaceConvolutionAsymmetry : Relates the autocorrelations of two independent molecules; "3DAPairRealSpaceConvolutionAsymmetry(help)" shows internal options

* 3dDistribution : computes the smooth radial distribution function using a given atom property; "3dDistribution(help)" shows internal options

* 3dDistributionSign : computes the smooth radial distribution function using a given atom property; "3dDistributionSign(help)" shows internal options

* 3daSoftMax : computes the smooth radial distribution function using a given atom property; "3daSoftMax(help)" shows internal options

* 3daSoftMaxSign : computes the smooth radial distribution function using a given atom property; "3daSoftMaxSign(help)" shows internal options

* 3daSoftMin : computes the smooth radial distribution function using a given atom property; "3daSoftMin(help)" shows internal options

* Central2DASign : Measure 2DASign of property at variable distances from molecule topological center; "Central2DASign(help)" shows internal options

* CoulombicForce : computes the intramolecular coulombic force (without the dielectric constant); "CoulombicForce(help)" shows internal options

* MolecularAsymmetry : Calculates an rdf-like asymmetry vector for the molecule based on overall distribution of atoms and/or properties. Use of this descriptor must be cited

as: Sliwoski, Gregory, et al. "BCL:: EMAS—Enantioselective Molecular Asymmetry Descriptor for 3D-QSAR." Molecules 17.8 (2012): 9971-9989.

Link: www.http://meilerlab.org/index.php/publications/show/2012

; "MolecularAsymmetry(help)" shows internal options

* MolecularFragmentRescale : Statistics of a descriptor across the series of fragments split off the original molecule; "MolecularFragmentRescale(help)" shows internal options

* MolecularFragmentStatistics : Statistics of a descriptor across the series of fragments split off the original molecule;

"MolecularFragmentStatistics(help)" shows internal options

* MolecularMaxFragmentStatistics : Statistics of a descriptor across the series of fragments split off the original molecule;

"MolecularMaxFragmentStatistics(help)" shows internal options

* MoleculeHistogram1D : computes a histogram using a single descriptor; "MoleculeHistogram1D(help)" shows internal options

* RDFMaxSign : computes the smooth radial distribution function using a given atom property; "RDFMaxSign(help)" shows internal options

* RotamerCoulombicForce : computes the intramolecular coulombic force (without the dielectric constant) due to the rotamer chosen, thus ignoring covalent and 1,3 bonding

interactions; "RotamerCoulombicForce(help)" shows internal options

* Triangulator : computes triangular autocorrelation of a specified atom property.This is much like RDF, but considers all triplets of atoms.;

"Triangulator(help)" shows internal options

*********************************************************************************************************************************************************************************

**************************************************************************** Descriptors of Atoms *******************************************************************************

*********************************************************************************************************************************************************************************

These can be converted into a Molecule-level descriptor using MoleculeSum(X), where X is any descriptor listed below

Basic Implementations

* AtomMaxRingSize : Maximum size of a ring that this atom is part of. For atoms that are not in a ring, returns 2. The size of the largest ring with no internal rings is

returned for atoms that are part of ring systems

* AtomMinRingSize : Minimum size of a ring that this atom is part of. For atoms that are not in a ring, returns 10000.

* AtomTypeNumber : Retrieves the atomic number of each atom

* Atom_AdditiveAtomicPolarizability : Retrieves the AdditiveAtomicPolarizability of desired atom

* Atom_AromaticityAxes : For aromatic atoms, the axes of the aromatic field

* Atom_BoilingPoint : Retrieves the elemental BoilingPoint for each atom

* Atom_CovalentRadius : Retrieves the elemental CovalentRadius for each atom

* Atom_CovalentSurfaceArea : approximates the surface area of atoms using the covalent radius, considering overlap from neighboring atoms. Alias for :

Atom_SurfaceArea(radius=Atom_CovalentRadius,min radius=Molecule/Atom Numeric Descriptor)

* Atom_CovalentVolume : approximates the volume of atoms using the covalent radius, considering overlap from neighboring atoms. Alias for :

Atom_Volume(radius=Atom_CovalentRadius,min radius=Molecule/Atom Numeric Descriptor)

* Atom_EffectivePolarizability : atomic polarizability smoothed over molecule

* Atom_ElectroNegativity : Retrieves the elemental ElectroNegativity for each atom

* Atom_EstCovalentSurfaceArea : The surface area, estimated from atom type covalent radii

* Atom_EstVdwSurfaceArea : The surface area, estimated from atom type elemental van der waals radii

* Atom_EstVdwSurfaceAreaCSD : The surface area, estimated from atom type CSD-derived van der waals radii

* Atom_FormalCharge : the formal charge of each atom

* Atom_GyromagneticRatio : Retrieves the elemental GyromagneticRatio for each atom

* Atom_HbondAcceptors : 1 for hydrogen bond acceptors (N and O), 0 for other elements

* Atom_HbondDonors : 1 for hydrogen bond donors (NH and OH), 0 for others

* Atom_IonizationPotential : Retrieves the elemental IonizationPotential for each atom

* Atom_IsSP : 1 for sp, -1 for non-sp non-terminal atoms, 0 for everything else

* Atom_IsSP2 : 1 for sp2, -1 for non-sp2 non-terminal atoms, 0 for everything else

* Atom_IsSP3 : 1 for sp3, -1 for non-sp3 non-terminal atoms, 0 for everything else

* Atom_LonePairEN : calculates lone-pair electronegativity, see http://www.springerlink.com/content/w300520690302287/fulltext.pdf

* Atom_LonePairElectronAffinity : Retrieves the LonePairElectronAffinity of desired atom

* Atom_LonePairElectronegativity : Retrieves the LonePairElectronegativity of desired atom

* Atom_LonePairIonizationPotential : Retrieves the LonePairIonizationPotential of desired atom

* Atom_MainGroup : Retrieves the elemental MainGroup for each atom

* Atom_Mass : Retrieves the elemental Mass for each atom

* Atom_MeltingPoint : Retrieves the elemental MeltingPoint for each atom

* Atom_NeighborDirection : The unit vector formed by the relative positions of neighboring atoms

* Atom_NumberValences : the formal charge of each atom

* Atom_PiCharge : uses Hueckel matrix to determine pi-orbital partial charge

* Atom_PiEN : uses Hueckel matrix to determine pi-orbital electronegativity

* Atom_PiOrbitalElectronegativityMulliken : Retrieves the PiOrbitalElectronegativityMulliken of desired atom

* Atom_PiOrbitalElectronegativityPauling : Retrieves the PiOrbitalElectronegativityPauling of desired atom

* Atom_PiValenceStateElectronAffinity : Retrieves the PiValenceStateElectronAffinity of desired atom

* Atom_PiValenceStateIonizationPotential : Retrieves the PiValenceStateIonizationPotential of desired atom

* Atom_Polarizability : computes the polarizability of each atom using the method from see J.Am.Chem.Soc. Vol 112, No. 23, 1990, 8534

* Atom_SigmaCharge : uses PEOE to determine sigma-orbital partial charge

* Atom_SigmaEN : uses PEOE to determine sigma-orbital electronegativity

* Atom_SigmaOrbitalElectronegativityMulliken : Retrieves the SigmaOrbitalElectronegativityMulliken of desired atom

* Atom_SigmaOrbitalElectronegativityPauling : Retrieves the SigmaOrbitalElectronegativityPauling of desired atom

* Atom_SigmaValenceStateElectronAffinity : Retrieves the SigmaValenceStateElectronAffinity of desired atom

* Atom_SigmaValenceStateIonizationPotential : Retrieves the SigmaValenceStateIonizationPotential of desired atom

* Atom_Stereocenters : 1 for R, -1 for S, 0 for achiral atoms, 2 for undefined chirality

* Atom_TopologicalPolarSurfaceArea : see Ertl, et. al. J. Med. Chem. 2000, 43, 3715

* Atom_TotalCharge : Returns the total charge on an atom. Alias for : Add(Atom_SigmaCharge,Atom_PiCharge)

* Atom_VDWSurfaceArea : approximates the surface area of atoms using the vdw radius, considering overlap from neighboring atoms. Alias for :

Atom_SurfaceArea(radius=Atom_VDWaalsRadius,min radius=Atom_CovalentRadius)

* Atom_VDWVolume : approximates the volume of atoms using the vdw radius, considering overlap from neighboring atoms. Alias for : Atom_Volume(radius=Atom_VDWaalsRadius,min

radius=Atom_CovalentRadius)

* Atom_VDWaalsRadius : Retrieves the elemental VDWaalsRadius for each atom

* Atom_Vcharge : Partial charges computed using vcharge 2003 algorithm and parameters, see http://pubs.acs.org/doi/full/10.1021/ci034148o

* Atom_VchargeV2 : Partial charges computed using vcharge 2003 algorithm and parameters, see http://pubs.acs.org/doi/full/10.1021/ci034148o

* AtomicNumbers : Retrieves the atomic number of each atom

* IsB : Returns 1 for boron atoms, 0 for others. Alias for : Equal(AtomicNumbers,Constant(5))

* IsBr : Returns 1 for bromine atoms, 0 for others. Alias for : Equal(AtomicNumbers,Constant(35))

* IsC : Returns 1 for carbon atoms, 0 for others. Alias for : Equal(AtomicNumbers,Constant(6))

* IsCl : Returns 1 for chlorine atoms, 0 for others. Alias for : Equal(AtomicNumbers,Constant(17))

* IsF : Returns 1 for fluorine atoms, 0 for others. Alias for : Equal(AtomicNumbers,Constant(9))

* IsH : Returns 1 for hydrogen atoms, 0 for heavy atoms. Alias for : Less(lhs=AtomicNumbers,rhs=Constant(1.5))

* IsHalogen : Returns 1 for atoms in main group 7 (F,Cl,Br,I,At,Uus). Alias for : Equal(Atom_MainGroup,Constant(7))

* IsI : Returns 1 for iodine atoms, 0 for others. Alias for : Equal(AtomicNumbers,Constant(53))

* IsN : Returns 1 for nitrogen atoms, 0 for others. Alias for : Equal(AtomicNumbers,Constant(7))

* IsNotC : Returns 1 for non-carbon atoms, 0 for others. Alias for : NotEqual(AtomicNumbers,Constant(6))

* IsNotH : Returns 1 for heavy atoms, 0 for hydrogen atoms. Alias for : Greater(lhs=AtomicNumbers,rhs=Constant(1.5))

* IsO : Returns 1 for oxygen atoms, 0 for others. Alias for : Equal(AtomicNumbers,Constant(8))

* IsP : Returns 1 for phosphorus atoms, 0 for others. Alias for : Equal(AtomicNumbers,Constant(15))

* IsPeriodThreePlus : Returns 1 for all elements in periods 3-7. Alias for : Greater(lhs=AtomicNumbers,rhs=Constant(10.5))

* IsS : Returns 1 for sulfur atoms, 0 for others. Alias for : Equal(AtomicNumbers,Constant(16))

* IsSi : Returns 1 for silicon atoms, 0 for others. Alias for : Equal(AtomicNumbers,Constant(14))

* Position : Returns the X,Y,Z coordinates of the Atom

Customizable Implementations

* Atom_RelativePropertyScore : Compute per-atom contribution of QSAR score relative to a provided scaffold; "Atom_RelativePropertyScore(help)" shows internal options

* BondTypeCount : Counts the number of bonds that satisfy a condition (property == value); "BondTypeCount(help)" shows internal options

Operations

* Atom_SurfaceArea : approximates the surface area of the atom, considering neighbor overlap; "Atom_SurfaceArea(help)" shows internal options

* Atom_Volume : approximates the volume of the atom, considering neighbor overlap; "Atom_Volume(help)" shows internal options

Customizable Operations

* 3DA : computes 3D autocorrelation of a specified atom property; "3DA(help)" shows internal options

* ElementHistogram1D : computes a histogram using a single descriptor; "ElementHistogram1D(help)" shows internal options

* ElementHistogram2D : computes a binary histogram using different descriptors for X and Y axis; "ElementHistogram2D(help)" shows internal options

* PlanarityAtoms : Returns the chi-squared value of the set of atoms from a perfect plane; "PlanarityAtoms(help)" shows internal options

* RDF : computes the radial distribution function using a given atom property; "RDF(help)" shows internal options

* RDFGrid : computes the radial distribution function using a given atom property, see

http://www.opus.ub.uni-erlangen.de/opus/volltexte/2007/736/pdf/MarkusHemmerDissertation.pdf, p. 65 for details; "RDFGrid(help)" shows internal options

* RDFSign : computes the radial distribution function using a given atom property; "RDFSign(help)" shows internal options

*********************************************************************************************************************************************************************************

**************************************************** Descriptors that can be natively computed for both Molecule and Atom *******************************************************

*********************************************************************************************************************************************************************************

By default, these are computed for each Atom, except when using GenerateDataset with a Molecule-level result descriptor. Molecule-level descriptors can be obtained instead by

using MoleculeSum(X), where X is any descriptor listed below

Customizable Operations

* 2DA : computes 2D (bond distance) autocorrelation of a specified atom property; "2DA(help)" shows internal options

* 2DASign : computes 2D (bond distance) autocorrelation of a specified atom property; "2DASign(help)" shows internal options

* 2DASmoothSign : computes 2D (bond distance) autocorrelation of a specified atom property; "2DASmoothSign(help)" shows internal options

* 3daClashSensitiveSign : computes the smooth radial distribution function using a given atom property; "3daClashSensitiveSign(help)" shows internal options

* 3daSmooth : computes the smooth radial distribution function using a given atom property; "3daSmooth(help)" shows internal options

* 3daSmoothSign : computes the smooth radial distribution function using a given atom property; "3daSmoothSign(help)" shows internal options

*********************************************************************************************************************************************************************************

******************************************************************** General-purpose descriptor operations **********************************************************************

*********************************************************************************************************************************************************************************

These can be used for any type of descriptor (Molecule-level, Atom-level, etc, or other general purpose descriptors)

Customizable Implementations

* ForEach : Allows creation of a set of descriptors by substituting a user-specified set of values in for a specific parameter; "ForEach(help)" shows internal options

* IterativePrediction : uses pre-trained machine learning model(s) on the given object; "IterativePrediction(help)" shows internal options

* IterativePredictionMean : computes the mean prediction of pre-trained machine learning model(s) on the given object, iteratively substituting the values returned by the

sub-model with the values returned by this model; "IterativePredictionMean(help)" shows internal options

* KohonenMapInfo : Computes distances to all members of one or more kohonen maps. May alternatively/additionally compute position of an element on the map. If both are

computed, output will be in the format: position in kohonen map 1, distances to nodes of kohonen map 1, position in kohonen map 2, etc.; "KohonenMapInfo(help)" shows internal

options

* Mapped :

Note that commas are automatically stripped from the given inputs; "Mapped(help)" shows internal options

* Prediction : uses pre-trained machine learning model(s) on the given object; "Prediction(help)" shows internal options

* PredictionInfo : Computes prediction-related information, including standard deviation, min, max, and ROC-curve derived statistics such as the PPV or local-PPV associated

with a prediction. Output will be statistics first, in the order provided; followed by metrics, in the order they are provided; "PredictionInfo(help)" shows internal options

* PredictionMean : computes the mean prediction of pre-trained machine learning model(s) on the given object; "PredictionMean(help)" shows internal options

* ToNumeric : Calculates a numeric descriptor and converts it into a string; "ToNumeric(help)" shows internal options

Operations

* 1st : For pairwise or higher dimension descriptor generation, selects the result from the 1st sub-object; "1st(help)" shows internal options

* 2nd : For pairwise or higher dimension descriptor generation, selects the result from the 2nd sub-object; "2nd(help)" shows internal options

* 3rd : For pairwise or higher dimension descriptor generation, selects the result from the 3rd sub-object; "3rd(help)" shows internal options

* 4th : For pairwise or higher dimension descriptor generation, selects the result from the 4th sub-object; "4th(help)" shows internal options

* Abs : Takes the absolute value of a descriptor; "Abs(help)" shows internal options

* Cos : Takes the cosine of a descriptor; "Cos(help)" shows internal options

* DefineNaN : replaces undefined/NaN values in a descriptor with another value or descriptor; "DefineNaN(help)" shows internal options

* DescriptorMax : Returns the max of a descriptor; "DescriptorMax(help)" shows internal options

* DescriptorMean : Returns the mean of a descriptor; "DescriptorMean(help)" shows internal options

* DescriptorMin : Returns the min of a descriptor; "DescriptorMin(help)" shows internal options

* DescriptorRange : Returns the range of a descriptor; "DescriptorRange(help)" shows internal options

* DescriptorStandardDeviation : Returns the standarddeviation of a descriptor; "DescriptorStandardDeviation(help)" shows internal options

* DescriptorSum : Returns the sum of a descriptor; "DescriptorSum(help)" shows internal options

* Divide : Divides two descriptors (binary true/false); "Divide(help)" shows internal options

* Equal : Equals two descriptors (binary true/false); "Equal(help)" shows internal options

* Exp : Takes the exponential of a descriptor; "Exp(help)" shows internal options

* Exponentiate : Exponentiates two descriptors (binary true/false); "Exponentiate(help)" shows internal options

* Greater : Greaters two descriptors (binary true/false); "Greater(help)" shows internal options

* GreaterEqual : GreaterEquals two descriptors (binary true/false); "GreaterEqual(help)" shows internal options

* Less : Lesss two descriptors (binary true/false); "Less(help)" shows internal options

* LessEqual : LessEquals two descriptors (binary true/false); "LessEqual(help)" shows internal options

* Ln : Takes the natural log of a descriptor; "Ln(help)" shows internal options

* Log : Takes the base-10 logarithm of a descriptor; "Log(help)" shows internal options

* MaxIndex : computes the index of maximum value for the given descriptor; "MaxIndex(help)" shows internal options

* MinIndex : computes the index of minimum value for the given descriptor; "MinIndex(help)" shows internal options

* Mod : Mods two descriptors (binary true/false); "Mod(help)" shows internal options

* Negative : gives the negative of the given argument of a descriptor; "Negative(help)" shows internal options

* Not : 1 if the argument is exactly 0, otherwise returns 0 of a descriptor; "Not(help)" shows internal options

* NotEqual : NotEquals two descriptors (binary true/false); "NotEqual(help)" shows internal options

* OuterProduct : Outer product of two descriptors; "OuterProduct(help)" shows internal options

* RankAsc : rank property values in ascending order. e.g. 0.2 0.21 0.5 0.1 -> 1 2 3 0Duplicate values receive the same rank (averaged) assigned, so 1 1 4 -> 0.5 0.5 2;

"RankAsc(help)" shows internal options

* RankDesc : rank property values in descending order. e.g. 0.2 0.21 0.5 0.1 -> 2 1 0 3Duplicate values receive the same rank (averaged) assigned, so 1 1 4 -> 1.5 1.5 0;

"RankDesc(help)" shows internal options

* Rescale : Rescales values relative to the Molecule values, specifically, computes: (X-MoleculeMean(X))/MoleculeStd(X). In the event that std is 0, returns 0;

"Rescale(help)" shows internal options

* SetNaNToDefinedDescriptorMean : replaces undefined/NaN values in a descriptor with the DescriptorMean of the defined values;

"SetNaNToDefinedDescriptorMean(help)" shows internal options

* Sin : Takes the sine of a descriptor; "Sin(help)" shows internal options

* SortAsc : sort property values in ascending order; "SortAsc(help)" shows internal options

* SortDesc : sort property values in descending order; "SortDesc(help)" shows internal options

* Sqr : squares the argument of a descriptor; "Sqr(help)" shows internal options

* Sqrt : Takes the square root of a descriptor; "Sqrt(help)" shows internal options

* Subtract : Subtracts two descriptors (binary true/false); "Subtract(help)" shows internal options

Customizable Operations

* Add : Adds two descriptors (binary true/false); "Add(help)" shows internal options

* Combine : Array of descriptors; "Combine(help)" shows internal options

* Limit : limit descriptor values to a specified range; "Limit(help)" shows internal options

* Multiply : Multiplys two descriptors (binary true/false); "Multiply(help)" shows internal options

* Partial : selects particular values (by index) of another descriptor; "Partial(help)" shows internal options

* Sigmoid : Amplitude / ( 1 + exp(-(x-x_offset)/slope)) + y_offset; "Sigmoid(help)" shows internal options

* WithinRange : This descriptor takes on a value of 1 if a descriptor's mean value is within a range, or 0 otherwise; "WithinRange(help)" shows internal options

* WithinRangeGaussian : Returns a value between 0 and 1 based on where another descriptor's value falls relative to a given mean value;

"WithinRangeGaussian(help)" shows internal options

* WithinRangeSmooth : This descriptor gives values between 0.0 and 1.0 depending on if another descriptor's magnitude is within a specified range. This descriptor's value

decreases smoothly as calculated by a sinusoidal transition (sigmoid-like); "WithinRangeSmooth(help)" shows internal options

Other strings will be interpreted as follows:

* : given a number, returns that number, otherwise retrieves the value of the misc property by that name; "(help)" shows internal options

<min_value> min/left boundary of the histogram, range: [-1.79769e+308,1.79769e+308]

<bin_size> size of each bin in the histogram, range: [0,1.79769e+308]

<number_bins> number of bins in the histogram, range: [0,18446744073709551615]

-string_histogram : properties containing space-delimited strings, This flag can be followed by any number of

<property> property, choose any implementation of bcl::chemistry::StringPropertyInterface:

Basic Implementations

* AtomTypes : Retrieves the atom types of all atoms in a molecule

* BondTypes : Retrieves the bond types of all bonds in a molecule

* Chirality : Retrieves the chirality of all atoms in a molecule

* Name : Retrieves the name of the molecule

* SumFormula : Retrieves the sum formula a molecule

Customizable Implementations

* Cached : Retrieve a property already cached on the molecule; "Cached(help)" shows internal options

* Numeric : Calculates a numeric molecular or atomic property from the molecule and converts it into a string; "Numeric(help)" shows internal options

Customizable Operations

* MappedString : uses one property as a key to return the corresponding value from a 2-column table in a file

Input files have rows with a key, followed by spaces or tabs, followed by a value string

2 5.0 6.0 12.0

1 3.0 14.0 18.0; "MappedString(help)" shows internal options

Other strings will be interpreted as follows:

* : Calculates a numeric molecular or atomic property from the molecule and converts it into a string; "(help)" shows internal options

-output_histogram : filename to write out histograms

<output_histogram> filename to write out histograms, default: "molecule.histogram.txt"

-statistics : properties on which to take statistics (output file is controlled by -output_histogram), This flag can be followed by any number of

<statistics> properties on which to take statistics (output file is controlled by -output_histogram),

choose any implementation of bcl::chemistry::StringPropertyInterface (already listed)

-tabulate : properties to put into a csv file; file will always contain molecule index in the first column, This flag can be followed by any number of

<property> property, choose any implementation of bcl::chemistry::StringPropertyInterface (already listed)

-output_table : filename to write out csv of properties given in -table

<output_table> filename to write out csv of properties given in -table, default: "properties.csv"

-remove : miscellaneous properties to remove from all molecules, This flag can be followed by any number of

<property> miscellaneous property to remove from all molecules

-remove_all : removes all properties from all molecules

-add : descriptors / properties to add to all molecules, This flag can be followed by any number of

<property> descriptors / properties to add to all molecules, Any of the following:

choose any implementation of bcl::chemistry::StringPropertyInterface (already listed)

Or allowed values: {Index}

Or choose any implementation of Molecule/Atom Numeric Descriptor (already listed)

-add_strings : strings to add as descriptors / properties to all molecules, This flag can be followed by any number of

<property> name of descriptor / property to add

<string> the string assigned to the property / descriptor name for all molecules

-rename : for each pair of properties listed, change the name from the first property to the second, This flag can be followed by any number of

<initial_property_name> property name as it is currently in the molecule

<desired_property_name> property name to rename the initial property name to

-output : sdf filename for where to write out molecules

<filename> sdf filename for where to write out molecules, optional

=============================================================================================================================================================================================

additional arguments (including the application) can be loaded from files by passing e.g. @my_file.txt

If no application is specified, attempts to read commands from bcl_commands.txt

***BCL v4.2.0 descriptors extended***

choose any Molecule / Atom Numeric descriptor :

*********************************************************************************************************************

******************************************** Descriptors of Molecules ***********************************************

*********************************************************************************************************************

Basic Implementations

* Atom_Identity : 1, for any atom or molecule. The Atom_ prefix is purely for backwards-compatibility. In new

descriptor files, the use of Constant(1) is preferred. Alias for : Constant(1)

* BondGirth : User-defined. Alias for :

DescriptorSum(2DAMax(steps=96,property=Atom_Identity,substitution_value=nan))

* CovalentSurfaceArea : covalent surface area. Alias for : MoleculeSum(Atom_CovalentSurfaceArea)

* CovalentVolume : covalent volume. Alias for : MoleculeSum(Atom_CovalentVolume)

* EstCovSurfaceArea : Estimated covalent surface area (conformation independent). Alias for :

MoleculeSum(Atom_EstCovalentSurfaceArea)

* EstVdwSurfaceArea : Estimated van-der-waals surface area (conformation independent), using element-based VDW

radii. Alias for : MoleculeSum(Atom_EstVdwSurfaceArea)

* EstVdwSurfaceAreaCSD : Estimated van-der-waals surface area (conformation independent), using more accurate,

CSD-derived atom-type VdW radii, which tend to give larger SAs to H than EstVdwSurfaceArea. Alias for :

MoleculeSum(Atom_EstVdwSurfaceAreaCSD)

* Girth : calculates the girth of a molecule

* HbondAcceptor : # hydrogen bond acceptors. Alias for : MoleculeSum(Atom_HbondAcceptors)

* HbondDonor : # hydrogen bond donors. Alias for : MoleculeSum(Atom_HbondDonors)

* LipinskiDruglike : Returns 1 if the number of Lipinski violations is less than 2. Alias for :

Less(lhs=LipinskiViolations,rhs=Constant(2))

* LipinskiViolations : calculates how many times a molecule violates Lipinski's Rule of 5

* LipinskiViolationsVeber : calculates how many times a molecule violates Veber's variant of the Lipinski's Rule of

5 (<10 rotatable bonds, Polar SA < 140 A^2; see J. Med. Chem., 2002, 45 (12), pp 2615–2623)

* LogP : Calculates logp using the heuristic formula from http://pubs.acs.org/doi/full/10.1021/ci010315d

* LogP2008 : Calculates logp using the heuristic formula from http://pubs.acs.org/doi/full/10.1021/ci010315d

* MACCS : Retrieves the MACCS of each molecule using a modified pubchem MACCS fingerprint. See

ftp://ftp.ncbi.nlm.nih.gov/pubchem/specifications/pubchem_fingerprints.txt for details on the pubchem values

* MaxRingSize : Largest unbridged ring inside the molecule; returns 2 for ringless molecules. Alias for :

MoleculeMax(AtomMaxRingSize)

* MinRingSize : Smallest ring inside the molecule; returns 10000 for ringless molecules. Alias for :

MoleculeMin(AtomMinRingSize)

* MoleculeComplexity : calculates the complexity of a molecule (see http://www.jcheminf.com/content/1/1/8)

* MoleculeOneFourClashScore : Calculates the sum of one-four clash scores in the molecule

* MoleculeTotalBondEnergy : Calculates the sum of bond energies derived from the statistical bond potential

* MoleculeVDWScore : Calculates the total molecule VDW score normalized by the number of atoms in the molecule

* NAromaticMacrocyclicRings : Number of Aromatic macrocyclic (>8 atoms) rings in the molecule

* NAromaticRings : Number of Aromatic rings in the molecule

* NAtoms : Number of atoms. Alias for : NElements

* NConjugatedMacrocyclicRings : Number of Conjugated macrocyclic (>8 atoms) rings in the molecule

* NConjugatedRings : Number of Conjugated rings in the molecule

* NElements : The number of Atom in the Molecule

* NHeavyAtoms : Number of non-hydrogen atoms in the molecule. Alias for : MoleculeSum(IsNotH)

* NMacrocyclicRings : Number of macrocyclic (>8 atoms) rings in the molecule

* NNonconjugatedMacrocyclicRings : Number of Nonconjugated macrocyclic (>8 atoms) rings in the molecule

* NNonconjugatedRings : Number of Nonconjugated rings in the molecule

* NRings : Number of rings in the molecule

* NRotBond : calculates the number of non-trivially rotatable bonds

* NRotBondSym : calculates the number of non-trivially rotatable bonds

* NStereo : Number of stereocenters. Alias for : MoleculeSum(NotEqual(Atom_Stereocenters,Constant(0)))

* Polarizability : total polarizability. Alias for : MoleculeSum(Atom_Polarizability)

* TopologicalPolarSurfaceArea : topological polar surface area. Alias for :

MoleculeSum(Atom_TopologicalPolarSurfaceArea)

* TotalCharge : Sum of sigma and pi charges. Alias for : MoleculeSum(Atom_TotalCharge)

* TotalFormalCharge : Sum of formal charges on the molecule. Alias for : MoleculeSum(Atom_FormalCharge)

* VdwSurfaceArea : van der waals surface area. Alias for : MoleculeSum(Atom_VDWSurfaceArea)

* VdwVolume : van der waals volume. Alias for : MoleculeSum(Atom_VDWVolume)

* Weight : Molecular weight (amu). Alias for : MoleculeSum(Atom_Mass)

* XLogP : Calculates LogP using a multi-tasking deep neural network trained to predict LogP, LogS, and hydration

free energy

Customizable Implementations

* 3DInterHBondCode : Represents hydrogen bond interactions in relative distance and angle correlation bins

Default label : 3DInterHBondCode(step size=0.5,angle size=7214877045470555745,steps=14,misc property

id="",reference_conformer="",mol_a_atom_indices_filename="",mol_b_atom_indices_filename="")

Parameters:

<step size> size of each step in angstroms, default: "0.50", Any decimal (floating-point) value >= 0.01 <= 100

<angle size> size of each angle increment in degrees, default: "8", Any non-negative integer >= 1 <= 180

<steps> # of steps/bins (each of size = step size) used in the function, default: "8",

Any non-negative integer >= 1 <= 1000000

<misc property id> misc property name of corresponding protein binding pocket for current molecule, default: "",

any string

<reference_conformer> the molecule (e.g. protein binding pocket) whose autocorrelation is being compared against

the input molecule descriptor set, default: "", any string

<mol_a_atom_indices_filename> get the atom indices from molecule A contributing to the 3da interaction and output

to this file; recommended to only use this for discrete properties, e.g.

Partial(3DAPairRSAsym050(Multiply(Atom_HydrophobicTernary,Atom_Polarizability),Atom_IsInAromaticRingTernary),indices(23)),default: "",

any string

<mol_b_atom_indices_filename> get the atom indices from molecule B contributing to the 3da interaction and output

to this file; recommended to only use this for discrete properties, e.g.

Partial(3DAPairRSAsym050(Multiply(Atom_HydrophobicTernary,Atom_Polarizability),Atom_IsInAromaticRingTernary),indices(23)),default: "",

any string

* AffinityNet : Calculates binding affinity (in units of pKd)

Default label :

AffinityNet(misc_property_id=receptor_filename,receptor_filename="",report_dg=0,temperature=298.75)

Parameters:

<misc_property_id> misc property name of corresponding protein binding pocket for current molecule,

default: "receptor_filename", any string

<receptor_filename> the filename of the protein binding pocket of interest, default: "", any string

<report_dg> reports the result as binding free energy in kcal/mol at the specified temperature, default: "0",

Any non-negative integer

<temperature> temperature for binding free energy conversion; no effect if report_dg = 0, default: "298.75",

Any decimal (floating-point) value

* AffinityNetAD : Calculates binding affinity (in units of pKd) weighted by 1.0 minus the model applicability

domain score

Default label :

AffinityNetAD(misc_property_id=receptor_filename,receptor_filename="",report_dg=0,temperature=298.75)

Parameters:

<misc_property_id> misc property name of corresponding protein binding pocket for current molecule,

default: "receptor_filename", any string

<receptor_filename> the filename of the protein binding pocket of interest, default: "", any string

<report_dg> reports the result as binding free energy in kcal/mol at the specified temperature, default: "0",

Any non-negative integer

<temperature> temperature for binding free energy conversion; no effect if report_dg = 0, default: "298.75",

Any decimal (floating-point) value

* BuserMetric : calculates the buser similarity score between

Default label : BuserMetric(atom hashing type=Element,filename="")

Parameters:

<atom hashing type> Choose one of four: Element, ElemRC, Atom, AtomRC. element by default, default: "Element",

Allowed values: {Element, Atom}

<filename> File containing active compounds to compare with each query molecule, any existent file

* Constant : Returns a constant set of values

(anonymous) parameter: Container with at least 1 Any decimal (floating-point) value

* Default : given a number, returns that number, otherwise retrieves the value of the misc property by that name

(anonymous) parameter: number or misc property name, default: "", any string

* Define : Define an alias for a given descriptor

(anonymous) parameter: The LHS of the = sign will become an alias that can be used later (in the same file or

command) to refer to the RHS. Existing aliases cannot be overridden. Accepts , any object serialization tree

* DockANNScore : Calculates a docking score based on the local ppv classifying a pose as within 1.0 Angstroms of

the native pose weighted by the predicted affinity of the pose

Default label :

DockANNScore(misc_property_id=receptor_filename,receptor_filename="",report_dg=0,temperature=298.75)

Parameters:

<misc_property_id> misc property name of corresponding protein binding pocket for current molecule,

default: "receptor_filename", any string

<receptor_filename> the filename of the protein binding pocket of interest, default: "", any string

<report_dg> reports the result as binding free energy in kcal/mol at the specified temperature, default: "0",

Any non-negative integer

<temperature> temperature for binding free energy conversion; no effect if report_dg = 0, default: "298.75",

Any decimal (floating-point) value

* EntropyQHA : Sampling options for relative conformational entropy estimates. Descriptor output indices correspond

to the global_s, local_s, global_s - local_s, and -ln(local_s/global_s). The first 4 indices are actual entropy

estimates in J*K^-1*mol^-1, while the latter 4 indices are the PCA eigenvalue sums.

Default label : EntropyQHA(sampler=SampleConformations(rotamer_library=bcl::chemistry::RotamerLibraryInterface,conformation_comparer=SymmetryRMSD(consider

H=0,atom comparison=ElementType,bond comparison=FuzzyBondOrderAmideOrAromaticWithRingness,isomorphism

limit=1000),generate_3D=1,tolerance=0.25,max_cr_iterations=2,max_avg_clash=0.1,max_iterations=200,change_chirality=0,max_conformations=200,cluster=0,relative_random_dihedral_change_weight=0.01),local_ensemble="",global_ensemble="")

Parameters:

<sampler> sample configurational space across dihedral bins,

default: "(conformation_comparer=bcl::chemistry::ConformationComparisonInterface,tolerance=0.0,generate_3D=0,relative_random_dihedral_change_weight=0.0,cluster=false,max_iterations=10000,max_conformations=1000)", sample conformations of molecules.

To keep part of the molecule rigid, set an MDL property SampleByParts with the MDL (0-indexed) atom indices that

can be moved, e.g.

> <SampleByParts>

0 4 6 8 10

This would indicate that only dihedrals containing exclusively of atoms 1, 5, 7, 9, and 11 in the SD file can be

rotated.

Default label :

SampleConformations(rotamer_library=bcl::chemistry::RotamerLibraryInterface,conformation_comparer=SymmetryRMSD(consider

H=0,atom comparison=ElementType,bond comparison=FuzzyBondOrderAmideOrAromaticWithRingness,isomorphism

limit=1000),generate_3D=1,tolerance=0.25,max_cr_iterations=2,max_avg_clash=0.1,max_iterations=200,change_chirality=0,max_conformations=200,cluster=0,relative_random_dihedral_change_weight=0.01)

Parameters:

<rotamer_library> flag for rotamer library to use. Crystallographic open database is used by default,

default: "File", choose any implementation of bcl::chemistry::RotamerLibraryInterface:

Basic Implementations

* cod : Load fragments from the cod library

Customizable Implementations

* File : ; "File(help)" shows internal options

<conformation_comparer> method to compare conformers with, default: "SymmetryRMSD",

Calculates the root-mean-squared-deviation between two molecules that are identical onthe

constitutional level, and whose atoms have already been aligned

Default label : SymmetryRMSD(consider H=0,atom comparison=ElementType,bond

comparison=FuzzyBondOrderAmideOrAromaticWithRingness,isomorphism limit=1000)

Parameters:

<consider H> set to true if you want to consider RMSD of hydrogens atoms as well. This is usually a

bad idea in larger molecules, because the number of possible automorphisms of the molecule with

hydrogens grows exponentially. Only recommended for molecules with fewer than 15 heavy atoms, default:

"false", Any non-negative integer

<atom comparison> Used to decide which atom types can be considered equivalent,

default: "ElementType",

Allowed values: {Identity, ElementType, AtomType, AtomTypeAndChirality,

AtomTypeAndComplexRingChirality, AtomTypeAndSymmetry, AtomTypeAndHasSymmetry,

AtomTypeAndNumberHydrogens, AtomTypeAndNumberHydrogensOnRingAtoms,

AtomTypeAndNumberHydrogensOnRingsAndDistinguishHydrogens, AtomTypeAndDistinguishHydrogens,

CIPPriorityHighToLow, CouldHaveSubstituents}

<bond comparison> Used to decide whether bonds are similar enough to consider equivalent,

default: "FuzzyBondOrderAmideOrAromaticWithRingness",

Allowed values: {Identity, BondOrder, NumberOfElectrons, Conjugation, IsConjugated, IsAromatic,

IsAmide, IsInRing, BondOrderInRingOrAromatic, BondOrderOrAromatic, BondOrderAmideOrAromatic,

BondOrderOrAromaticWithRingness, BondOrderAmideOrAromaticWithRingness,

FuzzyBondOrderWithIsometryOrAromaticWithRingness, FuzzyBondOrderAmideOrAromaticWithRingness,

ConstitutionalBondType, BondOrderWithIsometry, Isometry, IsIsometric,

BondOrderWithIsometryOrAromatic, BondOrderAmideWithIsometryOrAromaticWithRingness,

ConfigurationalBondType}

<isomorphism limit> If the calculation is prohibitively slow for highly-symmetric molecules, set the

isomorphism limit to speed up the computation. There are exponentially diminishing returns for

having all isomorphisms present, so usually 10-100 is sufficient for most purposes, default: "1000", Any

non-negative integer

<generate_3D> true to completely discard the input conformation and rebuild a 3D conformer from scratch. ,

default: "false", Any non-negative integer

<tolerance> amount of tolerance allowed between two conformers, default: "0.25",

Any decimal (floating-point) value

<max_cr_iterations> max number of clash resolution iterations performed (as fraction of # dihedrals & bond

angles).More iterations will produced less clashed molecules but may take much longer. Often it is faster to

just make more molecules and discard the excessively clashed ones if they can't be resolved in a small

fraction of moves (e.g. 10% of the total dihedrals + bond angles, default: "0.1", Any decimal

(floating-point) value

<max_avg_clash> max average clash allowed for conformations =sum(vdw_overlap for all atoms in molecule) / atoms

in molecule. larger values will yield potentially more clashed conformers for some molecules, default: "0.1",

Any decimal (floating-point) value

<max_iterations> number of iterations, default: "1000", Any non-negative integer

<change_chirality> change chirality during conformation sampling, default: "false", Any non-negative integer

<max_conformations> maximum number conformations to generate per molecule, default: "200",

Any non-negative integer

<cluster> Set to true to select up to max_conformations so as to maximize coverage of conformational space by

performing greedy clustering on all returned conformations. This option should only be turned off when

generating boltzmann ensembles of conformers typically. Otherwise, better speed and rmsd recovery will result

just by decreasing # of iterations, default: "True", Any non-negative integer

<relative_random_dihedral_change_weight> Weight for random dihededral changes relative to fragment based

changes. This is desireable for exhaustive sampling or when parts of a fragment are very poorly represented

in the fragment library, default: "0.0", Any decimal (floating-point) value

<local_ensemble> input a pre-generated local ensemble, default: "", any string

<global_ensemble> input a pre-generated global ensemble, default: "", any string

* IsConstitutionDruglike : Returns 1 if the molecule is druglike and 0 otherwise

Default label :

IsConstitutionDruglike(min_weight=10,max_weight=600,max_ring_size=10,min_log_p=-1,max_log_p=5,max_tpsa=140,max_hbda=12,max_n_rot_bonds=10,max_bond_score=0.1,max_complexity=4,max_n_f=5,max_n_cl=2,max_n_br=1,max_n_i=1,max_total_halogens=5,max_n_ring_halogens=3,max_n_non_aromatic_cl_br_i=0,enforce_hitlike=0,enforce_druglike_rings=0,enforce_druglike_fragments=0)

Parameters:

<min_weight> minimum molecular weight considered to be druglike, default: "10.0",

Any decimal (floating-point) value

<max_weight> maximum molecular weight considered to be druglike, default: "600.0",

Any decimal (floating-point) value

<max_ring_size> maximum number of heavy atoms in a ring considered to be druglike, default: "10",

Any non-negative integer

<min_log_p> minimum logP considered to be druglike, default: "-1.0", Any decimal (floating-point) value

<max_log_p> maximum logP considered to be druglike, default: "5.0", Any decimal (floating-point) value

<max_tpsa> maximum topological polar surface area considered to be druglike, default: "140",

Any decimal (floating-point) value

<max_hbda> maximum number of hydrogen bond donors + acceptors considered to be druglike, default: "12",

Any non-negative integer

<max_n_rot_bonds> maximum number of rotatable bonds considered to be druglike, default: "10",

Any non-negative integer

<max_bond_score> maximum bond propensity score considered to be druglike, default: "0.10",

Any decimal (floating-point) value

<max_complexity> maximum molecule complexity considered to be druglike, default: "4.0",

Any decimal (floating-point) value

<max_n_f> maximum number of fluorine atoms considered to be druglike, default: "5", Any non-negative integer

<max_n_cl> maximum number of chlorine atoms considered to be druglike, default: "2", Any non-negative integer

<max_n_br> maximum number of bromine atoms considered to be druglike, default: "1", Any non-negative integer

<max_n_i> maximum number of iodine atoms considered to be druglike, default: "1", Any non-negative integer

<max_total_halogens> maximum total number of halogen atoms considered to be druglike, default: "5",

Any non-negative integer

<max_n_ring_halogens> maximum total number of halogen atoms that can be on an aromatic ring system,

default: "3", Any non-negative integer

<max_n_non_aromatic_cl_br_i> maximum total number of Cl, Br, and I atoms that can be on a non-aromatic system,

default: "0", Any non-negative integer

<enforce_hitlike> require that the molecule also fullfil hit-like criteria;criteria based on a simple decision

tree trained with the data providedin Bickerton et al., 2012, Quantifying the chemical beauty of drugs,

10.1038/nchem.1243.Specifically, it addresses the following question posed to medicinal chemists:'If this

compound came out of a primary screen, would you perform hit optimization on it?', optional, Any non-negative integer

<enforce_druglike_rings> require that the molecule only contain rings found in the druglike ring database,

optional,Any non-negative integer

<enforce_druglike_fragments> require that the molecule only be composed of ECFP fragments found in the ECFP

fragment database, optional, Any non-negative integer

* IsConstitutionDruglikeAndHitlike : Returns 1 if the molecule is druglike and 0 otherwise

Default label :

IsConstitutionDruglikeAndHitlike(min_weight=10,max_weight=600,max_ring_size=10,min_log_p=-1,max_log_p=5,max_tpsa=140,max_hbda=12,max_n_rot_bonds=10,max_bond_score=0.1,max_complexity=4,max_n_f=5,max_n_cl=2,max_n_br=1,max_n_i=1,max_total_halogens=5,max_n_ring_halogens=3,max_n_non_aromatic_cl_br_i=0,enforce_hitlike=1,enforce_druglike_rings=0,enforce_druglike_fragments=0)

Parameters:

<min_weight> minimum molecular weight considered to be druglike, default: "10.0",

Any decimal (floating-point) value

<max_weight> maximum molecular weight considered to be druglike, default: "600.0",

Any decimal (floating-point) value

<max_ring_size> maximum number of heavy atoms in a ring considered to be druglike, default: "10",

Any non-negative integer

<min_log_p> minimum logP considered to be druglike, default: "-1.0", Any decimal (floating-point) value

<max_log_p> maximum logP considered to be druglike, default: "5.0", Any decimal (floating-point) value

<max_tpsa> maximum topological polar surface area considered to be druglike, default: "140",

Any decimal (floating-point) value

<max_hbda> maximum number of hydrogen bond donors + acceptors considered to be druglike, default: "12",

Any non-negative integer

<max_n_rot_bonds> maximum number of rotatable bonds considered to be druglike, default: "10",

Any non-negative integer

<max_bond_score> maximum bond propensity score considered to be druglike, default: "0.10",

Any decimal (floating-point) value

<max_complexity> maximum molecule complexity considered to be druglike, default: "4.0",

Any decimal (floating-point) value

<max_n_f> maximum number of fluorine atoms considered to be druglike, default: "5", Any non-negative integer

<max_n_cl> maximum number of chlorine atoms considered to be druglike, default: "2", Any non-negative integer

<max_n_br> maximum number of bromine atoms considered to be druglike, default: "1", Any non-negative integer

<max_n_i> maximum number of iodine atoms considered to be druglike, default: "1", Any non-negative integer

<max_total_halogens> maximum total number of halogen atoms considered to be druglike, default: "5",

Any non-negative integer

<max_n_ring_halogens> maximum total number of halogen atoms that can be on an aromatic ring system,

default: "3", Any non-negative integer

<max_n_non_aromatic_cl_br_i> maximum total number of Cl, Br, and I atoms that can be on a non-aromatic system,

default: "0", Any non-negative integer

<enforce_hitlike> require that the molecule also fullfil hit-like criteria;criteria based on a simple decision

tree trained with the data providedin Bickerton et al., 2012, Quantifying the chemical beauty of drugs,

10.1038/nchem.1243.Specifically, it addresses the following question posed to medicinal chemists:'If this

compound came out of a primary screen, would you perform hit optimization on it?', optional, Any non-negative integer

<enforce_druglike_rings> require that the molecule only contain rings found in the druglike ring database,

optional,Any non-negative integer

<enforce_druglike_fragments> require that the molecule only be composed of ECFP fragments found in the ECFP

fragment database, optional, Any non-negative integer

* MiscProperty : retrieves a molecule property that cannot be calculated directly by the bcl, e.g. biological data

Default label : MiscProperty("",values per molecule=0)

Parameters:

name of the miscellaneous property, any string

<values per molecule> expected number of values per molecule, default: "1",

Any non-negative integer >= 1 <= 100000

* MolAlignPharmScore : Performs quick property-based molecular alignment with BCL::MolAlign and returnsthe RMSDX

score as well as atomic overlap metrics.

Default label : MolAlignPharmScore(scaffold="",alignment type=flexible,max atom

distance=1,properties="",alignment

options=PsiFlexField(properties="",property_weights(1),max_atom_distance_criterion=1,optimizing_weights=0,anchor_weight=0.01,linear_mismatch_penalty=0.01,heavy_mismatch_penalty_fraction=0.6,heavy_mismatch_penalty=2,weight_file_path="",data_file_name="",iterations=400,max_unimproved_steps=160,number_rigid_trajectories=5,number_outputs=1,align_to_scaffold=0,initial_rand_rotation=0,exclusion_indices_a="",exclusion_indices_b="",pose_tolerance=0.125,pose_score_threshold=2,flip_prob=0.06,big_rot_prob=0.06,bond_swap_prob=0.06,bond_align_prob=0.3,bond_align3_prob=0.2,bond_align_inf_prob=0.1,small_rot_prob=0.12,small_trans_prob=0.12,conf_swap_prob=0.06,potential_map_filename="",rigid_mol_b=1,conformer_pairs=100,top_pairs=3,filter_iterations=600,filter_limit=240,refinement_iterations=200,refinement_limit=80,number_flexible_trajectories=5,fraction_filtered_initially=0.25,fraction_filtered_iteratively=0.5,sample_conformers=SampleConformations(rotamer_library=bcl::chemistry::RotamerLibraryInterface,conformation_comparer=SymmetryRMSD(consider

H=0,atom comparison=ElementType,bond comparison=FuzzyBondOrderAmideOrAromaticWithRingness,isomorphism

limit=1000),generate_3D=1,tolerance=0.25,max_cr_iterations=2,max_avg_clash=0.1,max_iterations=200,change_chirality=0,max_conformations=200,cluster=0,relative_random_dihedral_change_weight=0.01),conf_properties=""),property

weights="")

Parameters:

<scaffold> the molecule to which the inputs are aligned, any existent file

<alignment type> the type of alignment to perform; options are 'flexible' or 'rigid', default: "flexible",

any string

<max atom distance> distance for computing MolAlign mutually matched atoms and VoxelGrid neighbors,

default: "1.0", Any decimal (floating-point) value

<properties> atom properties to consider, use multiply(Constant(X),property y) for weighting,

default: "Combine(Atom_Identity)", Array of descriptors

(anonymous) parameter: descriptors to concatenate,

Container with at least 1 choose any implementation of Molecule/Atom Numeric Descriptor (already

listed)

<alignment options> options for the flexible alignment procedure,

default: "(rigid mol b = true,number rigid trajectories=3,number flexible trajectories=3,fraction filtered initially=0.75,fraction filtered iteratively=0.50,iterations=100,filter iterations=50,refinement iterations=50,align to scaffold = false,conformer pairs = 10,number outputs = 1,sample conformers=SampleConformations(conformation_comparer=RMSD,generate_3D=0,tolerance=0.50,rotamer_library = cod,max_iterations=100,max_conformations=10,cluster=true,clash_tolerance=0.4,change_chirality=0))", Aligns and computes property RMSD for molecule conformer libraries

Default label :

PsiFlexField(properties="",property_weights(1),max_atom_distance_criterion=1,optimizing_weights=0,anchor_weight=0.01,linear_mismatch_penalty=0.01,heavy_mismatch_penalty_fraction=0.6,heavy_mismatch_penalty=2,weight_file_path="",data_file_name="",iterations=400,max_unimproved_steps=160,number_rigid_trajectories=5,number_outputs=1,align_to_scaffold=0,initial_rand_rotation=0,exclusion_indices_a="",exclusion_indices_b="",pose_tolerance=0.125,pose_score_threshold=2,flip_prob=0.06,big_rot_prob=0.06,bond_swap_prob=0.06,bond_align_prob=0.3,bond_align3_prob=0.2,bond_align_inf_prob=0.1,small_rot_prob=0.12,small_trans_prob=0.12,conf_swap_prob=0.06,potential_map_filename="",rigid_mol_b=1,conformer_pairs=100,top_pairs=3,filter_iterations=600,filter_limit=240,refinement_iterations=200,refinement_limit=80,number_flexible_trajectories=5,fraction_filtered_initially=0.25,fraction_filtered_iteratively=0.5,sample_conformers=SampleConformations(rotamer_library=bcl::chemistry::RotamerLibraryInterface,conformation_comparer=SymmetryRMSD(consider

H=0,atom comparison=ElementType,bond comparison=FuzzyBondOrderAmideOrAromaticWithRingness,isomorphism

limit=1000),generate_3D=1,tolerance=0.25,max_cr_iterations=2,max_avg_clash=0.1,max_iterations=200,change_chirality=0,max_conformations=200,cluster=0,relative_random_dihedral_change_weight=0.01),conf_properties="")

Parameters:

<properties> atom properties to consider, use multiply(Constant(X),property y) for weighting,

default:

"Combine(Define(ChargeNegative=Multiply(Less(lhs=Atom_SigmaCharge,rhs=Constant(0)),Atom_SigmaCharge)),Define(ChargePositive=Multiply(Greater(lhs=Atom_SigmaCharge,rhs=Constant(0)),Atom_SigmaCharge)),Define(HbondAcceptorsStrict=Multiply(Atom_HbondAcceptors,Not(Atom_HbondDonors),LessEqual(lhs=BondTypeCount,rhs=Constant(2)))),Define(ENegOffset=Subtract(lhs=Atom_ElectroNegativity,rhs=Constant(2.5))),Define(IsENeg=Greater(lhs=ENegOffset,rhs=Constant(0))),Define(PolarTernary=Subtract(lhs=Multiply(Add(HbondAcceptorsStrict,Atom_HbondDonors),Constant(2)),rhs=Constant(1))),Define(Atom_Hydrophobic=Not(DescriptorSum(Abs(Partial(indices(1,2,5,8),2DASign(property=PolarTernary,steps=3)))))),Atom_SigmaCharge,HbondAcceptorsStrict,Atom_HbondDonors,Atom_Polarizability,Atom_AromaticityAxes,Multiply(IsENeg,ENegOffset),Atom_Hydrophobic,Atom_VDWVolume)",

Array of descriptors

(anonymous) parameter: descriptors to concatenate,

Container with at least 1 choose any implementation of Molecule/Atom Numeric Descriptor (already

listed)

<property_weights> Weighting to give the properties. Typically these are the inverse standard deviations of the

properties over a representative set of molecules, which can be computed w/ molecule:Properties -statistics,

default: "(5,3.5,7,1.71,2.41,2.41,2.41,3.0,2.0,0.714)", Container with at least 1 Any decimal (floating-point)

value

<max_atom_distance_criterion> independent scoring: max atom distance when directly calling this class for

scoring, default: "1.0", Any decimal (floating-point) value

<optimizing_weights> string - perform molecule:Compare w/ SymmetryRMSD to native binding poses in EnsembleB,

default: "false", Any non-negative integer

<anchor_weight> deprecated; enter in scientific notation, default: "1.0e-3", Any decimal (floating-point) value

<linear_mismatch_penalty> this penalty is applied as 'penalty = linear_mismatch_penalty * (1.0 -

max_fraction_matched)'; enter in scientific notation, default: "1.0e-2", Any decimal (floating-point) value

<heavy_mismatch_penalty_fraction> molecule alignments where at least one molecule has fewer than this fraction

of its atoms mutually matched to atoms in the other molecule suffer the 'heavy_mismatch_penalty', default:

"0.6", Any decimal (floating-point) value

<heavy_mismatch_penalty> this penalty is applied as 'heavy_mismatch_penalty * ( Sqr(( 0.6 - max_matched) /

0.6))' when the fraction of matched atoms in the maximally matched molecule is less than

heavy_mismatch_penalty_fraction; enter in scientific notation, default: "2", Any decimal (floating-point)

value

<weight_file_path> path for outputting .csv files for weight optimization, default: "/tmp/", any string

<data_file_name> file tag for unweighted property data in ,csv, default: "unweighted_files", any string

<iterations> number_of_mc_iterations, default: "400", Any non-negative integer

<max_unimproved_steps> limit number unimproved mc iterations, default: "160", Any non-negative integer

<number_rigid_trajectories> number of mc trajectories for rigid (single conformer) alignment; each will be

initialized with a uniform max atom distance between the lower and upper limits, default: "5", Any

non-negative integer

<number_outputs> number of best pairs to output as SDF files, default: "1", Any non-negative integer

<align_to_scaffold> include AlignToScaffold as a move in addition to mutates, default: "false",

Any non-negative integer

<initial_rand_rotation> perform an initial random rotation within the range 0 - 180 degrees; may be useful to

assist with benchmarking, default: "false", Any non-negative integer

<output_aligned_mol_a> if true output molecule(s) A pose with the specified name, optional, any string

<output_aligned_mol_b> if true output molecule(s) B pose with the specified name, optional, any string

<exclusion_indices_a> atom indices (0-indexed) to be ignored during alignment scoring in molecule A,

default: "", any string

<exclusion_indices_b> atom indices (0-indexed) to be ignored during alignment scoring in molecule B,

default: "", any string

<pose_tolerance> real space symmetry rmsd tolerance to discriminate unique poses for output, default: "0.125",

Any decimal (floating-point) value

<pose_score_threshold> threshold below which the alignment is accepted as a valid pose, default: "2.0",

Any decimal (floating-point) value

<flip_prob> relative probability of performing a Flip alignment move, default: "0.06",

Any decimal (floating-point) value

<big_rot_prob> relative probability of performing a BigRotation alignment move, default: "0.06",

Any decimal (floating-point) value

<bond_swap_prob> relative probability of performing a BondSwap alignment move, default: "0.06",

Any decimal (floating-point) value

<bond_align_prob> relative probability of performing a BondAlign alignment move, default: "0.30",

Any decimal (floating-point) value

<bond_align3_prob> relative probability of performing a BondAlign3 alignment move, default: "0.20",

Any decimal (floating-point) value

<bond_align_inf_prob> relative probability of performing a BondAlignInf alignment move, default: "0.10",

Any decimal (floating-point) value

<small_rot_prob> relative probability of performing a SmallRotation alignment move, default: "0.12",

Any decimal (floating-point) value

<small_trans_prob> relative probability of performing a SmallTranslation alignment move, default: "0.12",

Any decimal (floating-point) value

<conf_swap_prob> relative probability of performing a ConformerSwap alignment move, default: "0.06",

Any decimal (floating-point) value

<movie> string - save snapshots from MCM trajectory - WARNING - large file sizes easily accumulate, optional,

any string

<potential_map_filename> string - filename of pair potential map, default: "new_potentials.txt", any string

<rigid_mol_b> do not perform conformational sampling for molecule b, default: "true", Any non-negative integer

<conformer_pairs> number of pairs of conformers sampled, default: "100", Any non-negative integer

<top_pairs> number of best pairs within each trajectory included during inter-trajectory scoring,

default: "5", Any non-negative integer

<filter_iterations> number of mc iterations after initial mc, default: "600", Any non-negative integer

<filter_limit> max number of unimproved mc iterations after initial mc, default: "240",

Any non-negative integer

<refinement_iterations> number of mc iterations on the final molecule(s), default: "200",

Any non-negative integer

<refinement_limit> max number of unimproved mc iterations during final refinement, default: "80",

Any non-negative integer

<number_flexible_trajectories> number of independent flexible trajectories to run, default: "5",

Any non-negative integer

<fraction_filtered_initially> set the fraction of best conformer pairs to be saved after first pass,

default: "0.25", Any decimal (floating-point) value

<fraction_filtered_iteratively> fraction of best pairs saved with each subsequent iteration until best pair,

default: "0.50", Any decimal (floating-point) value

<sample_conformers> makes conformers of molecules,

default:

"(conformation_comparer=SymmetryRMSD,tolerance=0.25,generate_3D=0,cluster=true,max_iterations=2000,max_conformations=200,change_chirality=0)",

sample conformations of molecules.

To keep part of the molecule rigid, set an MDL property SampleByParts with the MDL (0-indexed) atom indices

that can be moved, e.g.

> <SampleByParts>

0 4 6 8 10

This would indicate that only dihedrals containing exclusively of atoms 1, 5, 7, 9, and 11 in the SD file can

be rotated.

Default label :

SampleConformations(rotamer_library=bcl::chemistry::RotamerLibraryInterface,conformation_comparer=SymmetryRMSD(consider

H=0,atom comparison=ElementType,bond comparison=FuzzyBondOrderAmideOrAromaticWithRingness,isomorphism

limit=1000),generate_3D=1,tolerance=0.25,max_cr_iterations=2,max_avg_clash=0.1,max_iterations=200,change_chirality=0,max_conformations=200,cluster=0,relative_random_dihedral_change_weight=0.01)

Parameters:

<rotamer_library> flag for rotamer library to use. Crystallographic open database is used by default,

default: "File", choose any implementation of bcl::chemistry::RotamerLibraryInterface (already

listed)

<conformation_comparer> method to compare conformers with, default: "SymmetryRMSD",

Calculates the root-mean-squared-deviation between two molecules that are identical onthe

constitutional level, and whose atoms have already been aligned

Default label : SymmetryRMSD(consider H=0,atom comparison=ElementType,bond

comparison=FuzzyBondOrderAmideOrAromaticWithRingness,isomorphism limit=1000)

Parameters:

<consider H> set to true if you want to consider RMSD of hydrogens atoms as well. This is usually

a bad idea in larger molecules, because the number of possible automorphisms of the molecule

with hydrogens grows exponentially. Only recommended for molecules with fewer than 15 heavy

atoms, default: "false", Any non-negative integer

<atom comparison> Used to decide which atom types can be considered equivalent,

default: "ElementType",

Allowed values: {Identity, ElementType, AtomType, AtomTypeAndChirality,

AtomTypeAndComplexRingChirality, AtomTypeAndSymmetry, AtomTypeAndHasSymmetry,

AtomTypeAndNumberHydrogens, AtomTypeAndNumberHydrogensOnRingAtoms,

AtomTypeAndNumberHydrogensOnRingsAndDistinguishHydrogens, AtomTypeAndDistinguishHydrogens,

CIPPriorityHighToLow, CouldHaveSubstituents}

<bond comparison> Used to decide whether bonds are similar enough to consider equivalent,

default: "FuzzyBondOrderAmideOrAromaticWithRingness",

Allowed values: {Identity, BondOrder, NumberOfElectrons, Conjugation, IsConjugated, IsAromatic,

IsAmide, IsInRing, BondOrderInRingOrAromatic, BondOrderOrAromatic, BondOrderAmideOrAromatic,

BondOrderOrAromaticWithRingness, BondOrderAmideOrAromaticWithRingness,

FuzzyBondOrderWithIsometryOrAromaticWithRingness, FuzzyBondOrderAmideOrAromaticWithRingness,

ConstitutionalBondType, BondOrderWithIsometry, Isometry, IsIsometric,

BondOrderWithIsometryOrAromatic, BondOrderAmideWithIsometryOrAromaticWithRingness,

ConfigurationalBondType}

<isomorphism limit> If the calculation is prohibitively slow for highly-symmetric molecules, set

the isomorphism limit to speed up the computation. There are exponentially diminishing returns

for having all isomorphisms present, so usually 10-100 is sufficient for most purposes, default: "1000",

Any non-negative integer

<generate_3D> true to completely discard the input conformation and rebuild a 3D conformer from scratch. ,

default: "false", Any non-negative integer

<tolerance> amount of tolerance allowed between two conformers, default: "0.25",

Any decimal (floating-point) value

<max_cr_iterations> max number of clash resolution iterations performed (as fraction of # dihedrals & bond

angles).More iterations will produced less clashed molecules but may take much longer. Often it is faster

to just make more molecules and discard the excessively clashed ones if they can't be resolved in a small

fraction of moves (e.g. 10% of the total dihedrals + bond angles, default: "0.1", Any decimal

(floating-point) value

<max_avg_clash> max average clash allowed for conformations =sum(vdw_overlap for all atoms in molecule) /

atoms in molecule. larger values will yield potentially more clashed conformers for some molecules,

default: "0.1", Any decimal (floating-point) value

<max_iterations> number of iterations, default: "1000", Any non-negative integer

<change_chirality> change chirality during conformation sampling, default: "false", Any non-negative integer

<max_conformations> maximum number conformations to generate per molecule, default: "200",

Any non-negative integer

<cluster> Set to true to select up to max_conformations so as to maximize coverage of conformational space by

performing greedy clustering on all returned conformations. This option should only be turned off when

generating boltzmann ensembles of conformers typically. Otherwise, better speed and rmsd recovery will

result just by decreasing # of iterations, default: "True", Any non-negative integer

<relative_random_dihedral_change_weight> Weight for random dihededral changes relative to fragment based

changes. This is desireable for exhaustive sampling or when parts of a fragment are very poorly represented

in the fragment library, default: "0.0", Any decimal (floating-point) value

<conf_properties> atom properties to consider, use multiply(Constant(X),property y) for weighting,

default: "(1)", Array of descriptors

(anonymous) parameter: descriptors to concatenate,

Container with at least 1 choose any implementation of Molecule/Atom Numeric Descriptor (already

listed)

<property weights> Weighting to give the properties. Typically these are the inverse standard deviations of the

properties over a representative set of molecules, which can be computed w/ molecule:Properties -statistics, default: "(1.0)", Container with at least 1 Any decimal (floating-point) value

* MoleculeAtomEnvironmentMap : Determines if molecule fragment radial atom environments exist in database

Default label :

MoleculeAtomEnvironmentMap(exclude_hydrogen_atoms=0,environment_size=2,atom_environment_file=chembl.h.atom_environment_hashmap.two_bonds.txt.gz)

Parameters:

<exclude_hydrogen_atoms> compare the local atom environments in the absence of hydrogen atoms; make sure to load

a hashmap generated in the absence of explicit hydrogen atoms; faster but not guaranteed to perform as well, default: "false", Any non-negative integer

<environment_size> atoms within this number of bonds of an atom constitutes the local environment, default: "2",

Any non-negative integer

<atom_environment_file> the file containing the pre-generated fragment atom environment counts,

default: "chembl.h.atom_environment_hashmap.two_bonds.txt.gz", any string

* MoleculeSimilarity : Searches for specified substructures within the query molecule. Returns 1 if a structure

exists in the query molecule

Default label : MoleculeSimilarity(filename="",method=bcl::chemistry::ConformationComparisonInterface)

Parameters:

<filename> File containing molecules to compare with each query molecule, any existent file

<method> the method of comparison to use, default: "LargestCommonSubstructureTanimoto",

choose any implementation of bcl::chemistry::ConformationComparisonInterface:

Basic Implementations

* Atom : Atomic Number and bond orders

* Element : Atomic Number and bond orders

* RMSD : Calculates the root-mean-squared-deviation between two molecules that are identical onthe

constitutional level, and whose atoms have already been aligned

* RealSpaceRMSD : Calculates the root-mean-squared-deviation between two molecules that are

identical onthe constitutional level, and whose atoms in the same order. No superimposition is

performed.

Customizable Implementations

* CommonFragments : Computes the number of common unique fragments between molecules;

"CommonFragments(help)" shows internal options

* CommonFragmentsTanimoto : Computes the tanimoto coefficient of the (#shared / #total)the number of

common unique fragments between molecules; "CommonFragmentsTanimoto(help)" shows internal options

* Dihedral : Calculates a function of the differences between all dihedral angles in the molecule;

"Dihedral(help)" shows internal options

* DihedralBins : Compares dihedral bin strings of two molecules to determine whether they have

essentially the same dihedral angles; "DihedralBins(help)" shows internal options

* LargestCommonDisconnectedSubstructureMetric : Computes the tanimoto coefficient of the largest

common (possibly disconnected) substructure between two molecules.;

"LargestCommonDisconnectedSubstructureMetric(help)" shows internal options

* LargestCommonDisconnectedSubstructureSize : Computes largest common (possibly disconnected)

substructure between two molecules.; "LargestCommonDisconnectedSubstructureSize(help)" shows internal

options

* LargestCommonDisconnectedSubstructureTanimoto : Computes the tanimoto coefficient of the largest

common (possibly disconnected) substructure between two molecules.;

"LargestCommonDisconnectedSubstructureTanimoto(help)" shows internal options

* LargestCommonSubstructureMetric : Computes the tanimoto coefficient of the largest common

connected substructure between two molecules.; "LargestCommonSubstructureMetric(help)" shows internal

options

* LargestCommonSubstructureSize : Computes largest common connected substructure between two

molecules.; "LargestCommonSubstructureSize(help)" shows internal options

* LargestCommonSubstructureTanimoto : Computes the tanimoto coefficient of the largest common

connected substructure between two molecules.; "LargestCommonSubstructureTanimoto(help)" shows internal

options

* LigFit : Orients a small molecule in a pocket cavity by minimizing geometric overlap of matched

atoms; "LigFit(help)" shows internal options

* MolAlignByParts : Aligns and computes property RMSD for molecule conformer libraries by parts;

"MolAlignByParts(help)" shows internal options

* MultiAlign : Align multiple molecules simultaneously; "MultiAlign(help)" shows internal options

* PropertyDistance : Calculates the root-mean-squared-deviation between properties between

molecules; "PropertyDistance(help)" shows internal options

* PropertyFieldDistance : Computes distance or correlation between molecular fields;

"PropertyFieldDistance(help)" shows internal options

* PropertyRMSDX : Calculates the root-mean-squared-deviation between positions and properties

between molecules using the formula sqrt( (x1-x2)^2 + (y1-y2)^2 + (z1-z2)^2 + (p1-p2)^2), where p

refers to the property value. The RMSD is then divided by 1 + a * ln( n-atoms-aligned mol / base).

a is set such that if only 1 atom aligns, the denominator evaluates to 1/base (e.g. a =

(base-1)/(base*ln(base)))Alignment assignments are removed, in increasing order of RMSD, to

optimize this function. The default property and property weight scheme corresponds to the

ChargeRMSD metric reported in Gregory et al., ACS Chemical Neuroscience. PMID: 24528109;

"PropertyRMSDX(help)" shows internal options

* PropertyTanimotoDistance : Calculates the Tanimoto/Jaccard distance between properties between

molecules; "PropertyTanimotoDistance(help)" shows internal options

* PsiField : Aligns two molecules and finds best property RMSD based on

ConformationComparisonPropertyFieldCorrelation scoring.; "PsiField(help)" shows internal options

* PsiFlexField : Aligns and computes property RMSD for molecule conformer libraries;

"PsiFlexField(help)" shows internal options

* SymmetryRMSD : Calculates the root-mean-squared-deviation between two molecules that are identical

onthe constitutional level, and whose atoms have already been aligned; "SymmetryRMSD(help)" shows internal

options

* SymmetryRealSpaceRMSD : Calculates the root-mean-squared-deviation between two molecules that are

identical onthe constitutional level, and whose atoms have already been aligned;

"SymmetryRealSpaceRMSD(help)" shows internal options

* NAromaticRingHalogensMaxFragment : Determines the sum of the number of halogen substituents on aromatic rings in

a molecule. Alternatively, identifies the aromatic ring with the most number of halogens and returns that count.

Parameter: <allowed_halogens> halogen types that will be counted, default: "F Cl Br I", any string

* NAromaticRingHalogensTotal : Determines the sum of the number of halogen substituents on aromatic rings in a

molecule. Alternatively, identifies the aromatic ring with the most number of halogens and returns that count.

Parameter: <allowed_halogens> halogen types that will be counted, default: "F Cl Br I", any string

* PCC-AD-DNN : Calculates binding affinity (in units of pKd) weighted by 1.0 minus the model applicability domain

score

Default label : PCC-AD-DNN(misc property id=bcl_pocket_id_080,receptor filename="")

Parameters:

<misc property id> misc property name of corresponding protein binding pocket for current molecule,

default: "bcl_pocket_id_080", any string

<receptor filename> the filename of the protein binding pocket of interest, default: "", any string

* PCC-DNN : Calculates binding affinity (in units of pKd)

Default label : PCC-DNN(misc property id=bcl_pocket_id_080,receptor filename="")

Parameters:

<misc property id> misc property name of corresponding protein binding pocket for current molecule,

default: "bcl_pocket_id_080", any string

<receptor filename> the filename of the protein binding pocket of interest, default: "", any string

* ReactionStructureSearch : Searches for specified substructures within the query molecule. Returns 1 if a

structure exists in the query molecule

Parameter: <filename> File containing molecules to compare with each query molecule, any existent file

* StructureCount : Searches for specified substructures within the query molecule. Returns the number of

substructure matches that exist in the query molecule

Default label : StructureCount(filename="",atom comparison=AtomType,bond

comparison=BondOrderAmideOrAromaticWithRingness,ignore h=1)

Parameters:

<filename> File containing molecules to compare with each query molecule, any existent file

<atom comparison> atom data that is compared to determine whether atoms are equivalent, default: "AtomType",

Allowed values: {Identity, ElementType, AtomType, AtomTypeAndChirality, AtomTypeAndComplexRingChirality,

AtomTypeAndSymmetry, AtomTypeAndHasSymmetry, AtomTypeAndNumberHydrogens, AtomTypeAndNumberHydrogensOnRingAtoms,

AtomTypeAndNumberHydrogensOnRingsAndDistinguishHydrogens, AtomTypeAndDistinguishHydrogens, CIPPriorityHighToLow,

CouldHaveSubstituents}

<bond comparison> bond data that is compared, default: "BondOrderAmideOrAromaticWithRingness",

Allowed values: {Identity, BondOrder, NumberOfElectrons, Conjugation, IsConjugated, IsAromatic, IsAmide,

IsInRing, BondOrderInRingOrAromatic, BondOrderOrAromatic, BondOrderAmideOrAromatic,

BondOrderOrAromaticWithRingness, BondOrderAmideOrAromaticWithRingness,

FuzzyBondOrderWithIsometryOrAromaticWithRingness, FuzzyBondOrderAmideOrAromaticWithRingness,

ConstitutionalBondType, BondOrderWithIsometry, Isometry, IsIsometric, BondOrderWithIsometryOrAromatic,

BondOrderAmideWithIsometryOrAromaticWithRingness, ConfigurationalBondType}

<ignore h> true to ignore hydrogens when comparing molecules, default: "True", Any non-negative integer

* StructureSearch : Searches for specified substructures within the query molecule. Returns 1 if a structure exists

in the query molecule

Default label : StructureSearch(filename="",atom comparison=AtomType,bond

comparison=BondOrderAmideOrAromaticWithRingness,ignore h=1)

Parameters:

<filename> File containing molecules to compare with each query molecule, any existent file

<atom comparison> atom data that is compared to determine whether atoms are equivalent, default: "AtomType",

Allowed values: {Identity, ElementType, AtomType, AtomTypeAndChirality, AtomTypeAndComplexRingChirality,

AtomTypeAndSymmetry, AtomTypeAndHasSymmetry, AtomTypeAndNumberHydrogens, AtomTypeAndNumberHydrogensOnRingAtoms,

AtomTypeAndNumberHydrogensOnRingsAndDistinguishHydrogens, AtomTypeAndDistinguishHydrogens, CIPPriorityHighToLow,

CouldHaveSubstituents}

<bond comparison> bond data that is compared, default: "BondOrderAmideOrAromaticWithRingness",

Allowed values: {Identity, BondOrder, NumberOfElectrons, Conjugation, IsConjugated, IsAromatic, IsAmide,

IsInRing, BondOrderInRingOrAromatic, BondOrderOrAromatic, BondOrderAmideOrAromatic,

BondOrderOrAromaticWithRingness, BondOrderAmideOrAromaticWithRingness,

FuzzyBondOrderWithIsometryOrAromaticWithRingness, FuzzyBondOrderAmideOrAromaticWithRingness,

ConstitutionalBondType, BondOrderWithIsometry, Isometry, IsIsometric, BondOrderWithIsometryOrAromatic,

BondOrderAmideWithIsometryOrAromaticWithRingness, ConfigurationalBondType}

<ignore h> true to ignore hydorgens when comparing molecules, default: "True", Any non-negative integer

* Template : Create a descriptor template; which is a partial definition for a descriptor, with remaining

user-defined arguments that can be set later

Default label : Template(signature="","")

Parameters:

<signature> Signature of the descriptor template, e.g. 3DA12(X). Parameters of the signature become (anonymous)

arguments whenever the template is called, so the template can be called with 3DA12(Atom_Identity), any object serialization tree

Definition of the descriptor template. Each argument in the signature should normally appear in the definition,

e.g. Template(signature=3DA12(X),3DA(property=X,steps=48,step size=0.25,temperature=100)), any object serialization tree

* UMol2D : calculates the number of certain types of atom environments

Default label : UMol2D(atom hashing type=Atom,feature size=574,Atom environment height=1)

Parameters:

<atom hashing type> Choose one of two: Element, Atom. Atom by default, default: "Atom",

Allowed values: {Element, Atom}

<feature size> get the feature size under the normal dimension setting. Range: 1-250 for Element(height=1), 1-574

for Atom(height=1), 1-5117 for Element(height=2), 1-8080 for Atom(height=2)Default: 574, default: "574", Any non-negative integer

<Atom environment height> Number of bond sphere from the center atom Range: 1 or 2Default: 1, default: "1",

Any non-negative integer

* UniformRandom : Returns a uniform-randomly chosen set of values

Parameter: <size> number of values to return per Molecule, Any non-negative integer

* UniformRandomDuplicated : Returns a uniform-randomly chosen value, copied {size} times

Parameter: <size> number of values to return per Molecule, Any non-negative integer

Operations

* MoleculeMax : Returns the max of the given descriptor across a Molecule

(anonymous) parameter: The descriptor to compute the max of across the Molecule,

choose any implementation of Molecule/Atom Numeric Descriptor (already listed)

* MoleculeMean : Returns the mean of the given descriptor across a Molecule

(anonymous) parameter: The descriptor to compute the mean of across the Molecule,

choose any implementation of Molecule/Atom Numeric Descriptor (already listed)

* MoleculeMin : Returns the min of the given descriptor across a Molecule

(anonymous) parameter: The descriptor to compute the min of across the Molecule,

choose any implementation of Molecule/Atom Numeric Descriptor (already listed)

* MoleculeRange : Returns the range of the given descriptor across a Molecule

(anonymous) parameter: The descriptor to compute the range of across the Molecule,

choose any implementation of Molecule/Atom Numeric Descriptor (already listed)

* MoleculeStandardDeviation : Returns the standarddeviation of the given descriptor across a Molecule

(anonymous) parameter: The descriptor to compute the standarddeviation of across the Molecule,

choose any implementation of Molecule/Atom Numeric Descriptor (already listed)

* MoleculeSum : Returns the sum of the given descriptor across a Molecule

(anonymous) parameter: The descriptor to compute the sum of across the Molecule,

choose any implementation of Molecule/Atom Numeric Descriptor (already listed)

* MoleculeWeightedMean : mean of a descriptor across the Molecule, weighted by any other descriptor

Default label : MoleculeWeightedMean(Molecule/Atom Numeric Descriptor,weight=Molecule/Atom Numeric Descriptor)

Parameters:

The descriptor to compute the mean of across the Molecule,

choose any implementation of Molecule/Atom Numeric Descriptor (already listed)

<weight> The descriptor used to weight the primary descriptor,

choose any implementation of Molecule/Atom Numeric Descriptor (already listed)

* MoleculeWeightedStandardDeviation : standarddeviation of a descriptor across the Molecule, weighted by any other

descriptor

Default label : MoleculeWeightedStandardDeviation(Molecule/Atom Numeric Descriptor,weight=Molecule/Atom Numeric

Descriptor)

Parameters:

The descriptor to compute the standarddeviation of across the Molecule,

choose any implementation of Molecule/Atom Numeric Descriptor (already listed)

<weight> The descriptor used to weight the primary descriptor,

choose any implementation of Molecule/Atom Numeric Descriptor (already listed)

* MoleculeWeightedSum : sum of a descriptor across the Molecule, weighted by any other descriptor

Default label : MoleculeWeightedSum(Molecule/Atom Numeric Descriptor,weight=Molecule/Atom Numeric Descriptor)

Parameters:

The descriptor to compute the sum of across the Molecule,

choose any implementation of Molecule/Atom Numeric Descriptor (already listed)

<weight> The descriptor used to weight the primary descriptor,

choose any implementation of Molecule/Atom Numeric Descriptor (already listed)

* ShapeMoments : computes the mean, std, and skew of atomic distances from four anchor points: the molecular

centroid (mct), closest atom to the mct (cam), furthest atom from mct (fam), and furthest atom from fam (faf). The

molecular centroid is the center of the positions, weighted by the anchor property.

Default label : ShapeMoments(anchor=Molecule/Atom Numeric Descriptor,weighting=Molecule/Atom Numeric Descriptor)

Parameters:

<anchor> weighting property used to define the centroid and other anchor points in the target molecule,

choose any implementation of Molecule/Atom Numeric Descriptor (already listed)

<weighting> weighting for statistical moments; e.g. use Atom_Polarizability to place more weight on distances

associated with highly polar species in the molecule, choose any implementation of Molecule/Atom Numeric Descriptor (already listed)

Customizable Operations

* 2DAMax : computes max for each bin of the 2DA of a specified atom property

Default label : 2DAMax(steps=12,property=Molecule/Atom Numeric Descriptor,substitution_value=nan)

Parameters:

<steps> # of steps; corresponds to maximum # of bonds between atoms that will be considered for autocorrelation,

default: "11", Any non-negative integer >= 1 <= 1000000

<property> property over which to calculate the 2D-autocorrelation, default: "Atom_Identity",

choose any implementation of Molecule/Atom Numeric Descriptor (already listed)

<substitution_value> value to replace empty bins unless given a nan, then uses worst found value,

default: "nan", Any decimal (floating-point) value

* 2DAMaxSign : computes 2D (bond distance) autocorrelation of a specified atom property

Default label : 2DAMaxSign(property=Molecule/Atom Numeric Descriptor,steps=12)

Parameters:

<property> property over which to calculate the smooth radial distribution function,

choose any implementation of Molecule/Atom Numeric Descriptor (already listed)

<steps> # of steps/bins (each of size = step size) used in the radial distribution function, default: "48",

Any non-negative integer >= 1 <= 1000000

* 2DAMin : computes min for each bin of the 2DA of a specified atom property

Default label : 2DAMin(steps=12,property=Molecule/Atom Numeric Descriptor,substitution_value=nan)

Parameters:

<steps> # of steps; corresponds to maximum # of bonds between atoms that will be considered for autocorrelation,

default: "11", Any non-negative integer >= 1 <= 1000000

<property> property over which to calculate the 2D-autocorrelation, default: "Atom_Identity",

choose any implementation of Molecule/Atom Numeric Descriptor (already listed)

<substitution_value> value to replace empty bins unless given a nan, then uses worst found value,

default: "nan", Any decimal (floating-point) value

* 3DAClosestPairRealSpace : Relates the autocorrelations of two independent molecules

Default label : 3DAClosestPairRealSpace(property=Molecule/Atom Numeric Descriptor,step

size=0.5,steps=14,temperature=5,gaussian=1,interpolate=1,misc property

id="",reference_conformer="",mol_a_atom_indices_filename="",mol_b_atom_indices_filename="")

Parameters:

<property> property over which to calculate the function,

choose any implementation of Molecule/Atom Numeric Descriptor (already listed)

<step size> size of each step in angstroms, default: "0.50", Any decimal (floating-point) value >= 0.01 <= 100

<steps> # of steps/bins (each of size = step size) used in the function, default: "14",

Any non-negative integer >= 1 <= 1000000

<temperature> increasing temperature spreads autocorrelation across more distant bins, default: "5.0",

Any decimal (floating-point) value >= 0 <= 1000

<gaussian> whether to apply gaussian smoothing to the final curve. If set to false, temperature is ignored,

interpolation is linear, and no gaussian smoothing is performed, default: "True", Any non-negative integer

<interpolate> whether to interpolate values to the two nearest points; if false, all weight will be applied to

the nearest bin, default: "True", Any non-negative integer

<misc property id> misc property name of corresponding protein binding pocket for current molecule, default: "",

any string

<reference_conformer> the molecule (e.g. protein binding pocket) whose autocorrelation is being compared against

the input molecule descriptor set, default: "", any string

<mol_a_atom_indices_filename> get the atom indices from molecule A contributing to the 3da interaction and output

to this file; recommended to only use this for discrete properties, e.g.

Partial(3DAClosestPairRSAsym050(Multiply(Atom_HydrophobicTernary,Atom_Polarizability),Atom_IsInAromaticRingTernary),indices(23)),default: "",

any string

<mol_b_atom_indices_filename> get the atom indices from molecule B contributing to the 3da interaction and output

to this file; recommended to only use this for discrete properties, e.g.

Partial(3DAClosestPairRSAsym050(Multiply(Atom_HydrophobicTernary,Atom_Polarizability),Atom_IsInAromaticRingTernary),indices(23)),default: "",

any string

* 3DAClosestPairRealSpaceAsymmetry : Relates the autocorrelations of two independent molecules

Default label : 3DAClosestPairRealSpaceAsymmetry(property_a=Molecule/Atom Numeric

Descriptor,property_b=Molecule/Atom Numeric Descriptor,step

size=0.5,steps=14,temperature=5,gaussian=1,interpolate=1,misc property

id="",reference_conformer="",mol_a_atom_indices_filename="",mol_b_atom_indices_filename="")

Parameters:

<property_a> small molecule property,

choose any implementation of Molecule/Atom Numeric Descriptor (already listed)

<property_b> protein pocket property,

choose any implementation of Molecule/Atom Numeric Descriptor (already listed)

<step size> size of each step in angstroms, default: "0.50", Any decimal (floating-point) value >= 0.01 <= 100

<steps> # of steps/bins (each of size = step size) used in the function, default: "14",

Any non-negative integer >= 1 <= 1000000

<temperature> increasing temperature spreads autocorrelation across more distant bins, default: "5.0",

Any decimal (floating-point) value >= 0 <= 1000

<gaussian> whether to apply gaussian smoothing to the final curve. If set to false, temperature is ignored,

interpolation is linear, and no gaussian smoothing is performed, default: "True", Any non-negative integer

<interpolate> whether to interpolate values to the two nearest points; if false, all weight will be applied to

the nearest bin, default: "True", Any non-negative integer

<misc property id> misc property name of corresponding protein binding pocket for current molecule, default: "",

any string

<reference_conformer> the molecule (e.g. protein binding pocket) whose autocorrelation is being compared against

the input molecule descriptor set, default: "", any string

<mol_a_atom_indices_filename> get the atom indices from molecule A contributing to the 3da interaction and output

to this file; recommended to only use this for discrete properties, e.g.

Partial(3DAPairRSAsym050(Multiply(Atom_HydrophobicTernary,Atom_Polarizability),Atom_IsInAromaticRingTernary),indices(23)),default: "",

any string

<mol_b_atom_indices_filename> get the atom indices from molecule B contributing to the 3da interaction and output

to this file; recommended to only use this for discrete properties, e.g.

Partial(3DAPairRSAsym050(Multiply(Atom_HydrophobicTernary,Atom_Polarizability),Atom_IsInAromaticRingTernary),indices(23)),default: "",

any string

* 3DAPairConvolution : Relates the autocorrelations of two independent molecules

Default label : 3DAPairConvolution(property=Molecule/Atom Numeric Descriptor,steps=20,step size=0.5,window

size=4,weighting=bcl::descriptor::WindowWeightingInterface,misc property id="",reference_conformer="")

Parameters:

<property> property over which to calculate the function,

choose any implementation of Molecule/Atom Numeric Descriptor (already listed)

<steps> # of steps/bins (each of size = step size) used in the function, default: "20",

Any non-negative integer >= 1 <= 1000000

<step size> size of each step in angstroms, default: "0.50", Any decimal (floating-point) value >= 0.01 <= 100

<window size> size of each step in multiples of step size, default: "4.0", Any non-negative integer

<weighting> method of generating weights for the windowing function, default: "Hann",

choose any implementation of bcl::descriptor::WindowWeightingInterface (already listed)

<misc property id> misc property name of corresponding protein binding pocket for current molecule, default: "",

any string

<reference_conformer> the molecule (e.g. protein binding pocket) whose autocorrelation is being compared against

the input molecule descriptor set, default: "", any string

* 3DAPairConvolutionAsymmetry : Relates the autocorrelations of two independent molecules

Default label : 3DAPairConvolutionAsymmetry(property_a=Molecule/Atom Numeric Descriptor,property_b=Molecule/Atom

Numeric Descriptor,step size=0.5,window size=4,steps=20,weighting=bcl::descriptor::WindowWeightingInterface,misc

property id="",reference_conformer="")

Parameters:

<property_a> ligand property over which to calculate the function,

choose any implementation of Molecule/Atom Numeric Descriptor (already listed)

<property_b> pocket property over which to calculate the function,

choose any implementation of Molecule/Atom Numeric Descriptor (already listed)

<step size> size of each step in angstroms, default: "0.50", Any decimal (floating-point) value >= 0.01 <= 100

<window size> size of each step in multiples of step size, default: "4.0", Any non-negative integer

<steps> # of steps/bins (each of size = step size) used in the function, default: "20",

Any non-negative integer >= 1 <= 1000000

<weighting> method of generating weights for the windowing function,

choose any implementation of bcl::descriptor::WindowWeightingInterface (already listed)

<misc property id> misc property name of corresponding protein binding pocket for current molecule, default: "",

any string

<reference_conformer> the molecule (e.g. protein binding pocket) whose autocorrelation is being compared against

the input molecule descriptor set, default: "", any string

* 3DAPairRealSpace : Relates the autocorrelations of two independent molecules

Default label : 3DAPairRealSpace(property=Molecule/Atom Numeric Descriptor,step

size=0.5,steps=14,temperature=5,gaussian=1,interpolate=1,misc property

id="",reference_conformer="",mol_a_atom_indices_filename="",mol_b_atom_indices_filename="")

Parameters:

<property> property over which to calculate the function,

choose any implementation of Molecule/Atom Numeric Descriptor (already listed)

<step size> size of each step in angstroms, default: "0.50", Any decimal (floating-point) value >= 0.01 <= 100

<steps> # of steps/bins (each of size = step size) used in the function, default: "14",

Any non-negative integer >= 1 <= 1000000

<temperature> increasing temperature spreads autocorrelation across more distant bins, default: "5.0",

Any decimal (floating-point) value >= 0 <= 1000

<gaussian> whether to apply gaussian smoothing to the final curve. If set to false, temperature is ignored,

interpolation is linear, and no gaussian smoothing is performed, default: "True", Any non-negative integer

<interpolate> whether to interpolate values to the two nearest points; if false, all weight will be applied to

the nearest bin, default: "True", Any non-negative integer

<misc property id> misc property name of corresponding protein binding pocket for current molecule, default: "",

any string

<reference_conformer> the molecule (e.g. protein binding pocket) whose autocorrelation is being compared against

the input molecule descriptor set, default: "", any string

<mol_a_atom_indices_filename> get the atom indices from molecule A contributing to the 3da interaction and output

to this file; recommended to only use this for discrete properties, e.g.

Partial(3DAPairRSAsym050(Multiply(Atom_HydrophobicTernary,Atom_Polarizability),Atom_IsInAromaticRingTernary),indices(23)),default: "",

any string

<mol_b_atom_indices_filename> get the atom indices from molecule B contributing to the 3da interaction and output

to this file; recommended to only use this for discrete properties, e.g.

Partial(3DAPairRSAsym050(Multiply(Atom_HydrophobicTernary,Atom_Polarizability),Atom_IsInAromaticRingTernary),indices(23)),default: "",

any string

* 3DAPairRealSpaceAsymmetry : Relates the autocorrelations of two independent molecules

Default label : 3DAPairRealSpaceAsymmetry(property_a=Molecule/Atom Numeric Descriptor,property_b=Molecule/Atom

Numeric Descriptor,step size=0.5,steps=14,temperature=5,gaussian=1,interpolate=1,misc property

id="",reference_conformer="",mol_a_atom_indices_filename="",mol_b_atom_indices_filename="")

Parameters:

<property_a> small molecule property,

choose any implementation of Molecule/Atom Numeric Descriptor (already listed)

<property_b> protein pocket property,

choose any implementation of Molecule/Atom Numeric Descriptor (already listed)

<step size> size of each step in angstroms, default: "0.50", Any decimal (floating-point) value >= 0.01 <= 100

<steps> # of steps/bins (each of size = step size) used in the function, default: "14",

Any non-negative integer >= 1 <= 1000000

<temperature> increasing temperature spreads autocorrelation across more distant bins, default: "5.0",

Any decimal (floating-point) value >= 0 <= 1000

<gaussian> whether to apply gaussian smoothing to the final curve. If set to false, temperature is ignored,

interpolation is linear, and no gaussian smoothing is performed, default: "True", Any non-negative integer

<interpolate> whether to interpolate values to the two nearest points; if false, all weight will be applied to

the nearest bin, default: "True", Any non-negative integer

<misc property id> misc property name of corresponding protein binding pocket for current molecule, default: "",

any string

<reference_conformer> the molecule (e.g. protein binding pocket) whose autocorrelation is being compared against

the input molecule descriptor set, default: "", any string

<mol_a_atom_indices_filename> get the atom indices from molecule A contributing to the 3da interaction and output

to this file; recommended to only use this for discrete properties, e.g.

Partial(3DAPairRSAsym050(Multiply(Atom_HydrophobicTernary,Atom_Polarizability),Atom_IsInAromaticRingTernary),indices(23)),default: "",

any string

<mol_b_atom_indices_filename> get the atom indices from molecule B contributing to the 3da interaction and output

to this file; recommended to only use this for discrete properties, e.g.

Partial(3DAPairRSAsym050(Multiply(Atom_HydrophobicTernary,Atom_Polarizability),Atom_IsInAromaticRingTernary),indices(23)),default: "",

any string

* 3DAPairRealSpaceConvolution : Relates the autocorrelations of two independent molecules

Default label : 3DAPairRealSpaceConvolution(property=Molecule/Atom Numeric Descriptor,steps=20,step

size=0.5,window size=4,weighting=bcl::descriptor::WindowWeightingInterface,misc property

id="",reference_conformer="",cutoff=1.09012e+27)

Parameters:

<property> property over which to calculate the function,

choose any implementation of Molecule/Atom Numeric Descriptor (already listed)

<steps> # of steps/bins (each of size = step size) used in the function, default: "20",

Any non-negative integer >= 1 <= 1000000

<step size> size of each step in angstroms, default: "0.50", Any decimal (floating-point) value >= 0.01 <= 100

<window size> size of each step in multiples of step size, default: "4.0", Any non-negative integer

<weighting> method of generating weights for the windowing function, default: "Hann",

choose any implementation of bcl::descriptor::WindowWeightingInterface (already listed)

<misc property id> misc property name of corresponding protein binding pocket for current molecule, default: "",

any string

<reference_conformer> the molecule (e.g. protein binding pocket) whose autocorrelation is being compared against

the input molecule descriptor set, default: "", any string

<cutoff> distance cutoff (in Angstroms) for neighbor atom determination, default: "7.0",

Any decimal (floating-point) value

* 3DAPairRealSpaceConvolutionAsymmetry : Relates the autocorrelations of two independent molecules

Default label : 3DAPairRealSpaceConvolutionAsymmetry(property_a=Molecule/Atom Numeric

Descriptor,property_b=Molecule/Atom Numeric Descriptor,step size=0.5,window

size=4,steps=20,weighting=bcl::descriptor::WindowWeightingInterface,misc property

id="",reference_conformer="",cutoff=0)

Parameters:

<property_a> ligand property over which to calculate the function,

choose any implementation of Molecule/Atom Numeric Descriptor (already listed)

<property_b> pocket property over which to calculate the function,

choose any implementation of Molecule/Atom Numeric Descriptor (already listed)

<step size> size of each step in angstroms, default: "0.50", Any decimal (floating-point) value >= 0.01 <= 100

<window size> size of each step in multiples of step size, default: "4.0", Any non-negative integer

<steps> # of steps/bins (each of size = step size) used in the function, default: "20",

Any non-negative integer >= 1 <= 1000000

<weighting> method of generating weights for the windowing function,

choose any implementation of bcl::descriptor::WindowWeightingInterface (already listed)

<misc property id> misc property name of corresponding protein binding pocket for current molecule, default: "",

any string

<reference_conformer> the molecule (e.g. protein binding pocket) whose autocorrelation is being compared against

the input molecule descriptor set, default: "", any string

<cutoff> distance cutoff (in Angstroms) for neighbor atom determination, default: "7.0",

Any decimal (floating-point) value

* 3dDistribution : computes the smooth radial distribution function using a given atom property

Default label : 3dDistribution(property=Molecule/Atom Numeric Descriptor,center property=Molecule/Atom Numeric

Descriptor,step size=1,steps=12)

Parameters:

<property> property over which to calculate the smooth radial distribution function,

choose any implementation of Molecule/Atom Numeric Descriptor (already listed)

<center property> property used to weight the atom distances. Should usually be something that is always >= 0,

choose any implementation of Molecule/Atom Numeric Descriptor (already listed)

<step size> size of each step in angstroms, default: "0.25", Any decimal (floating-point) value >= 0.01 <= 100

<steps> # of steps/bins (each of size = step size) used in the radial distribution function, default: "48",

Any non-negative integer >= 1 <= 1000000

* 3dDistributionSign : computes the smooth radial distribution function using a given atom property

Default label : 3dDistributionSign(property=Molecule/Atom Numeric Descriptor,center property=Molecule/Atom

Numeric Descriptor,step size=1,steps=12)

Parameters:

<property> property over which to calculate the smooth radial distribution function,

choose any implementation of Molecule/Atom Numeric Descriptor (already listed)

<center property> property used to weight the atom distances. Should usually be something that is always >= 0,

choose any implementation of Molecule/Atom Numeric Descriptor (already listed)

<step size> size of each step in angstroms, default: "0.25", Any decimal (floating-point) value >= 0.01 <= 100

<steps> # of steps/bins (each of size = step size) used in the radial distribution function, default: "48",

Any non-negative integer >= 1 <= 1000000

* 3daSoftMax : computes the smooth radial distribution function using a given atom property

Default label : 3daSoftMax(property=Molecule/Atom Numeric Descriptor,step

size=1,temperature=100,steps=12,gaussian=1)

Parameters:

<property> property over which to calculate the smooth radial distribution function,

choose any implementation of Molecule/Atom Numeric Descriptor (already listed)

<step size> size of each step in angstroms, default: "0.25", Any decimal (floating-point) value >= 0.01 <= 100

<temperature> increasing temperature spreads autocorrelation across more distant bins, default: "100",

Any decimal (floating-point) value >= 0 <= 1000

<steps> # of steps/bins (each of size = step size) used in the radial distribution function, default: "48",

Any non-negative integer >= 1 <= 1000000

<gaussian> whether to apply gaussian smoothing to the final curve. If set to false, temperature is ignored,

interpolation is linear, and no gaussian smoothing is performed, default: "True", Any non-negative integer

* 3daSoftMaxSign : computes the smooth radial distribution function using a given atom property

Default label : 3daSoftMaxSign(property=Molecule/Atom Numeric Descriptor,step

size=1,temperature=100,steps=12,gaussian=1)

Parameters:

<property> property over which to calculate the smooth radial distribution function,

choose any implementation of Molecule/Atom Numeric Descriptor (already listed)

<step size> size of each step in angstroms, default: "0.25", Any decimal (floating-point) value >= 0.01 <= 100

<temperature> increasing temperature spreads autocorrelation across more distant bins, default: "100",

Any decimal (floating-point) value >= 0 <= 1000

<steps> # of steps/bins (each of size = step size) used in the radial distribution function, default: "48",

Any non-negative integer >= 1 <= 1000000

<gaussian> whether to apply gaussian smoothing to the final curve. If set to false, temperature is ignored,

interpolation is linear, and no gaussian smoothing is performed, default: "True", Any non-negative integer

* 3daSoftMin : computes the smooth radial distribution function using a given atom property

Default label : 3daSoftMin(property=Molecule/Atom Numeric Descriptor,step

size=1,temperature=100,steps=12,gaussian=1)

Parameters:

<property> property over which to calculate the smooth radial distribution function,

choose any implementation of Molecule/Atom Numeric Descriptor (already listed)

<step size> size of each step in angstroms, default: "0.25", Any decimal (floating-point) value >= 0.01 <= 100

<temperature> increasing temperature spreads autocorrelation across more distant bins, default: "100",

Any decimal (floating-point) value >= 0 <= 1000

<steps> # of steps/bins (each of size = step size) used in the radial distribution function, default: "48",

Any non-negative integer >= 1 <= 1000000

<gaussian> whether to apply gaussian smoothing to the final curve. If set to false, temperature is ignored,

interpolation is linear, and no gaussian smoothing is performed, default: "True", Any non-negative integer

* Central2DASign : Measure 2DASign of property at variable distances from molecule topological center

Default label : Central2DASign(property=Molecule/Atom Numeric

Descriptor,max_bonds_from_center=12,2da_steps=12,smooth=1,temperature=100)

Parameters:

<property> property over which to calculate the smooth radial distribution function,

choose any implementation of Molecule/Atom Numeric Descriptor (already listed)

<max_bonds_from_center> size of outer array containing each signed 2DA, default: "12",

Any non-negative integer >= 1 <= 1000000

<2da_steps> # of steps/bins (each of size = step size) used in the radial distribution function, default: "12",

Any non-negative integer >= 1 <= 1000000

<smooth> whether to smooth the 2DA, default: "1", Any non-negative integer

<temperature> increasing temperature spreads autocorrelation across more distant bins, default: "100",

Any decimal (floating-point) value >= 0 <= 1000

* CoulombicForce : computes the intramolecular coulombic force (without the dielectric constant)

Default label : CoulombicForce(Molecule/Atom Numeric Descriptor,distance cutoff=nan,non-covalent=0)

Parameters:

charge property, choose any implementation of Molecule/Atom Numeric Descriptor (already listed)

<distance cutoff> maximum distance to consider. forces will be scaled (using a cosine transition) from 1A prior

to this value, default: "nan", Any decimal (floating-point) value

<non-covalent> true to ignore covalently bonded neighbors of an atom for the force calculation,

default: "False", Any non-negative integer

* MolecularAsymmetry : Calculates an rdf-like asymmetry vector for the molecule based on overall distribution of

atoms and/or properties. Use of this descriptor must be cited as: Sliwoski, Gregory, et al. "BCL::

EMAS—Enantioselective Molecular Asymmetry Descriptor for 3D-QSAR." Molecules 17.8 (2012): 9971-9989.

Link: www.http://meilerlab.org/index.php/publications/show/2012

Default label : MolecularAsymmetry(property=Molecule/Atom Numeric Descriptor,step

size=0,temperature=0,steps=0,sum properties=0)

Parameters:

<property> property over which to calculate the molecular asymmetry score,

choose any implementation of Molecule/Atom Numeric Descriptor (already listed)

<step size> size of each step in angstroms, default: "0.1", Any decimal (floating-point) value >= 0.01 <= 100

<temperature> increasing temperature spreads intensity across more distant bins, default: "100",

Any decimal (floating-point) value >= 0 <= 1000

<steps> # of steps/bins (each of size = step size) used in the radial distribution function, default: "24",

Any non-negative integer >= 1 <= 1000000

<sum properties> Use summation method to weight properties, Any non-negative integer

* MolecularFragmentRescale : Statistics of a descriptor across the series of fragments split off the original

molecule

Default label : MolecularFragmentRescale(Molecule/Atom Numeric

Descriptor,splitter=bcl::chemistry::FragmentSplitInterface,rescale="",cache=1)

Parameters:

The descriptor to compute the statistics of across the series of fragments,

choose any implementation of Molecule/Atom Numeric Descriptor (already listed)

<splitter> The method of splitting the molecule into a series of fragments,

choose any implementation of bcl::chemistry::FragmentSplitInterface:

Basic Implementations

* Chains : returns either ring or chains of a molecule

* GADDFragments : splits molecules into their GA-based Drug Database fragments (see

http://www.daylight.com/meetings/mug01/Yang/gadd/)

* InverseScaffold : returns the remaining components of a molecule after the murcko scaffold is

removed

* Isolate : splits a macromolecule into component parts

* Largest : splits a macromolecule and returns largest componenet

* Rigid : Rigid components; defined by breaking all single bonds that are not in a ring, amide, or

which connect to a terminal atom (disregarding H)

* RigidSansAmide : Rigid components; defined by breaking all single bonds that are not in a ring,

amide, or which connect to a terminal atom (disregarding H)

* Rings : returns either ring or chains of a molecule

* RingsWithUnsaturatedSubstituents : returns rings of a molecule, including unsaturated substituents

* Scaffolds : returns scaffolds that are present in the molecule of interest

* UnbridgedAromaticRings : returns either ring or chans of a molecule

* UnbridgedRings : returns either ring or chans of a molecule

Customizable Implementations

* ECFPFragments : splits molecules into fragments similar to those used for extended connectivity

fingerprints (see http://pubs.acs.org/doi/abs/10.1021/ci100050t); "ECFPFragments(help)" shows internal

options

* LargestCommonSubstructure : splits molecules into their common substructures relative to an input

set; "LargestCommonSubstructure(help)" shows internal options

* LinearFragments : Splits a molecule into a linear fragments (i.e. fragments that progress 1 atom

out at a time without branching). This implementation is similar to the openbabel FP2 fingerprint;

"LinearFragments(help)" shows internal options

* MoleculeFragmentRecombination : Recombine molecules based on maximum common substructure

differences; "MoleculeFragmentRecombination(help)" shows internal options

* SampleConformations : ; "SampleConformations(help)" shows internal options

<rescale> Rescalings to perform,

Container with at least 1 Allowed values: {MinMax, ZScore, SubtractMean, DivideMean}

<cache> Whether to cache conformation ensembles for each conformation, default: "1", Any non-negative integer

* MolecularFragmentStatistics : Statistics of a descriptor across the series of fragments split off the original

molecule

Default label : MolecularFragmentStatistics(Molecule/Atom Numeric Descriptor,weight=Molecule/Atom Numeric

Descriptor,splitter=bcl::chemistry::FragmentSplitInterface,statistics="")

Parameters:

The descriptor to compute the statistics of across the series of fragments,

choose any implementation of Molecule/Atom Numeric Descriptor (already listed)

<weight> Descriptor that can be defined to weight the values of the primary descriptor. Primarily used for

average and standard deviation calculations, but also influences Min and Max, because if the weight descriptor is

defined and is <= 0, the value is ignored for both min/max and ave/std, optional, choose any implementation of Molecule/Atom Numeric Descriptor (already listed)

<splitter> The method of splitting the molecule into a series of fragments,

choose any implementation of bcl::chemistry::FragmentSplitInterface (already listed)

<statistics> Statistics desired, Container with at least 1 Allowed values: {Min, Max, Mean, StDev, Sum}

* MolecularMaxFragmentStatistics : Statistics of a descriptor across the series of fragments split off the original

molecule

Default label : MolecularMaxFragmentStatistics(Molecule/Atom Numeric Descriptor,maximize=Molecule/Atom Numeric

Descriptor,splitter=bcl::chemistry::FragmentSplitInterface)

Parameters:

The descriptor to compute the statistics of across the series of fragments,

choose any implementation of Molecule/Atom Numeric Descriptor (already listed)

<maximize> Descriptor that can be defined to weight the values of the primary descriptor. Primarily used for

average and standard deviation calculations, but also influences Min and Max, because if the weight descriptor is

defined and is <= 0, the value is ignored for both min/max and ave/std, optional, choose any implementation of Molecule/Atom Numeric Descriptor (already listed)

<splitter> The method of splitting the molecule into a series of fragments,

choose any implementation of bcl::chemistry::FragmentSplitInterface (already listed)

* MoleculeHistogram1D : computes a histogram using a single descriptor

Default label : MoleculeHistogram1D(Molecule/Atom Numeric Descriptor,min=0,max=1,bin

size=1,smoothing=0,catchall=0)

Parameters:

descriptor to use, choose any implementation of Molecule/Atom Numeric Descriptor (already listed)

<min> min value to consider, Any decimal (floating-point) value

<max> max value to consider, Any decimal (floating-point) value

<bin size> size of each bin, Any decimal (floating-point) value >= 0

<smoothing> if non-zero, return a histogram with points smoothed with a gaussian kernel, specifically

Ae^(-BinDistance/SmoothingDistance), where BinDistance is the euclidean distance to center of the given bin, and

A is chosen such that the histogram's sum still equals 1, default: "0", Any decimal (floating-point) value >= 0

<catchall> If set, values that fall outside the boundaries will be placed into the nearest bin,

default: "False", Any non-negative integer

* RDFMaxSign : computes the smooth radial distribution function using a given atom property

Default label : RDFMaxSign(property=Molecule/Atom Numeric Descriptor,step size=0,temperature=0,steps=0)

Parameters:

<property> property over which to calculate the smooth radial distribution function,

choose any implementation of Molecule/Atom Numeric Descriptor (already listed)

<step size> size of each step in angstroms, default: "0.25", Any decimal (floating-point) value >= 0.01 <= 100

<temperature> increasing temperature spreads autocorrelation across more distant bins, default: "100",

Any decimal (floating-point) value >= 0 <= 1000

<steps> # of steps/bins (each of size = step size) used in the radial distribution function, default: "48",

Any non-negative integer >= 1 <= 1000000

* RotamerCoulombicForce : computes the intramolecular coulombic force (without the dielectric constant) due to the

rotamer chosen, thus ignoring covalent and 1,3 bonding interactions

Default label : RotamerCoulombicForce(Molecule/Atom Numeric Descriptor,distance cutoff=nan)

Parameters:

charge property, choose any implementation of Molecule/Atom Numeric Descriptor (already listed)

<distance cutoff> maximum distance to consider. forces will be scaled (using a cosine transition) from 1A prior

to this value, default: "nan", Any decimal (floating-point) value

* Triangulator : computes triangular autocorrelation of a specified atom property.This is much like RDF, but

considers all triplets of atoms.

Default label : Triangulator(property=Molecule/Atom Numeric Descriptor,step

size=0,cutoff=0,temperature=0,steps=0)

Parameters:

<property> property over which to calculate the triangular autocorrelation,

choose any implementation of Molecule/Atom Numeric Descriptor (already listed)

<step size> size of each step in angstroms, default: "1.0", Any decimal (floating-point) value >= 0.01 <= 100

<cutoff> max area (A^2) to consider; 0.0 to consider all areas, default: "0.0",

Any decimal (floating-point) value >= 0 <= 100

<temperature> increasing temperature spreads autocorrelation across more distant bins, default: "100",

Any decimal (floating-point) value >= 0 <= 1000

<steps> # of steps/bins (each of size = step size) used in the radial distribution function, default: "11",

Any non-negative integer >= 1 <= 1000000

*********************************************************************************************************************

********************************************** Descriptors of Atoms *************************************************

*********************************************************************************************************************

These can be converted into a Molecule-level descriptor using MoleculeSum(X), where X is any descriptor listed

below

Basic Implementations

* AtomMaxRingSize : Maximum size of a ring that this atom is part of. For atoms that are not in a ring, returns 2.

The size of the largest ring with no internal rings is returned for atoms that are part of ring systems

* AtomMinRingSize : Minimum size of a ring that this atom is part of. For atoms that are not in a ring, returns

10000.

* AtomTypeNumber : Retrieves the atomic number of each atom

* Atom_AdditiveAtomicPolarizability : Retrieves the AdditiveAtomicPolarizability of desired atom

* Atom_AromaticityAxes : For aromatic atoms, the axes of the aromatic field

* Atom_BoilingPoint : Retrieves the elemental BoilingPoint for each atom

* Atom_CovalentRadius : Retrieves the elemental CovalentRadius for each atom

* Atom_CovalentSurfaceArea : approximates the surface area of atoms using the covalent radius, considering overlap

from neighboring atoms. Alias for : Atom_SurfaceArea(radius=Atom_CovalentRadius,min radius=Molecule/Atom Numeric

Descriptor)

* Atom_CovalentVolume : approximates the volume of atoms using the covalent radius, considering overlap from

neighboring atoms. Alias for : Atom_Volume(radius=Atom_CovalentRadius,min radius=Molecule/Atom Numeric

Descriptor)

* Atom_EffectivePolarizability : atomic polarizability smoothed over molecule

* Atom_ElectroNegativity : Retrieves the elemental ElectroNegativity for each atom

* Atom_EstCovalentSurfaceArea : The surface area, estimated from atom type covalent radii

* Atom_EstVdwSurfaceArea : The surface area, estimated from atom type elemental van der waals radii

* Atom_EstVdwSurfaceAreaCSD : The surface area, estimated from atom type CSD-derived van der waals radii

* Atom_FormalCharge : the formal charge of each atom

* Atom_GyromagneticRatio : Retrieves the elemental GyromagneticRatio for each atom

* Atom_HSigmaChargeL : User-defined. Alias for : Multiply(Atom_SigmaChargeL,IsH)

* Atom_HVchargeL : User-defined. Alias for : Multiply(Atom_VchargeL,IsH)

* Atom_HbondAcceptors : 1 for hydrogen bond acceptors (N and O), 0 for other elements

* Atom_HbondDonors : 1 for hydrogen bond donors (NH and OH), 0 for others

* Atom_InAromaticRingIntersection : User-defined. Alias for :

GreaterEqual(lhs=BondTypeCount(property=IsAromatic,value=1),rhs=3)

* Atom_InRingIntersection : User-defined. Alias for :

GreaterEqual(lhs=BondTypeCount(property=IsInRing,value=1),rhs=3)

* Atom_IonizationPotential : Retrieves the elemental IonizationPotential for each atom

* Atom_IsInAromaticRing : User-defined. Alias for :

GreaterEqual(lhs=BondTypeCount(property=IsAromatic,value=1),rhs=2)

* Atom_IsSP : 1 for sp, -1 for non-sp non-terminal atoms, 0 for everything else

* Atom_IsSP2 : 1 for sp2, -1 for non-sp2 non-terminal atoms, 0 for everything else

* Atom_IsSP3 : 1 for sp3, -1 for non-sp3 non-terminal atoms, 0 for everything else

* Atom_LonePairEN : calculates lone-pair electronegativity, see

http://www.springerlink.com/content/w300520690302287/fulltext.pdf

* Atom_LonePairElectronAffinity : Retrieves the LonePairElectronAffinity of desired atom

* Atom_LonePairElectronegativity : Retrieves the LonePairElectronegativity of desired atom

* Atom_LonePairIonizationPotential : Retrieves the LonePairIonizationPotential of desired atom

* Atom_MainGroup : Retrieves the elemental MainGroup for each atom

* Atom_Mass : Retrieves the elemental Mass for each atom

* Atom_MeltingPoint : Retrieves the elemental MeltingPoint for each atom

* Atom_NeighborDirection : The unit vector formed by the relative positions of neighboring atoms

* Atom_NumberValences : the formal charge of each atom

* Atom_PiCharge : uses Hueckel matrix to determine pi-orbital partial charge

* Atom_PiEN : uses Hueckel matrix to determine pi-orbital electronegativity

* Atom_PiOrbitalElectronegativityMulliken : Retrieves the PiOrbitalElectronegativityMulliken of desired atom

* Atom_PiOrbitalElectronegativityPauling : Retrieves the PiOrbitalElectronegativityPauling of desired atom

* Atom_PiValenceStateElectronAffinity : Retrieves the PiValenceStateElectronAffinity of desired atom

* Atom_PiValenceStateIonizationPotential : Retrieves the PiValenceStateIonizationPotential of desired atom

* Atom_Polarizability : computes the polarizability of each atom using the method from see J.Am.Chem.Soc. Vol 112,

No. 23, 1990, 8534

* Atom_SigmaCharge : uses PEOE to determine sigma-orbital partial charge

* Atom_SigmaEN : uses PEOE to determine sigma-orbital electronegativity

* Atom_SigmaOrbitalElectronegativityMulliken : Retrieves the SigmaOrbitalElectronegativityMulliken of desired atom

* Atom_SigmaOrbitalElectronegativityPauling : Retrieves the SigmaOrbitalElectronegativityPauling of desired atom

* Atom_SigmaValenceStateElectronAffinity : Retrieves the SigmaValenceStateElectronAffinity of desired atom

* Atom_SigmaValenceStateIonizationPotential : Retrieves the SigmaValenceStateIonizationPotential of desired atom

* Atom_Stereocenters : 1 for R, -1 for S, 0 for achiral atoms, 2 for undefined chirality

* Atom_TernaryHBond : User-defined. Alias for :

Subtract(lhs=Atom_HbondAcceptors,rhs=Multiply(Constant(2),Atom_HbondDonors))

* Atom_TopologicalPolarSurfaceArea : see Ertl, et. al. J. Med. Chem. 2000, 43, 3715

* Atom_TotalCharge : Returns the total charge on an atom. Alias for : Add(Atom_SigmaCharge,Atom_PiCharge)

* Atom_VDWSurfaceArea : approximates the surface area of atoms using the vdw radius, considering overlap from

neighboring atoms. Alias for : Atom_SurfaceArea(radius=Atom_VDWaalsRadius,min radius=Atom_CovalentRadius)

* Atom_VDWVolume : approximates the volume of atoms using the vdw radius, considering overlap from neighboring

atoms. Alias for : Atom_Volume(radius=Atom_VDWaalsRadius,min radius=Atom_CovalentRadius)

* Atom_VDWaalsRadius : Retrieves the elemental VDWaalsRadius for each atom

* Atom_Vcharge : Partial charges computed using vcharge 2003 algorithm and parameters, see

http://pubs.acs.org/doi/full/10.1021/ci034148o

* Atom_VchargeV2 : Partial charges computed using vcharge 2003 algorithm and parameters, see

http://pubs.acs.org/doi/full/10.1021/ci034148o

* AtomicNumbers : Retrieves the atomic number of each atom

* ChargeNegative : User-defined. Alias for : Multiply(Less(lhs=Atom_SigmaCharge,rhs=Constant(0)),Atom_SigmaCharge)

* ChargePositive : User-defined. Alias for :

Multiply(Greater(lhs=Atom_SigmaCharge,rhs=Constant(0)),Atom_SigmaCharge)

* ENegOffset : User-defined. Alias for : Subtract(lhs=Atom_ElectroNegativity,rhs=Constant(2.5))

* HbondAcceptorsStrict : User-defined. Alias for :

Multiply(Atom_HbondAcceptors,Not(Atom_HbondDonors),LessEqual(lhs=BondTypeCount(property=Identity,value=1),rhs=Constant(2)))

* IsB : Returns 1 for boron atoms, 0 for others. Alias for : Equal(AtomicNumbers,Constant(5))

* IsBr : Returns 1 for bromine atoms, 0 for others. Alias for : Equal(AtomicNumbers,Constant(35))

* IsC : Returns 1 for carbon atoms, 0 for others. Alias for : Equal(AtomicNumbers,Constant(6))

* IsCl : Returns 1 for chlorine atoms, 0 for others. Alias for : Equal(AtomicNumbers,Constant(17))

* IsENeg : User-defined. Alias for : Greater(lhs=ENegOffset,rhs=Constant(0))

* IsF : Returns 1 for fluorine atoms, 0 for others. Alias for : Equal(AtomicNumbers,Constant(9))

* IsH : Returns 1 for hydrogen atoms, 0 for heavy atoms. Alias for : Less(lhs=AtomicNumbers,rhs=Constant(1.5))

* IsHTernary : User-defined. Alias for : Add(Constant(-1),Multiply(IsH,Constant(2)))

* IsHalogen : Returns 1 for atoms in main group 7 (F,Cl,Br,I,At,Uus). Alias for : Equal(Atom_MainGroup,Constant(7))

* IsI : Returns 1 for iodine atoms, 0 for others. Alias for : Equal(AtomicNumbers,Constant(53))

* IsN : Returns 1 for nitrogen atoms, 0 for others. Alias for : Equal(AtomicNumbers,Constant(7))

* IsNotC : Returns 1 for non-carbon atoms, 0 for others. Alias for : NotEqual(AtomicNumbers,Constant(6))

* IsNotH : Returns 1 for heavy atoms, 0 for hydrogen atoms. Alias for :

Greater(lhs=AtomicNumbers,rhs=Constant(1.5))

* IsO : Returns 1 for oxygen atoms, 0 for others. Alias for : Equal(AtomicNumbers,Constant(8))

* IsP : Returns 1 for phosphorus atoms, 0 for others. Alias for : Equal(AtomicNumbers,Constant(15))

* IsPeriodThreePlus : Returns 1 for all elements in periods 3-7. Alias for :

Greater(lhs=AtomicNumbers,rhs=Constant(10.5))

* IsS : Returns 1 for sulfur atoms, 0 for others. Alias for : Equal(AtomicNumbers,Constant(16))

* IsSi : Returns 1 for silicon atoms, 0 for others. Alias for : Equal(AtomicNumbers,Constant(14))

* PolarTernary : User-defined. Alias for :

Subtract(lhs=Multiply(Add(HbondAcceptorsStrict,Atom_HbondDonors),Constant(2)),rhs=Constant(1))

* Position : Returns the X,Y,Z coordinates of the Atom

Customizable Implementations

* Atom_RelativePropertyScore : Compute per-atom contribution of QSAR score relative to a provided scaffold

Default label : Atom_RelativePropertyScore(property=XLogP,reference_mols="")

Parameters:

<property> the property to use for scoring, default: "XLogP",

Calculates LogP using a multi-tasking deep neural network trained to predict LogP, LogS, and hydration

free energy

<reference_mols> the reference molecule(s) against which target molecules will be scored, default: "", any string

* BondTypeCount : Counts the number of bonds that satisfy a condition (property == value)

Default label : BondTypeCount(property=Identity,value=1)

Parameters:

<property> bond property to query, default: "Identity",

Allowed values: {Identity, BondOrder, NumberOfElectrons, Conjugation, IsConjugated, IsAromatic, IsAmide,

IsInRing, BondOrderInRingOrAromatic, BondOrderOrAromatic, BondOrderAmideOrAromatic,

BondOrderOrAromaticWithRingness, BondOrderAmideOrAromaticWithRingness,

FuzzyBondOrderWithIsometryOrAromaticWithRingness, FuzzyBondOrderAmideOrAromaticWithRingness,

ConstitutionalBondType, BondOrderWithIsometry, Isometry, IsIsometric, BondOrderWithIsometryOrAromatic,

BondOrderAmideWithIsometryOrAromaticWithRingness, ConfigurationalBondType}

<value> value to calculate number of bonds satisfying property == value, default: "1", Any non-negative integer

Operations

* Atom_SurfaceArea : approximates the surface area of the atom, considering neighbor overlap

Default label : Atom_SurfaceArea(radius=Molecule/Atom Numeric Descriptor,min radius=Molecule/Atom Numeric

Descriptor)

Parameters:

<radius> Descriptor that defines the maximum atomic radius, assuming no overlap from neighboring atoms,

choose any implementation of Molecule/Atom Numeric Descriptor (already listed)

<min radius> Descriptor that defines the minimum atomic radius, after accounting for overlap from neighboring

atoms, default: "0", choose any implementation of Molecule/Atom Numeric Descriptor (already listed)

* Atom_Volume : approximates the volume of the atom, considering neighbor overlap

Default label : Atom_Volume(radius=Molecule/Atom Numeric Descriptor,min radius=Molecule/Atom Numeric Descriptor)

Parameters:

<radius> Descriptor that defines the maximum atomic radius, assuming no overlap from neighboring atoms,

choose any implementation of Molecule/Atom Numeric Descriptor (already listed)

<min radius> Descriptor that defines the minimum atomic radius, after accounting for overlap from neighboring

atoms, default: "0", choose any implementation of Molecule/Atom Numeric Descriptor (already listed)

Customizable Operations

* 3DA : computes 3D autocorrelation of a specified atom property

Default label : 3DA(property=Molecule/Atom Numeric Descriptor,step size=1,steps=12,normalized=0)

Parameters:

<property> property over which to calculate the 3D-autocorrelation,

choose any implementation of Molecule/Atom Numeric Descriptor (already listed)

<step size> size of each step in angstroms, default: "1.0", Any decimal (floating-point) value >= 0.01 <= 100

<steps> # of steps/bins (each of size = step size) used in the autocorrelation, default: "12",

Any non-negative integer >= 1 <= 1000000

<normalized> deprecated; no longer used, optional, Any non-negative integer

* ElementHistogram1D : computes a histogram using a single descriptor

Default label : ElementHistogram1D(Molecule/Atom Numeric Descriptor,min=0,max=1,bin

size=1,smoothing=0,catchall=0)

Parameters:

descriptor to use, choose any implementation of Molecule/Atom Numeric Descriptor (already listed)

<min> min value to consider, Any decimal (floating-point) value

<max> max value to consider, Any decimal (floating-point) value

<bin size> size of each bin, Any decimal (floating-point) value >= 0

<smoothing> if non-zero, return a histogram with points smoothed with a gaussian kernel, specifically

Ae^(-BinDistance/SmoothingDistance), where BinDistance is the euclidean distance to center of the given bin,

and A is chosen such that the histogram's sum still equals 1, default: "0", Any decimal (floating-point) value >= 0

<catchall> If set, values that fall outside the boundaries will be placed into the nearest bin,

default: "False", Any non-negative integer

* ElementHistogram2D : computes a binary histogram using different descriptors for X and Y axis

Default label : ElementHistogram2D(x=Molecule/Atom Numeric Descriptor,y=Molecule/Atom Numeric Descriptor,min

x=0,min y=0,max x=1,max y=1,bin size x=1,bin size y=1,smoothing=0,catchall=0)

Parameters:

<x> descriptor to use for the x-axis,

choose any implementation of Molecule/Atom Numeric Descriptor (already listed)

<y> descriptor to use for the y-axis,

choose any implementation of Molecule/Atom Numeric Descriptor (already listed)

<min x> min value to plot for the x-axis, Any decimal (floating-point) value

<min y> min value to plot for the y-axis, Any decimal (floating-point) value

<max x> max value to plot for the x-axis, Any decimal (floating-point) value

<max y> max value to plot for the y-axis, Any decimal (floating-point) value

<bin size x> bin size for the x-axis, Any decimal (floating-point) value >= 0

<bin size y> bin size for the y-axis, Any decimal (floating-point) value >= 0

<smoothing> if non-zero, return a grid with points smoothed with a gaussian kernel, specifically

Ae^(-BinDistance/SmoothingDistance), where BinDistance is the euclidean distance to center of the given bin,

and A is chosen such that the histogram's sum still equals 1, default: "0", Any decimal (floating-point) value >= 0

<catchall> If set, values that fall outside the histogram boundaries will be placed into the nearest bin,

default: "False", Any non-negative integer

* PlanarityAtoms : Returns the chi-squared value of the set of atoms from a perfect plane

(anonymous) parameter: coordinate retrievers; must each return 3 values per atom,

Container with at least 4 choose any implementation of Molecule/Atom Numeric Descriptor (already listed)

* RDF : computes the radial distribution function using a given atom property

Default label : RDF(property=Molecule/Atom Numeric Descriptor,step size=0.1,temperature=0,steps=48,normalized=0)

Parameters:

<property> property over which to calculate the radial distribution function,

choose any implementation of Molecule/Atom Numeric Descriptor (already listed)

<step size> size of each step in angstroms, default: "0.25", Any decimal (floating-point) value >= 0.01 <= 100

<temperature> increasing temperature spreads autocorrelation across more distant bins, default: "100",

Any decimal (floating-point) value >= 0 <= 1000

<steps> # of steps/bins (each of size = step size) used in the radial distribution function, default: "48",

Any non-negative integer >= 1 <= 1000000

<normalized> deprecated; no longer used, optional, Any non-negative integer

* RDFGrid : computes the radial distribution function using a given atom property, see

http://www.opus.ub.uni-erlangen.de/opus/volltexte/2007/736/pdf/MarkusHemmerDissertation.pdf, p. 65 for details

Default label : RDFGrid(property=Molecule/Atom Numeric Descriptor,weight property=Molecule/Atom Numeric

Descriptor,distance steps=24,property steps=12,distance step size=0.5,property step size=0.5,distance

temperature=100,property temperature=100)

Parameters:

<property> property over which to calculate the radial distribution function,

choose any implementation of Molecule/Atom Numeric Descriptor (already listed)

<weight property> property whose product will weight the radial distribution function,

choose any implementation of Molecule/Atom Numeric Descriptor (already listed)

<distance steps> number of distance bins (each of size distance size) used in the radial distribution function,

default: "24", Any non-negative integer >= 1 <= 1000000

<property steps> number of property bins (each of size property step size) used in the radial distribution

function, default: "12", Any non-negative integer >= 1 <= 1000000

<distance step size> size of each step for the distance axis in angstroms, default: "0.5",

Any decimal (floating-point) value >= 0.01 <= 100

<property step size> size of each step for the property axis in angstroms, default: "0.5",

Any decimal (floating-point) value >= 0.01 <= 10000

<distance temperature> increasing temperature spreads autocorrelation across more distant bins, default: "100",

Any decimal (floating-point) value >= 0 <= 1000

<property temperature> same as distance temperature but used to distribute values of atom property over more

distant bins, default: "100", Any decimal (floating-point) value >= 0 <= 1000

* RDFSign : computes the radial distribution function using a given atom property

Default label : RDFSign(property=Molecule/Atom Numeric Descriptor,step size=0,temperature=0,steps=0,normalized=0)

Parameters:

<property> property over which to calculate the radial distribution function,

choose any implementation of Molecule/Atom Numeric Descriptor (already listed)

<step size> size of each step in angstroms, default: "0.25", Any decimal (floating-point) value >= 0.01 <= 100

<temperature> increasing temperature spreads autocorrelation across more distant bins, default: "100",

Any decimal (floating-point) value >= 0 <= 1000

<steps> # of steps/bins (each of size = step size) used in the radial distribution function, default: "48",

Any non-negative integer >= 1 <= 1000000

<normalized> deprecated, no longer has any effect, optional, Any non-negative integer

*********************************************************************************************************************

********************** Descriptors that can be natively computed for both Molecule and Atom *************************

*********************************************************************************************************************

By default, these are computed for each Atom, except when using GenerateDataset with a Molecule-level result

descriptor. Molecule-level descriptors can be obtained instead by using MoleculeSum(X), where X is any descriptor listed below

Customizable Operations

* 2DA : computes 2D (bond distance) autocorrelation of a specified atom property

Default label : 2DA(steps=12,property=Molecule/Atom Numeric Descriptor,normalized=1)

Parameters:

<steps> # of steps; corresponds to maximum # of bonds between atoms that will be considered for autocorrelation,

default: "11", Any non-negative integer >= 1 <= 1000000

<property> property over which to calculate the 2D-autocorrelation, default: "Atom_Identity",

choose any implementation of Molecule/Atom Numeric Descriptor (already listed)

<normalized> deprecated; no longer used, optional, Any non-negative integer

* 2DASign : computes 2D (bond distance) autocorrelation of a specified atom property

Default label : 2DASign(property=Molecule/Atom Numeric Descriptor,steps=12)

Parameters:

<property> property over which to calculate the smooth radial distribution function,

choose any implementation of Molecule/Atom Numeric Descriptor (already listed)

<steps> # of steps/bins (each of size = step size) used in the radial distribution function, default: "48",

Any non-negative integer >= 1 <= 1000000

* 2DASmoothSign : computes 2D (bond distance) autocorrelation of a specified atom property

Default label : 2DASmoothSign(property=Molecule/Atom Numeric Descriptor,steps=12,temperature=100)

Parameters:

<property> property over which to calculate the smooth radial distribution function,

choose any implementation of Molecule/Atom Numeric Descriptor (already listed)

<steps> # of steps/bins (each of size = step size) used in the radial distribution function, default: "48",

Any non-negative integer >= 1 <= 1000000

<temperature> increasing temperature spreads autocorrelation across more distant bins, default: "100",

Any decimal (floating-point) value >= 0 <= 1000

* 3daClashSensitiveSign : computes the smooth radial distribution function using a given atom property

Default label : 3daClashSensitiveSign(property=Molecule/Atom Numeric Descriptor,step size=1,steps=12,max

clashes=2,clash distance=0.25)

Parameters:

<property> property over which to calculate the smooth radial distribution function,

choose any implementation of Molecule/Atom Numeric Descriptor (already listed)

<step size> size of each step in angstroms, default: "0.25", Any decimal (floating-point) value >= 0.01 <= 100

<steps> # of steps/bins (each of size = step size) used in the radial distribution function, default: "48",

Any non-negative integer >= 1 <= 1000000

<max clashes> max # of intervening atoms between considered distance pairs, default: "2",

Any non-negative integer >= 1 <= 1000000

<clash distance> distance between atom pair vector and another atom that will be considered a clash,

default: "0.15", Any decimal (floating-point) value >= 0 <= 1

* 3daSmooth : computes the smooth radial distribution function using a given atom property

Default label : 3daSmooth(property=Molecule/Atom Numeric Descriptor,step

size=1,temperature=100,steps=12,gaussian=1,interpolate=1)

Parameters:

<property> property over which to calculate the smooth radial distribution function,

choose any implementation of Molecule/Atom Numeric Descriptor (already listed)

<step size> size of each step in angstroms, default: "0.25", Any decimal (floating-point) value >= 0.01 <= 100

<temperature> increasing temperature spreads autocorrelation across more distant bins, default: "100",

Any decimal (floating-point) value >= 0 <= 1000

<steps> # of steps/bins (each of size = step size) used in the radial distribution function, default: "48",

Any non-negative integer >= 1 <= 1000000

<gaussian> whether to apply gaussian smoothing to the final curve. If set to false, temperature is ignored,

interpolation is linear, and no gaussian smoothing is performed, default: "True", Any non-negative integer

<interpolate> whether to interpolate values to the two nearest points; if false, all weight will be applied to

the nearest bin, default: "True", Any non-negative integer

* 3daSmoothSign : computes the smooth radial distribution function using a given atom property

Default label : 3daSmoothSign(property=Molecule/Atom Numeric Descriptor,step

size=1,temperature=100,steps=12,gaussian=1,interpolate=1)

Parameters:

<property> property over which to calculate the smooth radial distribution function,

choose any implementation of Molecule/Atom Numeric Descriptor (already listed)

<step size> size of each step in angstroms, default: "0.25", Any decimal (floating-point) value >= 0.01 <= 100

<temperature> increasing temperature spreads autocorrelation across more distant bins, default: "100",

Any decimal (floating-point) value >= 0 <= 1000

<steps> # of steps/bins (each of size = step size) used in the radial distribution function, default: "48",

Any non-negative integer >= 1 <= 1000000

<gaussian> whether to apply gaussian smoothing to the final curve. If set to false, temperature is ignored,

interpolation is linear, and no gaussian smoothing is performed, default: "True", Any non-negative integer

<interpolate> whether to interpolate values to the two nearest points; if false, all weight will be applied to

the nearest bin, default: "True", Any non-negative integer

*********************************************************************************************************************

************************************** General-purpose descriptor operations ****************************************

*********************************************************************************************************************

These can be used for any type of descriptor (Molecule-level, Atom-level, etc, or other general purpose

descriptors)

Basic Implementations

* Atom_Hydrophobic : User-defined. Alias for :

Not(DescriptorSum(Abs(Partial(2DASign(property=PolarTernary,steps=3),indices(1,2,5,8)))))

* Atom_SigmaChargeL : User-defined. Alias for : Limit(Atom_SigmaCharge,max=0.25,min=-0.25)

* Atom_VchargeL : User-defined. Alias for : Limit(Atom_Vcharge,max=0.5,min=-0.5)

Customizable Implementations

* ForEach : Allows creation of a set of descriptors by substituting a user-specified set of values in for a

specific parameter

Default label : ForEach(template="",variable="")

Parameters:

<template> Template descriptor; should contain one or more variables to substitute e.g.

template=3DA(property=X,step size=0.25,number steps=48), any object serialization tree

<variable> Variable name; this name should be unique from any other descriptor used inside this label, any string

<values> Values that the variable will take on for this descriptor. Can be omitted if descriptors parameter is

given, optional, Container any object serialization tree

<descriptors> If the replacement values are descriptors of Molecule, then the descriptors can be taken from any

previously-defined list, or given directly. E.g., if Define(YZ=Combine(Y,Z)), then

ForEach(template=Foo(Bar),variable=Bar,descriptors(YZ,T)) expands to: Combine(Foo(Y),Foo(Z),Foo(T)), optional, Container with at least 1 any object serialization tree

* IterativePrediction : uses pre-trained machine learning model(s) on the given object

Default label : IterativePrediction(storage=bcl::model::RetrieveInterface,lower

layer=bcl::model::RetrieveInterface,iterations=2)

Parameters:

<storage> type of storage for models, choose any implementation of bcl::model::RetrieveInterface:

Customizable Implementations

* File : Storage of trained model on the file system; "File(help)" shows internal options

<lower layer> storage for the predictor that this descriptor's predictions should overwrite. Note that using

this descriptor causes the cache to be modified; such that the original predictions of this model cannot be

returned, choose any implementation of bcl::model::RetrieveInterface (already listed)

<iterations> number of iterations to perform, Any non-negative integer

* IterativePredictionMean : computes the mean prediction of pre-trained machine learning model(s) on the given

object, iteratively substituting the values returned by the sub-model with the values returned by this model

Default label : IterativePredictionMean(storage=bcl::model::RetrieveInterface,lower

layer=bcl::model::RetrieveInterface,iterations=2)

Parameters:

<storage> type of storage for models,

choose any implementation of bcl::model::RetrieveInterface (already listed)

<lower layer> storage for the predictor that this descriptor's predictions should overwrite. Note that using

this descriptor causes the cache to be modified; such that the original predictions of this model cannot be

returned, choose any implementation of bcl::model::RetrieveInterface (already listed)

<iterations> number of iterations to perform, Any non-negative integer

* KohonenMapInfo : Computes distances to all members of one or more kohonen maps. May alternatively/additionally

compute position of an element on the map. If both are computed, output will be in the format: position in

kohonen map 1, distances to nodes of kohonen map 1, position in kohonen map 2, etc.

Default label : KohonenMapInfo(distances=1,positions=0,storage=bcl::model::RetrieveInterface)

Parameters:

<distances> Whether to compute distances to all nodes of the kohonen map, default: "True",

Any non-negative integer

<positions> Whether to return positions of best matching node in each kohonen map, default: "False",

Any non-negative integer

<storage> type of storage for models,

choose any implementation of bcl::model::RetrieveInterface (already listed)

* Mapped :

Note that commas are automatically stripped from the given inputs

Default label : Mapped(file="",key=Molecule/Atom String Descriptor,delimiter= )

Parameters:

<file> file with every line containing a key followed by a value, any existent file

<key> descriptor key (id type) to calculate the key for a given input,

choose any Molecule / Atom String descriptor :

******************************************************************************************************

************************************* Descriptors of Molecules ***************************************

******************************************************************************************************

Basic Implementations

* NElements : The number of Atom in the Molecule

Customizable Implementations

* ConformationIndexInSdfFile : Index of this molecule in an sdf file, searching at the Conformation

level; "ConformationIndexInSdfFile(help)" shows internal options

* ConstitutionIndexInSdfFile : Index of this molecule in an sdf file, searching at the Constitution

level; "ConstitutionIndexInSdfFile(help)" shows internal options

* Define : Define an alias for a given descriptor; "Define(help)" shows internal options

* FileID : ID of this molecule from a File; "FileID(help)" shows internal options

* String : A constant string; "String(help)" shows internal options

******************************************************************************************************

****************************** General-purpose descriptor operations *********************************

******************************************************************************************************

These can be used for any type of descriptor (Molecule-level, Atom-level, etc, or other general

purpose descriptors)

Customizable Implementations

* Cached : Retrieve a string already cached on the molecule; "Cached(help)" shows internal options

* ForEach : Allows creation of a set of descriptors by substituting a user-specified set of values

in for a specific parameter; "ForEach(help)" shows internal options

* Name : Retrieves the name of the molecule; "Name(help)" shows internal options

* ToString : Calculates a numeric descriptor and converts it into a string;

"ToString(help)" shows internal options

Operations

* 1st : For pairwise or higher dimension descriptor generation, selects the result from the 1st

sub-object; "1st(help)" shows internal options

* 2nd : For pairwise or higher dimension descriptor generation, selects the result from the 2nd

sub-object; "2nd(help)" shows internal options

* 3rd : For pairwise or higher dimension descriptor generation, selects the result from the 3rd

sub-object; "3rd(help)" shows internal options

* 4th : For pairwise or higher dimension descriptor generation, selects the result from the 4th

sub-object; "4th(help)" shows internal options

Customizable Operations

* Combine : Array of descriptors; "Combine(help)" shows internal options

* Mapped :

Note that commas are automatically stripped from the given inputs;

"Mapped(help)" shows internal options

* Partial : selects particular values (by index) of another descriptor;

"Partial(help)" shows internal options

<delimiter> Delimiter between the id and key. If not given, the ids must be fixed width, based on the # of

characters as the given key descriptorIf the delimiter is given, the read-in string will be tokenized, such

that repeated spacing is ignored, optional, any letter or character

<default> Value(s) returned if the key is not present in the file, optional,

Container with at least 1 Any decimal (floating-point) value

* Prediction : uses pre-trained machine learning model(s) on the given object

Parameter: <storage> type of storage for models,

choose any implementation of bcl::model::RetrieveInterface (already listed)

* PredictionInfo : Computes prediction-related information, including standard deviation, min, max, and ROC-curve

derived statistics such as the PPV or local-PPV associated with a prediction. Output will be statistics first, in

the order provided; followed by metrics, in the order they are provided

Default label : PredictionInfo(predictor=bcl::model::RetrieveInterface)

Parameters:

<predictor> predictor to obtain derived info from, uses pre-trained machine learning model(s) on the given object

Parameter: <storage> type of storage for models,

choose any implementation of bcl::model::RetrieveInterface (already listed)

<statistics> statistics to compute for the predictions, optional,

Container with at least 1 Allowed values: {Min, Max, Mean, StandardDeviation}

<metrics> ROC-curve based metrics to compute for each model's output. e.g. LocalPPV will give you the likelihood

that a prediction is a true positive, optional,

Container with at least 1 Allowed values: {TPR, FPR, FNR, TNR, Accuracy, Specificity, Precision, Recall, PPV,

Ideal-PPV, Ideal-PPV_FPRelative, NPV, FDR, HitRate, MCC, Enrichment, InformationGainRatio, Cutoff, LocalPPV}

* PredictionMean : computes the mean prediction of pre-trained machine learning model(s) on the given object

Parameter: <storage> type of storage for models,

choose any implementation of bcl::model::RetrieveInterface (already listed)

* ToNumeric : Calculates a numeric descriptor and converts it into a string

Default label : ToNumeric(Molecule/Atom String Descriptor,size=1)

Parameters:

data label for a numeric descriptor,

choose any implementation of Molecule/Atom String Descriptor (already listed)

<size> size of the descriptor, default: "1", Any non-negative integer

Operations

* 1st : For pairwise or higher dimension descriptor generation, selects the result from the 1st sub-object

(anonymous) parameter: descriptor of interest,

choose any implementation of Molecule/Atom Numeric Descriptor (already listed)

* 2nd : For pairwise or higher dimension descriptor generation, selects the result from the 2nd sub-object

(anonymous) parameter: descriptor of interest,

choose any implementation of Molecule/Atom Numeric Descriptor (already listed)

* 3rd : For pairwise or higher dimension descriptor generation, selects the result from the 3rd sub-object

(anonymous) parameter: descriptor of interest,

choose any implementation of Molecule/Atom Numeric Descriptor (already listed)

* 4th : For pairwise or higher dimension descriptor generation, selects the result from the 4th sub-object

(anonymous) parameter: descriptor of interest,

choose any implementation of Molecule/Atom Numeric Descriptor (already listed)

* Abs : Takes the absolute value of a descriptor

(anonymous) parameter: descriptor for which to Takes the absolute value,

choose any implementation of Molecule/Atom Numeric Descriptor (already listed)

* Cos : Takes the cosine of a descriptor

(anonymous) parameter: descriptor for which to Takes the cosine,

choose any implementation of Molecule/Atom Numeric Descriptor (already listed)

* DefineNaN : replaces undefined/NaN values in a descriptor with another value or descriptor

Default label : DefineNaN(Molecule/Atom Numeric Descriptor,replacement=Molecule/Atom Numeric Descriptor)

Parameters:

descriptor whose undefined/NaN values should be replaced,

choose any implementation of Molecule/Atom Numeric Descriptor (already listed)

<replacement> undefined values will be replaced with this property value (usually constants),

default: "Constant(0)", choose any implementation of Molecule/Atom Numeric Descriptor (already listed)

* DescriptorMax : Returns the max of a descriptor

(anonymous) parameter: The descriptor on which to compute the max,

choose any implementation of Molecule/Atom Numeric Descriptor (already listed)

* DescriptorMean : Returns the mean of a descriptor

(anonymous) parameter: The descriptor on which to compute the mean,

choose any implementation of Molecule/Atom Numeric Descriptor (already listed)

* DescriptorMin : Returns the min of a descriptor

(anonymous) parameter: The descriptor on which to compute the min,

choose any implementation of Molecule/Atom Numeric Descriptor (already listed)

* DescriptorRange : Returns the range of a descriptor

(anonymous) parameter: The descriptor on which to compute the range,

choose any implementation of Molecule/Atom Numeric Descriptor (already listed)

* DescriptorStandardDeviation : Returns the standarddeviation of a descriptor

(anonymous) parameter: The descriptor on which to compute the standarddeviation,

choose any implementation of Molecule/Atom Numeric Descriptor (already listed)

* DescriptorSum : Returns the sum of a descriptor

(anonymous) parameter: The descriptor on which to compute the sum,

choose any implementation of Molecule/Atom Numeric Descriptor (already listed)

* Divide : Divides two descriptors (binary true/false)

Default label : Divide(lhs=Molecule/Atom Numeric Descriptor,rhs=Molecule/Atom Numeric Descriptor)

Parameters:

<lhs> argument for the left hand side of the operation,

choose any implementation of Molecule/Atom Numeric Descriptor (already listed)

<rhs> argument for the right hand side of the operation,

choose any implementation of Molecule/Atom Numeric Descriptor (already listed)

* Equal : Equals two descriptors (binary true/false)

(anonymous) parameter: Descriptors to Equal,

Container with 2 choose any implementation of Molecule/Atom Numeric Descriptor (already listed)

* Exp : Takes the exponential of a descriptor

(anonymous) parameter: descriptor for which to Takes the exponential,

choose any implementation of Molecule/Atom Numeric Descriptor (already listed)

* Exponentiate : Exponentiates two descriptors (binary true/false)

Default label : Exponentiate(lhs=Molecule/Atom Numeric Descriptor,rhs=Molecule/Atom Numeric Descriptor)

Parameters:

<lhs> argument for the left hand side of the operation,

choose any implementation of Molecule/Atom Numeric Descriptor (already listed)

<rhs> argument for the right hand side of the operation,

choose any implementation of Molecule/Atom Numeric Descriptor (already listed)

* Greater : Greaters two descriptors (binary true/false)

Default label : Greater(lhs=Molecule/Atom Numeric Descriptor,rhs=Molecule/Atom Numeric Descriptor)

Parameters:

<lhs> argument for the left hand side of the operation,

choose any implementation of Molecule/Atom Numeric Descriptor (already listed)

<rhs> argument for the right hand side of the operation,

choose any implementation of Molecule/Atom Numeric Descriptor (already listed)

* GreaterEqual : GreaterEquals two descriptors (binary true/false)

Default label : GreaterEqual(lhs=Molecule/Atom Numeric Descriptor,rhs=Molecule/Atom Numeric Descriptor)

Parameters:

<lhs> argument for the left hand side of the operation,

choose any implementation of Molecule/Atom Numeric Descriptor (already listed)

<rhs> argument for the right hand side of the operation,

choose any implementation of Molecule/Atom Numeric Descriptor (already listed)

* Less : Lesss two descriptors (binary true/false)

Default label : Less(lhs=Molecule/Atom Numeric Descriptor,rhs=Molecule/Atom Numeric Descriptor)

Parameters:

<lhs> argument for the left hand side of the operation,

choose any implementation of Molecule/Atom Numeric Descriptor (already listed)

<rhs> argument for the right hand side of the operation,

choose any implementation of Molecule/Atom Numeric Descriptor (already listed)

* LessEqual : LessEquals two descriptors (binary true/false)

Default label : LessEqual(lhs=Molecule/Atom Numeric Descriptor,rhs=Molecule/Atom Numeric Descriptor)

Parameters:

<lhs> argument for the left hand side of the operation,

choose any implementation of Molecule/Atom Numeric Descriptor (already listed)

<rhs> argument for the right hand side of the operation,

choose any implementation of Molecule/Atom Numeric Descriptor (already listed)

* Ln : Takes the natural log of a descriptor

(anonymous) parameter: descriptor for which to Takes the natural log,

choose any implementation of Molecule/Atom Numeric Descriptor (already listed)

* Log : Takes the base-10 logarithm of a descriptor

(anonymous) parameter: descriptor for which to Takes the base-10 logarithm,

choose any implementation of Molecule/Atom Numeric Descriptor (already listed)

* MaxIndex : computes the index of maximum value for the given descriptor

(anonymous) parameter: descriptor for which to find the index with the maximum corresponding value,

choose any implementation of Molecule/Atom Numeric Descriptor (already listed)

* MinIndex : computes the index of minimum value for the given descriptor

(anonymous) parameter: descriptor for which to find the index with the minimum corresponding value,

choose any implementation of Molecule/Atom Numeric Descriptor (already listed)

* Mod : Mods two descriptors (binary true/false)

Default label : Mod(lhs=Molecule/Atom Numeric Descriptor,rhs=Molecule/Atom Numeric Descriptor)

Parameters:

<lhs> argument for the left hand side of the operation,

choose any implementation of Molecule/Atom Numeric Descriptor (already listed)

<rhs> argument for the right hand side of the operation,

choose any implementation of Molecule/Atom Numeric Descriptor (already listed)

* Negative : gives the negative of the given argument of a descriptor

(anonymous) parameter: descriptor for which to gives the negative of the given argument,

choose any implementation of Molecule/Atom Numeric Descriptor (already listed)

* Not : 1 if the argument is exactly 0, otherwise returns 0 of a descriptor

(anonymous) parameter: descriptor for which to 1 if the argument is exactly 0, otherwise returns 0,

choose any implementation of Molecule/Atom Numeric Descriptor (already listed)

* NotEqual : NotEquals two descriptors (binary true/false)

(anonymous) parameter: Descriptors to NotEqual,

Container with 2 choose any implementation of Molecule/Atom Numeric Descriptor (already listed)

* OuterProduct : Outer product of two descriptors

Default label : OuterProduct(lhs=Molecule/Atom Numeric Descriptor,rhs=Molecule/Atom Numeric Descriptor)

Parameters:

<lhs> argument for the left hand side of the operation,

choose any implementation of Molecule/Atom Numeric Descriptor (already listed)

<rhs> argument for the right hand side of the operation,

choose any implementation of Molecule/Atom Numeric Descriptor (already listed)

* RankAsc : rank property values in ascending order. e.g. 0.2 0.21 0.5 0.1 -> 1 2 3 0Duplicate values receive the

same rank (averaged) assigned, so 1 1 4 -> 0.5 0.5 2

(anonymous) parameter: descriptor to rank,

choose any implementation of Molecule/Atom Numeric Descriptor (already listed)

* RankDesc : rank property values in descending order. e.g. 0.2 0.21 0.5 0.1 -> 2 1 0 3Duplicate values receive the

same rank (averaged) assigned, so 1 1 4 -> 1.5 1.5 0

(anonymous) parameter: descriptor to rank,

choose any implementation of Molecule/Atom Numeric Descriptor (already listed)

* Rescale : Rescales values relative to the Molecule values, specifically, computes:

(X-MoleculeMean(X))/MoleculeStd(X). In the event that std is 0, returns 0

(anonymous) parameter: descriptor to rescale,

choose any implementation of Molecule/Atom Numeric Descriptor (already listed)

* SetNaNToDefinedDescriptorMean : replaces undefined/NaN values in a descriptor with the DescriptorMean of the

defined values

(anonymous) parameter: descriptor whose undefined/NaN values should be replaced,

choose any implementation of Molecule/Atom Numeric Descriptor (already listed)

* Sin : Takes the sine of a descriptor

(anonymous) parameter: descriptor for which to Takes the sine,

choose any implementation of Molecule/Atom Numeric Descriptor (already listed)

* SortAsc : sort property values in ascending order

(anonymous) parameter: descriptor to sort property values,

choose any implementation of Molecule/Atom Numeric Descriptor (already listed)

* SortDesc : sort property values in descending order

(anonymous) parameter: descriptor to sort property values,

choose any implementation of Molecule/Atom Numeric Descriptor (already listed)

* Sqr : squares the argument of a descriptor

(anonymous) parameter: descriptor for which to squares the argument,

choose any implementation of Molecule/Atom Numeric Descriptor (already listed)

* Sqrt : Takes the square root of a descriptor

(anonymous) parameter: descriptor for which to Takes the square root,

choose any implementation of Molecule/Atom Numeric Descriptor (already listed)

* Subtract : Subtracts two descriptors (binary true/false)

Default label : Subtract(lhs=Molecule/Atom Numeric Descriptor,rhs=Molecule/Atom Numeric Descriptor)

Parameters:

<lhs> argument for the left hand side of the operation,

choose any implementation of Molecule/Atom Numeric Descriptor (already listed)

<rhs> argument for the right hand side of the operation,

choose any implementation of Molecule/Atom Numeric Descriptor (already listed)

Customizable Operations

* Add : Adds two descriptors (binary true/false)

(anonymous) parameter: Descriptors to Add,

Container with between 2 and 1000 choose any implementation of Molecule/Atom Numeric Descriptor (already

listed)

* Combine : Array of descriptors

(anonymous) parameter: descriptors to concatenate,

Container with at least 1 choose any implementation of Molecule/Atom Numeric Descriptor (already listed)

* Limit : limit descriptor values to a specified range

Default label : Limit(Molecule/Atom Numeric Descriptor,max=3.40282e+38,min=-3.40282e+38)

Parameters:

descriptor to limit values of,

choose any implementation of Molecule/Atom Numeric Descriptor (already listed)

<max> maximum value for the limit; higher values will be reduced to this value, default: "3.40282e+38",

Any decimal (floating-point) value

<min> minimum value for the limit; lower values will be reduced to this value, default: "-3.40282e+38",

Any decimal (floating-point) value

* Multiply : Multiplys two descriptors (binary true/false)

(anonymous) parameter: Descriptors to Multiply,

Container with between 2 and 1000 choose any implementation of Molecule/Atom Numeric Descriptor (already

listed)

* Partial : selects particular values (by index) of another descriptor

Default label : Partial(Molecule/Atom Numeric Descriptor,indices="")

Parameters:

descriptor of interest, choose any implementation of Molecule/Atom Numeric Descriptor (already listed)

<indices> desired indices (0-offset) of the descriptor to keep,

Container with at least 1 Any non-negative integer

* Sigmoid : Amplitude / ( 1 + exp(-(x-x_offset)/slope)) + y_offset

Default label : Sigmoid(descriptor=Molecule/Atom Numeric Descriptor,amplitude=1,x offset=0,y

offset=0,x=1,y=0.731059)

Parameters:

<descriptor> the property to consider,

choose any implementation of Molecule/Atom Numeric Descriptor (already listed)

<amplitude> amplitude of the sigmoid, default: "1.0", Any decimal (floating-point) value

<x offset> offset of the sigmoid midpoint along the x-axis, default: "0", Any decimal (floating-point) value

<y offset> Offset of the sigmoid midpoint along the y-axis, default: "0", Any decimal (floating-point) value

<x> Any x along the sigmoid other than x-offset, for which the y-value can be provided to compute the sigmoidal

slope, default: "1", Any decimal (floating-point) value

<y> y on the desired sigmoidal curve, default: "0.731", Any decimal (floating-point) value

* WithinRange : This descriptor takes on a value of 1 if a descriptor's mean value is within a range, or 0

otherwise

Default label : WithinRange(descriptor=Molecule/Atom Numeric Descriptor,begin=nan,end=nan,inclusive=1)

Parameters:

<descriptor> the descriptor to consider,

choose any implementation of Molecule/Atom Numeric Descriptor (already listed)

<begin> beginning of the range, Any decimal (floating-point) value

<end> end of the region where properties will be within range, Any decimal (floating-point) value

<inclusive> whether to include the endpoints in the range, default: "true", Any non-negative integer

* WithinRangeGaussian : Returns a value between 0 and 1 based on where another descriptor's value falls relative to

a given mean value

Default label : WithinRangeGaussian(descriptor=Molecule/Atom Numeric Descriptor,mean=0,stdev=1)

Parameters:

<descriptor> the property to consider,

choose any implementation of Molecule/Atom Numeric Descriptor (already listed)

<mean> the mean of the gaussian function, Any decimal (floating-point) value

<stdev> the standard deviation of the gaussian function, Any decimal (floating-point) value

* WithinRangeSmooth : This descriptor gives values between 0.0 and 1.0 depending on if another descriptor's

magnitude is within a specified range. This descriptor's value decreases smoothly as calculated by a sinusoidal

transition (sigmoid-like)

Default label : WithinRangeSmooth(descriptor=Molecule/Atom Numeric Descriptor,begin=0,end=0,left width=0,right

width=0,inclusive=1)

Parameters:

<descriptor> the property to consider,

choose any implementation of Molecule/Atom Numeric Descriptor (already listed)

<begin> the beginning of the range where this descriptor will take on a value of 1.0,

Any decimal (floating-point) value

<end> the end of the range where this descriptor will take on a value of 1.0, Any decimal (floating-point) value

<left width> how wide the interpolation region should be to the left of the beginning of the range,

default: "0", Any decimal (floating-point) value >= 0

<right width> how wide the interpolation region should be to the right of the end of the range, default: "0",

Any decimal (floating-point) value >= 0

<inclusive> whether the ends of the ranges should be inclusive. practically, this only effects a ranges that

have interpolation width(s) of zero, default: "true", Any non-negative integer

Other strings will be interpreted as follows:

* : given a number, returns that number, otherwise retrieves the value of the misc property by that name;

"(help)" shows internal options

choose any Molecule / Atom String descriptor :

*********************************************************************************************************************

******************************************** Descriptors of Molecules ***********************************************

*********************************************************************************************************************

Basic Implementations

* NElements : The number of Atom in the Molecule

Customizable Implementations

* ConformationIndexInSdfFile : Index of this molecule in an sdf file, searching at the Conformation level

(anonymous) parameter: Filenames for all sdf files to consider; returned index indicates the index within the

files as if they were concatenated, Container with at least 1 Retrieves molecules from a file

(anonymous) parameter: Filename, any existent file

* ConstitutionIndexInSdfFile : Index of this molecule in an sdf file, searching at the Constitution level

(anonymous) parameter: Filenames for all sdf files to consider; returned index indicates the index within the

files as if they were concatenated, Container with at least 1 Retrieves molecules from a file

(anonymous) parameter: Filename, any existent file

* Define : Define an alias for a given descriptor

(anonymous) parameter: The LHS of the = sign will become an alias that can be used later (in the same file or

command) to refer to the RHS. Existing aliases cannot be overridden. Accepts , any object serialization tree

* FileID : ID of this molecule from a File

(anonymous) parameter: Filename, Any of the following:

file pattern <*.sdf>

Or file pattern <*.sdf.bz2>

Or file pattern <*.sdf.gz>

* String : A constant string

(anonymous) parameter: any string

*********************************************************************************************************************

************************************** General-purpose descriptor operations ****************************************

*********************************************************************************************************************

These can be used for any type of descriptor (Molecule-level, Atom-level, etc, or other general purpose

descriptors)

Customizable Implementations

* Cached : Retrieve a string already cached on the molecule

Default label : Cached(example,size=12)

Parameters:

the name of a property stored on the molecule, default: "", any object serialization tree

<size> number of characters expected. If the string is longer than this, it will be truncated to this length.If

it is shorter, it will be padded with spaces to this number of characters, provided that it exists on the

molecule (otherwise it will be blank), default: "12", Any non-negative integer

* ForEach : Allows creation of a set of descriptors by substituting a user-specified set of values in for a

specific parameter

Default label : ForEach(template="",variable="")

Parameters:

<template> Template descriptor; should contain one or more variables to substitute e.g.

template=3DA(property=X,step size=0.25,number steps=48), any object serialization tree

<variable> Variable name; this name should be unique from any other descriptor used inside this label, any string

<values> Values that the variable will take on for this descriptor. Can be omitted if descriptors parameter is

given, optional, Container any object serialization tree

<descriptors> If the replacement values are descriptors of Molecule, then the descriptors can be taken from any

previously-defined list, or given directly. E.g., if Define(YZ=Combine(Y,Z)), then

ForEach(template=Foo(Bar),variable=Bar,descriptors(YZ,T)) expands to: Combine(Foo(Y),Foo(Z),Foo(T)), optional, Container with at least 1 any object serialization tree

* Name : Retrieves the name of the molecule

Parameter: <size> number of characters expected. If the string is longer than this, it will be truncated to this

length.If it is shorter, it will be padded with spaces to this number of characters, provided that it exists on

the molecule (otherwise it will be blank), default: "240", Any non-negative integer

* ToString : Calculates a numeric descriptor and converts it into a string

(anonymous) parameter: data label for a numeric descriptor,

choose any implementation of Molecule/Atom Numeric Descriptor (already listed)

Operations

* 1st : For pairwise or higher dimension descriptor generation, selects the result from the 1st sub-object

(anonymous) parameter: descriptor of interest,

choose any implementation of Molecule/Atom String Descriptor (already listed)

* 2nd : For pairwise or higher dimension descriptor generation, selects the result from the 2nd sub-object

(anonymous) parameter: descriptor of interest,

choose any implementation of Molecule/Atom String Descriptor (already listed)

* 3rd : For pairwise or higher dimension descriptor generation, selects the result from the 3rd sub-object

(anonymous) parameter: descriptor of interest,

choose any implementation of Molecule/Atom String Descriptor (already listed)

* 4th : For pairwise or higher dimension descriptor generation, selects the result from the 4th sub-object

(anonymous) parameter: descriptor of interest,

choose any implementation of Molecule/Atom String Descriptor (already listed)

Customizable Operations

* Combine : Array of descriptors

(anonymous) parameter: descriptors to concatenate,

Container with at least 1 choose any implementation of Molecule/Atom String Descriptor (already listed)

* Mapped :

Note that commas are automatically stripped from the given inputs

Default label : Mapped(file="",key=Molecule/Atom String Descriptor,delimiter= )

Parameters:

<file> file with every line containing a key followed by a value, any existent file

<key> descriptor key (id type) to calculate the key for a given input,

choose any implementation of Molecule/Atom String Descriptor (already listed)

<delimiter> Delimiter between the id and key. If not given, the ids must be fixed width, based on the # of

characters as the given key descriptorIf the delimiter is given, the read-in string will be tokenized, such

that repeated spacing is ignored, optional, any letter or character

<default> Value(s) returned if the key is not present in the file, optional, any string

* Partial : selects particular values (by index) of another descriptor

Default label : Partial(Molecule/Atom String Descriptor,indices="")

Parameters:

descriptor of interest, choose any implementation of Molecule/Atom String Descriptor (already listed)

<indices> desired indices (0-offset) of the descriptor to keep,

Container with at least 1 Any non-negative integer
