# Supplementary material for: Introduction to the BioChemical Library (BCL): An Application-Based Open-Source Toolkit for Integrated Cheminformatics and Machine Learning in Computer-Aided Drug Discovery
Source: Front Pharmacol. 2022 Feb 21;13:833099. doi: 10.3389/fphar.2022.833099 (PMC8899505; doi:10.3389/fphar.2022.833099)
Supplement: Supplementary file 4 [file DataSheet1.docx]

10. Supplementary Materials and Methods

*10.1 Dataset preparation*

This section describes dataset preparation for several datasets used in examples throughout the text. Note that all molecule files (SDF) prior to their use in any example were first cleaned with the BCL molecule:Split and molecule:Filter applications as follows:

bcl.exe molecule:Split \

–implementation Largest \

–input_filenames <any_file.sdf> -output <any_file.largest.sdf> \

bcl.exe molecule:Filter \

–input_filenames <any_file.largest.sdf> \

-output_matched <any_file.clean.sdf> \

-defined_atom_types

Tyrosine kinase inhibitor structure files for gefitinib, afatinib, and osimertinib were originally obtained as PDB files from PDB IDs 4G5J, 4I22, and 4ZAU, respectively. They were protonated and converted to SDF format with OpenBabel (O’Boyle et al., 2011) prior to processing in the BCL.

10.1.1 QSPR solubility prediction

Molecules from multiple previously published databases were combined with BCL molecule:Unique to remove redundant compounds and merge properties:

bcl.exe molecule:Unique \

-input_filenames <molecules.sdf> -output <molecules.unique.sdf> \

-scheduler PThread 4 –compare Configurations

Specifically, we merged configurationally unique molecules from the Guthrie FreeSolv0.51 dataset (1339 compounds) (Mobley and Guthrie, 2014), Lowe et al. (22016 compounds) (Edward W. Lowe et al., 2011), PhysProp (12874 compounds) (Syracuse Research Corporation, 1994), and MoleculeNet (4169 compounds in the lipophilicity set, and 1115 compounds in the Delaney set) (Wu et al., 2018). In total, our dataset consisted of labeled 35874 molecules. We had the following numbers for each result label: LogP – 35113, LogS – 20721, and ΔG_hydration_ – 1339.

10.1.2 Kinase inhibitors and opioid receptors for AD modeling

Two sets of drugs, 82 kinase drugs and 58 opioid receptor orthosteric modulators, were obtained from the DrugBank database (Wishart et al., 2018). Only FDA approved drugs were considered, and only orthosteric modulators of opioid receptors were selected for the opioid drug set. The molecules were neutralized, and their 3D conformations were generated with the BCL, e.g.:

bcl.exe molecule:ConformerGenerator \

-add_h –neutralize -ensemble_filenames <kinase_inhibitors.sdf> \

-conformers_single_file <kinase_inhibitors.3d.sdf> \

-max_iterations 500 –top_models 1 –cluster –generate_3d \

-conformation_comparer SymmetryRMSD 0.25

After being preprocessed, each set of drugs were randomized with BCL molecule:Reorder.

bcl.exe molecule:Reorder \

-input_filenames <kinase_inhibitors.3d.sdf> \

-randomize -output <kinase_inhibitors.3d.random.sdf>

Then, the first five molecules are selected as test set, and the rest were used as the training set.

10.2 Druglikeness model training and validation

Linear regression, decision tree, and neural network classification models were generated for section 3.4 to evaluate the potential for hit optimization on compounds identified in a HTS. Model training data were obtained from the Supplementary Materials of Bickerton et al. (Bickerton et al., 2012). Specifically, we modified their “AZ_chemical_survey.csv” file such that the first 8 columns of the new file are the descriptor columns from the original final, and the final column was a binary 1 for “Yes” or 0 for “No” corresponding to the “DO_CHEMISTRY” column of the original file. This was then converted into a BCL partial binary (.bin) file using descriptor:GenerateDataset. For all comparisons to the QED score we used the weighted QED values in the final column of the “AZ_chemical_survey.csv”.

The learning methods utilized for each model were prepared as follows in the configuration file:

Linear Regression –

learning-method: LinearRegression( \

objective function=RMSD, \

solver=Cholesky(smoothing=0) \

)

Decision tree –

learning-method: DecisionTree( \

objective function=Accuracy, \

partitioner=InformationGain, \

Activity cutoff=0.5, \

nodes core=SplitRating, \

min split=0 \

)

Artificial neural network –

learning-method: 'NeuralNetwork( \

transfer function = Sigmoid, \

weight update = Simple(alpha=0.50,eta=0.05), \

dropout(0.20,0.50), \

objective function = %(objective-function)s, \

scaling=AveStd, steps per update=1, hidden architecture(32), \

balance=True, balance target ratio=0.10, \

shuffle=True, input dropout type=Zero \

)'

Results reported for linear regression and decision tree are the ROC curve for the models trained on all data from the original “AZ_chemical_survey.csv” file. Results reported for the artificial neural network are the ROC curve for the pooled average model across 5-fold randomized cross-validation.

**11. Supplementary Figures**

**Supplementary Figure 1. BCL Runtime performance for loading molecules into memory.** The molecule:Unique application was used to load molecules into memory. Molecules were loaded in serial with one thread. Molecules were loaded as pre-generated 3D conformers in SDF format.

**Supplementary Figure 2. BCL Runtime performance for molecular comparisons as a function of thread count.** The molecule:Compare application was used to compare molecules utilizing a largest common substructure Tanimoto similarity metric. The dataset used for comparison was the 1798 (M1 Muscarinic Receptor) active compound list from Butkiewicz et al.^1^. The comparison created a 187x187 matrix of Tanimoto similarity scores. Molecules were loaded as pre-generated 3D conformers in SDF format.

**Supplementary Figure 3. BCL Runtime performance for generating feature datasets.** The descriptor:GenerateDataset application was used to load molecules into memory and generate feature datasets. Molecules were loaded in serial with one thread or in parallel with six threads. Two distinct feature sets were used from Mendenhall et al.^2^. Molecules were loaded as pre-generated 3D conformers in SDF format.

**Supplementary Figure 4. BCL Runtime performance for randomizing feature datasets.** The pre-generated binary files from Supplementary Figure 3 were loaded into memory using descriptor:GenerateDataset and their rows randomized using the Randomize dataset retriever.

**Supplementary Figure 5. BCL Runtime performance for artificial neural network model training.** Models were trained for each of the randomized datasets from Supplementary Figure 4 using settings from Mendenhall et al.^2^. The datasets used for benchmarking are from Butkiewicz et al.^1^. Briefly, each network contained a feature input layer, a single 32 neuron hidden layer, and an output layer for one result label. The model was trained to classify molecule as active or inactive for a given target. Dropout was set to 5% and 25% for the input and hidden layers, respectively. A total of 25 iterations were performed for each model (additional iterations were not previously found to improve model performance^2^). The batch size (number of training samples between weight updates) is equivalent to the total number of training samples. Each data point is the mean time across a 5x cross-validation. The standard deviation is represented with error bars (however, they may not be perceptible because the min and max standard deviations are 0 and 12 seconds, respectively).

**12. Supplementary Tables**

**Supplementary Table 1. Available application groups and applications in the BCL 4.2.0 release.**

| **Group** | **Application** | **Description** |
| --- | --- | --- |
| *bcl* | Cluster | Hierarchical agglomerative clustering of data |
| *protein* | Compare | Calculates the structural differences between protein models using methods such as RMSD and GDT |
|  | CreateSSEPool | Generates a pool of secondary structure elements (SSEs) used by other protein applications including Fold |
|  | Fold | De-novo protein structure prediction algorithm that assembles a pool of secondary structure elements. Allows for usage of experimental restraints (Formerly known as EMFold) |
|  | FusionProtein | Designs chimeric proteins from a scaffold by fusing multiple donor protein fragments |
|  | PDBConvert | Converts PDBs to/from FASTAs and performs various deterministic manipulations of PDBs |
|  | Score | Scores proteins/PDBs according to BCL energy functions |
| *sequence* | Alignment | Sequence alignment with choice of alignment algorithm, scores, and weights |
| *molecule* | AlignToScaffold | Aligns molecules by substructure |
|  | BuildFragmentLibrary | Builds a library of fragments from starting structures |
|  | BuildRotamerLibrary | Builds a rotamer library from starting structures |
|  | Compare | Compares molecules by spatial, property, fingerprint, or substructural features |
|  | ConformerGenerator | Generates small molecule conformations for ensemble of molecules that are provided |
|  | Coordinates | Performs operations on molecule coordinates. |
|  | ExtractProteinPocket | Extracts binding pocket residues from a protein |
|  | Filter | Filters an ensemble of molecules by a user defined criteria |
|  | GenerateHierarchicalTree | Creates a hierarchical tree for an ensemble of molecules where parent node is immediate substructure of child node |
|  | GenerateRosettaNCAAInstructions | Returns an instructions and input file from which to generate non-canonical amino acids in Rosetta |
|  | MapParams | A helper application to reduce manual labor in NCAA design. It maps atom names from a Rosetta-derived SDF to an AmberTools prepi file |
|  | Mutate | Generates molecules via multiple different mutating schemes |
|  | Properties | Works with string and numeric descriptions of molecules |
|  | React | Performs chemical reactions to produce new molecules. |
|  | Reorder | Reorder atoms in molecules, or reorder molecules in SD files by property |
|  | SetSampleByPartsAtoms | Labels molecules with atom indices to be sampled in SampleConformations based on common substructure to a reference molecule |
|  | Split | Generates molecule fragments via multiple different splitting schemes |
|  | Unique | Removes duplicate molecules at constitution, configuration, conformation, or exact levels |
| *descriptor* | Analyze | Analyzes the Tanimoto overlap between descriptor sets. Formerly known as AnalyzeCodeObjectFile |
|  | ConvertCodeObjectFile | Converts a code object file from an CSV-style formatting version into the new version given a complete set of CSV-formatted descriptors and standard BCL code-object file descriptors. |
|  | DatasetSimilarityMeasures | Generate similarity measures of small molecule feature sets. |
|  | GenerateDataset | Generates bin files (fast, small, easier to use with other applications), csv files (human readable), or .arff files (for use with WEKA) from any combination of dataset sources. Can compute dataset statistics and compare bin files. |
|  | GeneratePCAEigenVectors | Generates descriptor set eigenvectors |
|  | RefineByScore | Creates a new descriptor file using the best descriptors by score. Formerly known as RefineDescriptors |
|  | ScoreDataset | Scores feature columns using various criteria. Used during descriptor development and selection. Formerly known as ScoreFeatureResultDataset |
|  | SequentialFeatureSelection | Takes a descriptor groups file and generates new groups of features to test, based on the best descriptor set in a prior round. Formerly known as TrainModelDescriptorSelection |
| *model* | ComputeStatistics | Evaluates quality measures of QSAR/QSAP model predictions or just experimental/ predicted values, and present results in table and as gnuplot graphics. Formerly known as ComputeJuryStatistics |
|  | PredictionMerge | Merges cross-validation/prediction matrices horizontally or simply append those models. Formerly known as TrainModelPredictionMerge |
|  | Test | Tests any machine learning models. Formerly known as TestModel. |
|  | TestANNWithDropoutResampling | Tests an ANN using dropout at test-time to compute the distribution of values for each output. Formerly known as TestANNWithDropoutResampling. |
|  | Train | Train any machine learning models. Formerly known as TrainModel. |
| *cheminfo* | FocusedLibraryDesign | Generates distributions of molecules utilizing alchemical mutations and a property-based score metric, such as a QSAR model. |
|  | LinkFragments | Connect molecule fragments with various linkers; deprecated in favor of Mutate. |
|  | MoleculeFeatures | Generate absolute and relative pharmacophore/feature maps |
|  | MoleculeFit | Fits molecules against one another utilizing several routines. |
|  | ReactionCombichem | Reaction-based de novo molecule design; deprecated in favor of React. |
| *bioinfo* | ContactPrediction | Predicts amino acid contacts for a given sequence. |
|  | Jufo | Predicts secondary structure for a given protein primary sequence. |
| *restraint* | Pofr | Computes pairwise distance histogram of bcl_model and scale to experimental data. Formerly known as RestraintPofr. |
|  | Saxs | Create SAXS profiles from input PDB file and compare the profile generated with an experimental profile. Formerly known as SimulateSaxsData |
|  | SaxsPrep | Prepares experimental SAXS data for analysis |
| *density* | FitInDensity | Fits atomic protein structure into cryoEM or other medium resolution (5-20 A) density maps. |
|  | FitInDensityMinimize | Protein model refinement using a density map. Optimizes the correlation between the given experimental density and the simulated density. |

**Supplementary Table 2. Common command-line options across BCL applications**

| **General flags** | **Descriptions** |
| --- | --- |
| -help | output user help for the bcl or an application |
| -readme | output readme information for the application |
| -message_level | adjust the MessageLevel  <level> minimum level of messages that will be printed, default: "Standard", Allowed values: Error, Silent, Critical, Standard, Verbose, Debug  <verbosity> set detailing level of printing the source file and line of origination for each message, default: "Summary", Allowed values: Summary, Detail |
| -logger | Set the stream for the output. Default: "Default", Choose from the following: Default (output to screen), File (output to a log file + name of log file) |
| -scheduler | choice of scheduler and number of cpus.  <scheduler> type of scheduler, default: "Serial", Choose from the following: Serial, Pthread (distributes jobs to  <number_cpus> number of cpus for a Pthead scheduler, default: "1". |
| -file_compressed_alternatives | set to enable the search for files that cannot be opened, but for which a file with a compression extension might exist as alternative |
| -file_compression | the type of file compression. Choose from the following: Uncompressed, BZ2, GZ, Crypt. |
| -enums_files | files for enum data that adds enums or overrides data of existing enum data. |
| -random_seed | adjust the random seed. |
| -model_path | path for reading and writing models. |
|  |  |

**Supplementary Table 3. Parameters for applications discussed in in the main text.**

| **Group** | **Application name** | **Descriptions and application specific options** |
| --- | --- | --- |
| Molecule |  | **Molecule group greneral flags:**  **-input_filenames :** input files containing molecules in sdf format, This flag can be followed by any number of files containing molecules in sdf format  **-input_start:** index (0-offset) of first molecule to load from the SDF input file(s)  **-input_max :** Specify the maximum number index of molecules to be loaded from the input sdf file(s)  **-add_h :** saturates all molecules with hydrogen atoms  **-remove_h :** remove hydrogens from molecules when loaded  **-neutralize :** neutralizes any formal charges  **-explicit_aromaticity :** write MDL bonds with aromatic bonds specified explicitly (as 4) |
| (Molecule processing application subgroup) | Filter | Selects a subset of molecules from the input SDF file(s) that satisfy a set of criteria. This application are commonly used to cleanning SDF files or identifying molecules that cannot be easily and automatically corrected  **Output flags:**  **-add_atom_type :** add atom types in mdl line while writing out  **-output_matched :** sdf filename for where to write out molecules that met all the criteria  **-output_unmatched :** sdf filename for where to write out molecules that failed any of the criteria  **Properties related criterion flags:**  **-has_properties :** match molecules that have miscellaneous properties of interest. More than 1 <property> name are allowed.  <property> name of miscellaneous property  **-property_has_string :** match molecules that have miscellaneous properties with string values of interest. This flag can be followed by any number of  miscellaneous <property>  <property> name of miscellaneous property  <target_string> string value that the property should have for matched molecules  **-property_contains_string :** match molecules that have miscellaneous properties with string values contain strings of interest. This flag can be followed by any number of miscellaneous <property>  <property> name of miscellaneous property  <target_string> string that the property value should contain for matched molecules  **-compare_property_values :** Match molecules with property values that satisfy the comparison, This flag can be followed by any number of  <property> name of miscellaneous property  <comparison> Choose from the following: less, less_equal, greater, greater_equal, equal, not_equal  <target_string> string value that the property should have for matched molecules  **Other criterion flags:**  **-3d :** match molecules that have 3d coordinates on at least one neighbor of every atom with 4 neighbors  **-defined_atom_types :** match molecules with no undefined atom types  **-simple :** matches molecules which are simple (e.g. not molecular complexes)  **-only_planar_amide_bonds :** match molecules for which any amide or thioamide bonds outside rings are within a specified number of degrees of planar  <tolerance> number of degrees out of plane to allow amide bonds to be (any number from 0 to 180, default: 20)  **-only_planar_aromatic_rings :** match molecules for which any simple, non-bridged aromatic rings are planar  **-contains_fragments_from :** match molecules with any of the fragments from <contains_fragments_from> file  <contains_fragments_from> filename for input sdf of fragments to find.  <atom_comparison_type> string value of the atomtype info for substructure matching calculation  <bond_comparison_type> string value of the bondtype info for substructure matching calculation  **-contains_conformers :** match molecules with any of the fragments from <contains_conformers> file  <contains_conformers> filename for input sdf of conformers to find.  **-conformation_comparer :** match molecules satisfying criterion of the method for conformation comparison  <rmsd_type> rmsd method to be used for comparing conformations  <comparison> comparison to perform between input molecules and conformers (less, less_equal, greater, greater_equal, equal, not_equal)  <tolerance> amount of tolerance allowed between two conformers, as float value after <comparision>  **-contains :** match molecules with the same constitutional structures as any molecules in the <contains> file  <contains> filename for input sdf of molecules to find.  **-any :** if any of the criteria specified is match, the molecule will be matched (by default, all criteria must match) |
|  | Unique | Identifies duplicate molecules and remove redundant compounds in the input SDF files  **Output Flags:**  **-output :** sdf filename for where to write out molecules  **-output_dupes :** sdf filename for where to write out duplicate molecules  **Comparison method flags:**  **-compare :** Choice of comparison levels to determine duplicate molecules: Constitutions, Configurations, Conformations, and Exact  **-conformation_comparer :** match molecules satisfying criterion of the method for conformation comparison  <rmsd_type> rmsd method to be used for comparing conformations  <comparison> comparison to perform between input molecules and conformers (less, less_equal, greater, greater_equal, equal, not_equal)  <tolerance> amount of tolerance allowed between two conformers, as float value after <comparision>  -merge_descriptors : If true, merge descriptors of the duplicates. Note that this significantly increases memory requirements  **-overwrite_descriptors :** If true, overwrite descriptors of the duplicates.  **-same_molecule_same_name :** If set, only match molecules if they have identical names |
|  | Reorder | Sorts and reorders the molecules based on the criteria that are set by the user.  **Output flags:**  **-output :** sdf filename for where to write out molecules  **-max_molecules :** set the number of molecules in each output file.  **-output_max :** the maximum number of molecules to write out  **-output_order :** fine control over ordering of molecules using an explicit index list. This flag can be followed by a 0-indexed list of how molecules should be reordered  **Molecule reordering flags:**  **-randomize :** randomly reorder the molecules  **-sort :** sort by any property  <property> small molecule property( choose any Molecule / Atom Numeric descriptor)  **-reverse :** reverse the molecules (after sorting, if a sort property was given)  **Atom reordering flags:**  **-canonicalize :** sort atoms in the molecule by descending cahn-ingold-prelog priority (Useful for applications that require that the atoms in the molecule are aligned)  **-atom_order :** Reorder atoms in the given molecules into the order specified. This flag can be followed by a 0-indexed list of how the atoms should be reordered. |
|  | Split | Derives fragments from starting small molecules to aid in pharmacophore modeling, fragment-based drug discovery, and de novo drug design.  **Output flags:**  **-min_frag_size :** set the minimum number of heavy atoms for the output fragements  **-recenter :** translate molecules such that the middle of the molecule is at the origin  **-preserve_mdl_properties :** keep the MDL properties of original molecule on all split components  **-output :** file to write split molecules into  **Splitting method flags:**  **-implementation :** method to split molecules (choose any implementation of bcl::chemistry::FragmentSplitInterface). |
|  | Coordinates | Recenters all molecules in the input file(s) to the origin, computea molecular centroidsm and computes statistics on bond lengths, bond angles, and dihedral angles.  **Ouput Flags:**  **-output :** base name for output of histograms  **Functionality flags:**  **-recenter :** translate molecules such that the middle of the molecule is at the origin  **-centroid :** output geometric coordinates for each molecule in ensemble  **-statistics :** acquire statistics on bond angles, dihedral angles, and bond lengths  **-dihedral_bin_size :** # of degrees each bin will represent in the dihedral angle histograms  **-bond_angle_bin_size :** # of degrees each bin will represent in the bond angle histograms  **Output scoring flags:**  **-dihedral_scores :** dihedral scores are printed alongside the atom indices of the central bond atoms; these scores do not include amide bond non-planarity penalties  **-amide_deviations :** amount of deviation from planarity printed alongside the atom indices of the central bond atoms (units: degrees)  **-amide_penalties :** amide bond penalties; penalty associated with amide bond deviation from planarity; printed alongside the atom indices of the central bond atoms (units: ConfScore units)  **-clash_scores :** atom pair clash scores are printed alongside the atom indices of the clashing atoms; if hydrogen atoms are to be included in clash score, pass 1 |
|  | Properties | Computes molecular descriptors/properties. Properties can be added to an SDF file, renamed, or removed. Statistics and histograms can also be collected for properties.  **Output flags:**  **-output :** sdf filename for where to write out molecules  **-output_histogram :** filename to write out histograms  **-output_table :** filename to write out csv of properties given in -tabulate  **Property flags:**  **-numeric_histogram :** generate a histogram for properties containing numeric values. This flag can be followed by any number of  <property> atom or small molecule property,  <min_value> min/left boundary of the histogram,  <bin_size> size of each bin in the histogram  <number_bins> number of bins in the histogram  **-string_histogram :** properties containing space-delimited strings, This flag can be followed by any number of  <property> property, choose any implementation of bcl::chemistry::StringPropertyInterface.  **-statistics** : properties on which to take statistics. This flag can be followed by any number of properties names.  **-tabulate :** output property statistics into a csv file. This flag can be followed by any number of properties.  **-remove :** remove miscellaneous properties from all molecules. This flag can be followed by any number of properties.  **-remove_all :** removes all properties from all molecules.  **-add :** add properties to all molecules. This flag can be followed by any number of properties.  **-add_strings :** add strings as properties to all molecules. This flag can be followed by any number of names of properties and their assigned strings.  **-rename :** for each pair of properties listed, change the name from the first property to the second. This flag can be followed by any number of properties. |
|  | ConformerGenerator | Samples and generates small molecule 3D conformers.  **Output flags:**  **-conformers_single_file :** output all conformations of ensemble to one output file.  **-conformers_separate_files :** this flag will generate separate sdf file for each ligand.  **-output_ligand_data :** For debugging, output fragments and conformations for every molecules  **-output_rmsd_score :** output a file containing rmsd to native and score of conformers. This flag can be followed by:  <output_rmsd_score> output filename to containing rmsd to natives and score of conformers  <rmsd_type> method to use to find rmsd between conformers, choose any implementation of bcl::chemistry::ConformationComparisonInterface.  **-top_models :** number of conformers to output.  **-failed_3D :** filename base to output molecules whose 3D structures could not be generated.  **-minimum_number_conformations :** if one wants to output a fixed number of conformers.  **Conformation sampling flags:**  **-natives :** sdf file containing the conformations that need to be compared to generated conformers.  **-conformation_comparer :** method to compare conformers (As described in molecule::Filter application).  **-max_iterations :** maximum number of iterations for searching conformations.  **-change_chirality :** specify if chirality and isometry should be sampled as well during conformation sampling.  **-generate_3D :** Use rotamer library instead to generate initial conformation, ignore input structure and bond lengths / angles.  **-skip_cluster :** disable clustering, appropriate when a Boltzmann-like conformer ensemble is desired rather than one that beest covers likely ligand conformational space.  **-rnd_dihedral_mutate_weight :** Relative weight for random mutation of dihedral angles.  **-skip_rotamer_dihedral_sampling :** Skip dihedral sampling, but still allow wiggling dihedrals up to +/- 30 degrees around the input conformation.  **-skip_bond_angle_sampling :** Skip bond angle and bond length sampling.  **-skip_ring_sampling :** Skip ring conformation sampling  **-clash_resolution :** maximum number of tries (times number of dihedral angles plus number of bond angles) to resolve clashes permolecule, before clustering).  **-max_clash_tolerance** : maximum average angstroms clash present across all atoms in the molecule.  **-rotamer_library :** path to rotamer library to use |
|  | Compare | Compute distance/similarity between each molecule in the input SDF file to a reference molecule(s).  **Input file flags:**  **-ensemble_a_start :** Index (0-indexed) of the first molecule to load from the first input SDF file  **-ensemble_b_start :** Index (0-indexed) of the first molecule to load from the second input SDF file  **-ensemble_a_max :** maximum number of molecules to take from the first input SDF file  **-ensemble_b_max :** maximum number of molecules to take from the second input SDF file  **Output file flags:**  **-output :** filename for similarity matrix  **-bcl_table_format :** output the scores in bcl table format  **Similarity analysis flags:**  **-disable_strict_atom_bond_type_checks :** disables strict atom/bond type checking.  **-method :** method for comparing molecules  default: "RMSD", choose any implementation of bcl::chemistry::ConformationComparisonInterface. |
|  | React | Performs chemical reactions to produce new molecules. Currently, intramolecular and partial intramolecular reactions not supported.  **Output file flags:**  **-add_atom_type :** add atom types in mdl line while writing out.  **-output_filename :** file to which the molecules will be output after fitting.  **Reagent and product conformation flags:**  **-corina :** make a system call to the external program Corina to make the final 3D conformer of each product.  **-ligand_based :** overrides 3D conformer settings to just produce an arbitrary conformer without preserving spatial information.  **-sample_confs :** settings for small molecule conformer generation.  **-fix_geometry :** pose-dependent; if 3D conformer matters, fix atoms with bad geometry even if they are in reference structure.  **-fix_ring_geometry :** pose-dependent; if 3D conformer matters, add all ring atoms from non-reference scaffolds to mobile selection.  **-extend_adjacent_atoms :** pose-dependent; include adjacent atoms out this many bonds from any perturbed atom when generating a new 3D conformer.  **Reaction flags:**  **-starting_fragments :** fragments to react with reagents.  **-reagents :** reagents to react with starting fragments.  **-reactions :** directory containing reaction files.  **-routine :** the reaction routine to perform: Random (perform a random reaction on each starting fragment) or Exhaustive (perform all reactions on each starting fragment).  **-repeats :** the number of times to repeat a reaction routine for a given 'starting_fragment'.  **-allowed_reactant_positions :** reactant position indices (0-indexed) indicating which parts of the reaction(s) the target molecule is allowed to match. |
| Descriptor | GenerateDataset | Generates, manipulates, and analyzes feature datasets for QSAR/QSPR models.  **I/O flags:**  **-source :** method to retrieve the dataset. A source could be any implementation of bcl::model::RetrieveDataSetBase (a raw or preprocessed Csv, SDF , or binary file).  **-feature_labels :** label or file containing the label for the feature descriptors.  **-result_labels :** label or file containing the label for the result descriptors.  **-id_labels :** label or file containing the id label.  **-output :** filename for output dataset with suffix .bin (binary) or .csv or .arff (for use with WEKA).  **-block_size :** number of MB in the output dataset to generate before writing out.  **Dataset processing flags:**  **-compare :** filenames to compare generated output bin file with, This flag can be followed by any number of bin file names.  **-nonredundant :** Allows dataset to be post-filtered to remove redundant descriptors; criteria for which can be set with options:  <max outliers> Maximum number of data points that can violate the z-score tolerance for a particular descriptor before declaring a pair of descriptors non-redundant  <tol> Maximum difference in z-score between two redundant descriptors.  <allow a constant> allow up to one constant, non-zero, descriptor in a dataset.  <min span> Minimum range of values that a descriptor needs to span to be considered non-constant.  <min std> Minimum standard deviation of values that a descriptor needs to span to be considered non-constant.  <min rsq> Minimum r-squared for declaration of feature as redundant.  **-forbid_incomplete_records :** whether to forbid records that have incomplete results (some, but not all, results nan). |
| Model | Train | Trains machine models  **I/O flags:**  **-training :** source of dataset used to train the model (a CSV/binary/SDF file, or a preprocessed CSV/binary/SDF file)  **-monitoring :** source of dataset used when deciding whether model has improved.  **-independent :** source of dataset used when evaluating the final objective function.  **-feature_labels :** label or file containing the label for the feature descriptors.  **-result_labels :** label or file containing the label for the result descriptors.  **-id_labels :** label or file containing the label for the id descriptors.  **-print_training_predictions :** print the predicted values for training data to (1st column = actual, 2nd column = predicted) to a file.  **-suppress_progress_output :** by default, objective function evaluations are shown as the model is training; set this flag to suppress that output.  **-print_monitoring_predictions :** file to print the predicted values for monitoring data to (1st column = actual, 2nd column = predicted).  **-print_independent_predictions :** file to print the predicted values for independent data to (1st column = actual, 2nd column = predicted).  **-storage_model :** location to store the trained models.  **-storage_descriptor_selection :** location to store meta data (used in descriptor selection).  **Training flags:**  **<machine learning algorithms>** choose any implementation of bcl::model::ApproximatorBase  **-max_minutes :** maximum # of minutes to train.  **-max_iterations :** maximum number of iterations.  **-max_unimproved_iterations :** maximum number of iterations that can pass between improvement steps without stopping the training.  **-result_averaging_window :** Window size for computing the current average result, helps smooth noisy objective functions.  **-final_objective_function :** data label for an objective function the evaluates the final model.  **-continued_training :** write out a serialized iterate instance for later continuation of training.  **-continue :** read in an iterator for training. |
|  | PredictionMerge | Reads in multiple prediction outputs of a trained machine learning models and merges or appends these into one prediction outputs. The functionality is beneficial for cross-validation use case scenarios.  **I/O flags:**  **-input :** input prediction matrices.  **-input_model_storage :** Use a model storage to compute all the input filenames, which to merge, which to append.  **-output :** output filename for final prediction matrix  **Prediction merging flags:**  **-modus :** flag for appending (flag is set) or merging (flag is not set) prediction matrices.  **-median :** flag computing median prediction prediction matrices.  **-jury :** flag computing jury prediction based on an objective function.  **-min :** flag computing min prediction matrices  **-max :** flag computing max prediction matrices  **-local_ppv :** average local PPV values instead of raw model outputs |
|  | ComputeStatistics | Reads in the prediction output of a trained machine learning model and evaluates an array of quality measures and objective functions to determine the prediction performance of the trained model.  **I/O flags:**  **-input :** list of input filenames  **-take_log10 :** takes log base 10 of the input values  **-filename_obj_function** : file where all specified objective function evaluation are stored.  **-output_directory :** directory where the output will be re-directed.  **Data visualization flags:**  **-plot_x :** choose which contingency matrix measure to plot on the x-axis.  **-plot_log_x :** choose which contingency matrix measure to plot on the log x-axis.  **-plot_y :** choose which contingency matrix measure to plot on the y-axis  **-no_plot :** disable plotting or writing out data files for each output.  **-correlation :** indicates that correlation plots and not ROC curves are desired  **-potency_cutoff :** potency cutoff for plotting roc curves  **-image_format :** specify the image type to display  **Model evaluation flags:**  **-table_name :** name of the table containing the quality measures.  **-sort_by :** the quality measure to sort the table by.  **-actives_below_cutoff :** indicates that those values predicted below cutoff are treated as actives  **-obj_function :** list of objective function labels. |
|  | Test | tests any machine learning model generated with the BCL.  **I/O flags:**  **-retrieve_dataset :** method to retrieve the dataset to test the model(s) against (a CSV/binary/SDF file, or a preprocessed CSV/binary/SDF file).  **-storage_model :** location of models to predict with.  **-id_labels :** label or file containing the id label.  **-output :** flag for output base name.  **Computing flags:**  **-average :** set this flag to only write out the average file (to -output), rather than files for each model |

**References**

Bickerton, G. R., Paolini, G. V., Besnard, J., Muresan, S., and Hopkins, A. L. (2012). Quantifying the chemical beauty of drugs. *Nat. Chem.* 4, 90–8. doi:10.1038/nchem.1243.

Edward W. Lowe, Mariusz Butkiewicz, Matthew Spellings, Albert Omlor, and Jens Meiler (2011). Comparative Analysis of Machine Learning Techniques for the Prediction of LogP.

Mobley, D. L., and Guthrie, J. P. (2014). FreeSolv: a database of experimental and calculated hydration free energies, with input files. *J. Comput. Aided Mol. Des.* 28, 711–720. doi:10.1007/s10822-014-9747-x.

O’Boyle, N. M., Banck, M., James, C. A., Morley, C., Vandermeersch, T., and Hutchison, G. R. (2011). Open Babel: An open chemical toolbox. *J. Cheminform.* 3, 33. doi:10.1186/1758-2946-3-33.

Syracuse Research Corporation (1994). Physical/Chemical Property Database (PHYSPROP).

Wishart, D. S., Feunang, Y. D., Guo, A. C., Lo, E. J., Marcu, A., Grant, J. R., et al. (2018). DrugBank 5.0: a major update to the DrugBank database for 2018. *Nucleic Acids Res.* 46, D1074–D1082. doi:10.1093/nar/gkx1037.

Wu, Z., Ramsundar, B., Feinberg, E. N., Gomes, J., Geniesse, C., Pappu, A. S., et al. (2018). MoleculeNet: a benchmark for molecular machine learning. *Chemical Science* 9, 513–530. doi:10.1039/c7sc02664a.
